# Hydrogenation of aromatic ketones, aldehydes, and epoxides with hydrogen and Pd(0)EnCat™ 30NP

**DOI:** 10.1186/1860-5397-2-15

**Published:** 2006-08-25

**Authors:** Steven V Ley, Angus J P Stewart-Liddon, David Pears, Remedios H Perni, Kevin Treacher

**Affiliations:** 1 Department of Chemistry, University of Cambridge, Lensfield Road, Cambridge CB2 1EW, UK; 2 Reaxa Ltd, Hexagon Tower, Blackley, Manchester M9 8ZS, UK

## Abstract

Aromatic aldehydes and ketones as well as aromatic epoxides are selectively reduced to the corresponding alcohols under mild conditions using conventional hydrogen in the presence of Pd(0)EnCat™ 30NP catalyst. The reactions were performed at room temperature during 16 hours with high to excellent conversions of the corresponding products.

## Introduction

Benzylic alcohols occupy an important position in organic synthesis as target molecules of biological interest and synthetic intermediates that can be produced from the reduction of aromatic carbonyl substrates. Many reducing agents have been used for this purpose including metallic hydrides (NaBH_4_, LiAlH_4_) and their derivatives, in addition to borane and substituted boranes, which are the most commonly used. [1–4] However, from a practical point of view a more attractive approach is to develop a selective heterogeneous catalyst that is efficient for this transformation. From an industrial perspective the economical and environmental impact of processes which replace wasteful complex hydride reagents with more weight efficient reductants such as hydrogen is considerable. Selective hydrogenation of these compounds to the corresponding alcohols is complicated by the fact that different side reactions can take place like aromatic ring hydrogenation, as well as hydrogenolysis of the produced alcohol to the hydrocarbon derivative. [5–6] Some of these problems have been circumvented by adding ethylenediamine, [7] although this can make isolation of the product and recycling of the catalyst problematic. Addition of NaOH or designing bimetallic catalysts in which one of the metals acts as poisoning agent, have also been reported. [8–10]

Several metals (e.g. Pd, Pt, Ru) have been used in the literature for the selective heterogeneous catalytic reduction of aryl ketones and aldehydes to the corresponding alcohols. These catalysts generally involve the use of additives to avoid the secondary reactions mentioned above, and/or the use of toxic mixed metals and in many cases high temperatures are required. [11–14]

In previous work, colleagues at Cambridge University described the selective reduction of electron deficient and neutral aryl ketones to benzylic alcohols using Pd(0)EnCat™ 30NP as catalyst and a mixture of HCOOH/Et_3_N as the source of hydrogen [16]. Under these conditions they also achieved the chemoselective hydrogenolysis of aryl epoxides [17]. For these kind of substrates, catalysts based on Ni, Pd, and Pt have been used, and further efforts are directed toward improving the chemoselectivity and regioselectivity of this ring-opening reaction. [18–19]

We now demonstrate that aryl aldehydes and ketones as well as aryl epoxides can also effectively be reduced using Pd(0)EnCat™ 30NP under conventional catalytic hydrogenation conditions of H_2_ (atmospheric pressure) with good selectivity and conversions [20].

## Results and discussion

The results obtained using different conditions for the carbonyl reduction of 4-methoxybenzaldehyde and 4-methoxyacetophenone are summarized in Table 1. In the first case, when Pd/C was used the over-reduced product was quantitatively obtained (entries 7 and 8). Using palladium on different supports with lower surface area than carbon, such as CaCO_3_ or Al_2_O_3_ (entries 4 and 5) a mixture of benzylic alcohol and over-reduced product was found. However, a much better selectivity was achieved when using Pd(0)EnCat™ 30NP. In this case, the corresponding alcohol was obtained in 94%–95% conversion (entries 1, 2, and 3). It is known that the use of non-protic solvents like ethyl acetate can avoid the over-reduction of aryl aldehydes but in our experiments the same selectivity was obtained in ethanol and ethyl acetate. We have also seen minor difference in reactivity between water wet or solvent prewashed EnCat™. Solvent prewashed catalyst is slightly less active and so more selective, giving less of the over-reduced side product (entry 3). It is worth mentioning that the crude product obtained in Table 1, Entry 1 was analysed for metal contamination to determine the exent of leaching. Inductively coupled plasma (ICP) analysis indicated the level of palladium (in the reaction mixture after filtration) to be under 6 ppm.

**Table 1:** Pd(0)EnCat™ 30NP-catalyzed hydrogenation of 4-mathoxybenzaldehyde and 4-methoxyacetophenone. Comparison with other Pd catalysts.

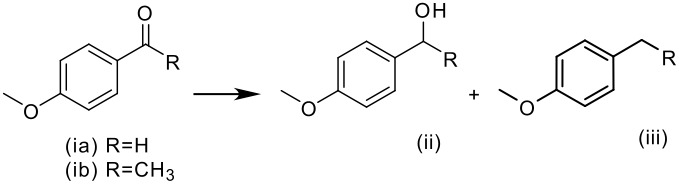					
**Entry**	**Method^a^**	**R**	**Solvent**	**Conversion^b^**	
				**(ii)%**	**(iii)%**

1	H_2_, Pd(0)EnCat™ 30NP	H	EtOH	94	6
2	H_2_, Pd(0)EnCat™ 30NP	H	AcOEt	94	6
3	H_2_, Pd(0)EnCat™ 30NP pre-washed with EtOH	H	EtOH	95	5
4	H_2_, 5% Pd/CaCO_3_^c^	H	EtOH	63	37
5	H_2_, 5% Pd/Al_2_O_3_^d^	H	EtOH	45	55
6	H_2_, 10% Pd/C Engelhard^e^	H	EtOH	13	84
7	H_2_, 5% Pd/C Aldrich^f^	H	EtOH	---	100
8	H_2_, 5% Pd/C J.Matthey^g^	H	EtOH	---	100
9	H_2_, Pd(0) EnCat™ 30NP	CH_3_	EtOH	100	---

^a^ Reaction conditions: H_2_ Balloon or HCO_2_H/Et_3_N, 10 mol% Pdcatalyst, r.t., 16 hours. Pd(0)EnCat™ 30NP is supplied as a water wet solid with water content 45% w/w. All NMR data were in agreement with the literature ^b^ Conversions calculated by ^1^H NMR ^c^ unreduced dry ESCAT 1371 Engelhard ^d^ reduced, dry ESCAT 1241 Engelhard ^e^ 10% Pd/C Engelhard C3645 Aldrich ref. 520888, 3% of aldehyde is observed ^f^ 5% Pd/C Aldrich ref. 205680 ^g^ 5% Pd/C Johnson Matthey ref. 113026 ^h^ see Pears, D. A.; Smith, S. C. *Aldrichimica Acta* **2005,** *38,* 24–33.

In the case of 4-methoxyacetophenone the same excellent selectivity was achieved and the alcohol was obtained in 100% conversion when using Pd(0)EnCat™ 30NP under our standard conditions (entry 9). It is worthwhile mentioning that, as we expected, transfer hydrogenation conditions did not work so efficiently for this electron rich aromatic system. [17–18]

To explore the scope of this reaction, different electron rich and electron deficient aldehydes were also tested under these standard conditions using Pd(0)EnCat™ 30NP as a catalyst. In general good conversions to the corresponding benzylic alcohol were obtained. The results are summarized in Table 2.

**Table 2:** Pd(0)EnCat™30NP-catalyzed hydrogenation of aryl ketones and aldehydes.

**Entry**	**Substrate^a^** **Product**	**Conversion^b^**

1	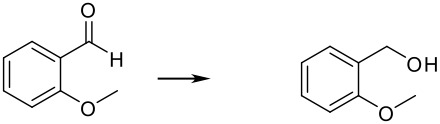	100%
2	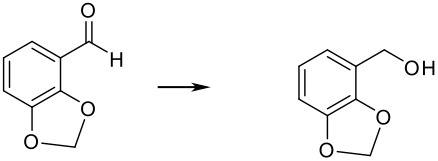	100%
3	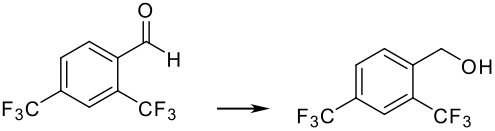	100%
4	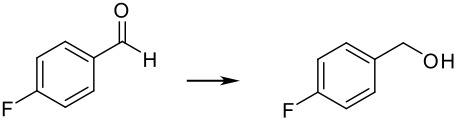	87%

^a^ Reaction conditions: H_2_ Balloon, 10 mol% Pd(0)EnCat™ 30NP, r.t., 16 hours. Pd(0)EnCat™ 30NP is supplied as a water wet solid with water content 45% w/w. All NMR data were in agreement with the literature ^b^ Conversions calculated by ^1^H NMR ^c^ 13% 4-fluorobenzoic acid

*trans*-Stilbene oxide was chosen as a model for the hydrogenolysis of aromatic epoxides. The effect of different heterogeneous palladium catalysts and their propensity to over-reduce the benzylic alcohol generated after hydrogenolysis of the epoxide was investigated. In this case similar encouraging results were obtained (see Table 3). Employing Pd/C resulted in complete conversion to the over-reduced product (entry 3), however, the use of Pd(0)EnCat 30NP as supplied gives the benzylic alcohol in 93% conversion (entry 1) which is improved to a conversion of 96% when the catalyst is pre-washed with ethanol (Table 3, entry 2). In this case, transfer hydrogenation conditions using ammonium formate as a source of hydrogen works equally well (entry 4 in Table 3).

**Table 3:** Pd(0)EnCat™ 30NP-Catalyzed Hydrogenolysis of trans-stilbene oxide. Comparison with other Pd catalysts.

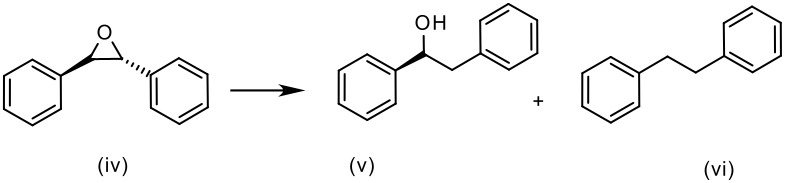				
**Entry**	**Method^a^**	**Solvent**	**Conversion^b^**	
			**(v)%**	**(vi)%**

1	H_2_, Pd(0)EnCat™ 30NP	EtOH	93	7
2	H_2_, Pd(0)EnCat™ 30NP pre-washed with EtOH	EtOH	96	4
3	H_2_, 5% Pd/C Aldrich^c^	EtOH	---	100
4	HCO_2_NH_4_, Pd(0)EnCat™ 30NP	MeOH/H_2_O	98	2

^a^ Reaction conditions: H_2_ Balloon or HCO_2_NH_4_, 10 mol% catalyst, r.t., 16 hours. Pd(0)EnCat™ 30NP is supplied as a water wet solid with water content 45% w/w. All NMR data were in agreement with the literature ^b^ Conversions calculated by ^1^H NMR ^c^ 5% Pd/C Aldrich ref. 205680.

Finally, different benzylic epoxides were also subjected to the same standard conditions established above and good conversions of the homobenzylic alcohol were consistently obtained (Table 4)

**Table 4:** Pd(0)EnCat™30NP-catalyzed hydrogenolysis of aryl epoxides.

**Entry**	**Substrate^a^** ** Product**	**Conversion^b^**

1	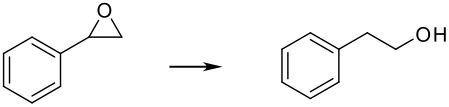	100%
2	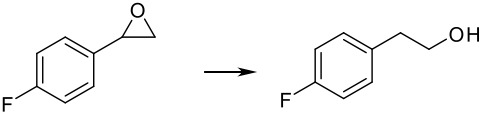	100%
3	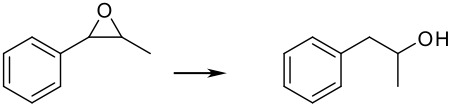	100%
4	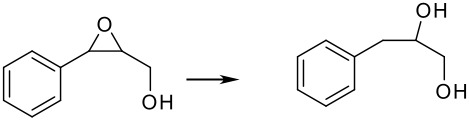	100%

^a^ Reaction conditions: H_2_ Balloon, 10 mol% Pd(0)EnCat™ 30NP, r.t., 16 hours. Pd(0)EnCat™ 30NP is supplied as a water wet solid with water content 45% w/w. All NMR data were in agreement with the literature ^b^ Conversions calculated by ^1^H NMR

In a typical procedure all reactions were performed on a 1 mmol scale. The substrate was generally dissolved in 10 ml of EtOH, 10 mol% of the catalyst was added and the mixture degassed twice under vacuum (using a water pump) and refilling with hydrogen each time. The reaction mixture was left at room temperature overnight connected to a double layer balloon of hydrogen. The catalyst was then filtered off, washed with EtOH, and the filtrate concentrated to give a crude product which was submitted for ^1^H-NMR analysis to determine the conversion. "Pre-washing" of the catalyst was as follows: The amount of wet catalyst required was weighed and then washed with the solvent in which the reaction took place.

## Conclusion

In conclusion, we have found that Pd(0)EnCat™ 30NP as a catalyst during hydrogenation reactions can selectively reduce aromatic aldehydes and ketones as well as aromatic epoxides to the corresponding alcohols in high conversions under mild conditions. We have also shown that Pd(0)EnCat™ 30NP gives better selectivities compared to Pd/C and other supported palladium catalysts such as Pd/CaCO_3_ or Pd/Al_2_O_3_.

## Supporting Information

File 1 R H Perni Supp Inf 170806.PDF contains all experimental details.
