# Supplementary material for: Hydrogenation of aromatic ketones, aldehydes, and epoxides with hydrogen and Pd(0)EnCat™ 30NP
Source: Beilstein J Org Chem. 2006 Aug 25;2:15. doi: 10.1186/1860-5397-2-15 (PMC1630636; doi:10.1186/1860-5397-2-15)
Supplement: File 1 — R H Perni Supp Inf 170806.PDF contains all experimental details. [file Beilstein_J_Org_Chem-02-15-s001.pdf]

Supplementary data as an aid to the referees

Hydrogenation of aromatic ketones, aldehydes, and epoxides with hydrogen and Pd(0)EnCat™ 30NP.

Steven V. Ley [a], Angus J. P. Stewart-Liddon[b], David Pears [b], Remedios H. Perni [b],* Kevin Treacher [b]

a- Department of Chemistry, University of Cambridge, Lensfield Road, Cambridge (UK) CB2 1EW

b- Reaxa Ltd, Hexagon Tower, Blackley, Manchester (UK) M9 8ZS

Email: Steven V. Ley – svl1000@cam.ac.uk; Angus J. P. Stewart-Liddon – angus.liddon@reaxa.co.uk; David Pears – david.pears@reaxa.co.uk; Remedios H. Perni – remediosperni@reaxa.co.uk; Kevin Treacher – kevin.treacher@reaxa.co.uk

* Corresponding author

General: The compounds described in this paper were prepared according to the procedure discussed in the text by the authors. All starting materials were purchased from Sigma-Aldrich and used without further purification. All reaction products were identified by comparison with their ^{1}H NMR spectra versus authentic ^{1}H NMR obtained from Sigma-Aldrich database and/or the authentic sample. Conversions were calculated from the ^{1}H NMR spectrum based on the disappearance of the starting material. ^{1}H NMR spectra were recorded on a Bruker DPX300 300 MHz NMR spectrometer at room temperature. Chemical shifts are reported in ppm on the δ scale relative to the internal standard TMS.

Table (1)

4-Methoxybenzyl alcohol: ^{1}H NMR ($CDCl_3$): δ 7.28 (2H, m, Ar), 6.89 (2H, m, Ar), 4.61 (2H, s, CH_2), 3.81 (3H, s, CH_3), 1.71 (1H, broad s, OH). In agreement with Sigma Aldrich database http://www.sigmaaldrich.com/catalog/search/ProductDetail/ALDRICH/136905

4-Methylanisole: ^{1}H NMR ($CDCl_3$): δ 7.01 (2H, m, Ar), 6.80 (2H, m, Ar), 3.78 (3H, s, CH_3), 2.27 (3H, s, CH_3). In agreement with Sigma Aldrich database http://www.sigmaaldrich.com/catalog/search/ProductDetail/FLUKA/61050

4-Methoxy-alpha-methylbenzyl alcohol: ^{1}H NMR ($CDCl_3$): δ 7.30 (2H, m, Ar), 6.88 (2H, m, Ar), 4.86 (1H, q, J = 6.44 Hz, CH), 3.81 (3H, s, CH_3), 1.70 (1H, broad s, OH), 1.48 (3H, d, J = 6.44 Hz, CH_3). In agreement with commercially available sample.

Table (2)

2-Methoxy benzylalcohol: ^{1}H NMR ($CDCl_3$) δ 7.29-7.23 (2H, m, Ar), 6.96-6.86 (2H, m, Ar), 4.67 (2H, s, CH_2), 3.84 (3H, s, CH_3), 2.47 (1H, s, OH) in agreement with the Sigma Aldrich database. http://www.sigmaaldrich.com/catalog/search/ProductDetail/ALDRICH/M10808

2,3-Methylenedioxy benzylalcohol: ^{1}H NMR ($CDCl_3$) δ 6.86-6.75 (3H, m, Ar), 5.95 (2H, s, CH_2), 4.66 (2H, s, CH_2), 2.16(1H, bs, OH) in agreement with data published by Roesch KR, Larock RC, *J Org Chem* 2002, **67**: 86.

2,4-Bistrifluoromethyl benzylalcohol: ^{1}H NMR ($CDCl_3$) δ 7.96-7.84 (3H, m, Ar), 4.97 (2H, s, CH_2), 2.11 (1H, bs, 2H).

4-Fluorobenzaldehyde: ^{1}H NMR ($CDCl_3$) δ 7.27 (2H, m), 7.00 (2H,m), 4.58 (2H, s), 3.88 (1H, bs, OH) in agreement with the Sigma Aldrich database.
http://www.sigmaaldrich.com/catalog/search/ProductDetail/FLUKA/46765
4-Fluorobenzoic acid: ^{1}H NMR ($CDCl_3$) δ 8.05 (2H, m), 7.09 (2H, m) in agreement with the Sigma Aldrich database. http://www.sigmaaldrich.com/catalog/search/ProductDetail/FLUKA/46600

Table (3)

2-phenyl-1-phenylethanol: ^{1}H NMR ($CDCl_3$): δ 7.40-7.15 (10H, m, Ar), 4.89 (1H, m,CH), 3.10-2.90 (2H, m, CH_2), 1.95 (1H, broad s OH) In agreement with data published by Blake AJ, Cunningham A, Ford A, Teat SJ, Woodward S, *Chem Eur J* 2000, **6**:3586.

Dibenzyl: ^{1}H NMR ($CDCl_3$): δ 7.35-7.25 (10H, m, Ar), 2.92 (4H, s, 2xCH_2). In agreement with Sigma Aldrich database.
http://www.sigmaaldrich.com/catalog/search/ProductDetail/FLUKA/14330

Table (4)

2-Phenylethanol: ^{1}H NMR ($CDCl_3$) δ 7.40-7.21 (5H, m, Ar), 3.85 (2H, t, J = 6.6 Hz, CH_2), 2.86 (2H, t, J = 6.6 Hz, CH_2), 1.55 (1H, bs, OH) in agreement with data published by Ley, SV, Mitchell C, Pears D, Ramaro C, Yu J-Q, Zhou W, *Org Lett*, 2003, **5**:4665.

2-(4'-Fluorophenyl) ethanol: ^{1}H NMR ($CDCl_3$) δ 7.21-7.16 (2H, m, Ar), 7.03-6.96 (2H, m, Ar), 3.84 (2H, t, J = 6.6 Hz, CH_2), 2.84 (2H, t, J = 6.6 Hz, CH_2), 1.49 (1H, bs, OH) in agreement data published by Ley, SV, Mitchell C, Pears D, Ramaro C, Yu J-Q, Zhou W, *Org Lett*, 2003, **5**:4665.

1-Phenyl propan-2-ol: ^{1}H NMR ($CDCl_3$) δ 7.32-7.12 (5H, m, Ar), 4.04-3.93 (1H, m, CH), 2.78-2.64 (2H, m, CH_2), 1.88 (1H, bs, OH), 1.21 (3H, d, J = 6.3 Hz, CH_3) in agreement data published by Ley, SV, Mitchell C, Pears D, Ramaro C, Yu J-Q, Zhou W, *Org Lett*, 2003, **5**:4665.

3-Phenyl propane-1,2-diol: ^{1}H NMR ($CDCl_3$) δ 7.37-7.20 (5H, m, Ar), 3.96-3.88 (m, 1H, C*H*OH), 3.66 (1H, dd, J = 11.1, 3.0 Hz, CH*H*), 3.49 (1H, dd, J = 11.1, 6.9 Hz, CH*H*), 2.82-2.69 (2H, m, CH_2), 2.47 (2H, bs, 2 × OH) in agreement data published by Ley, SV, Mitchell C, Pears D, Ramaro C, Yu J-Q, Zhou W, *Org Lett*, 2003, **5**:4665.

10375595, NBZ5781/4/1, BRUKER DPX300, QNP, H1 SPECTRUM, SOLV;- CDCl3/TMS

Intertek ASG

```
;zg30
;avance-version (02/05/31)
;1D sequence
;using 30 degree flip angle

#include <Avance.incl>

1 ze
2 30m
  d1
  p1*0.33 ph1
  go=2 ph31
  30m mc #0 to 2 F0(zd)
exit

ph1=0 2 2 0 1 3 3 1
ph31=0 2 2 0 1 3 3 1

;pl1 : f1 channel - power level for pulse (defaul
;p1 : f1 channel -  90 degree high power pulse
;d1 : relaxation delay; 1-5 * T1
```

TABLE 1 ENTRY 1

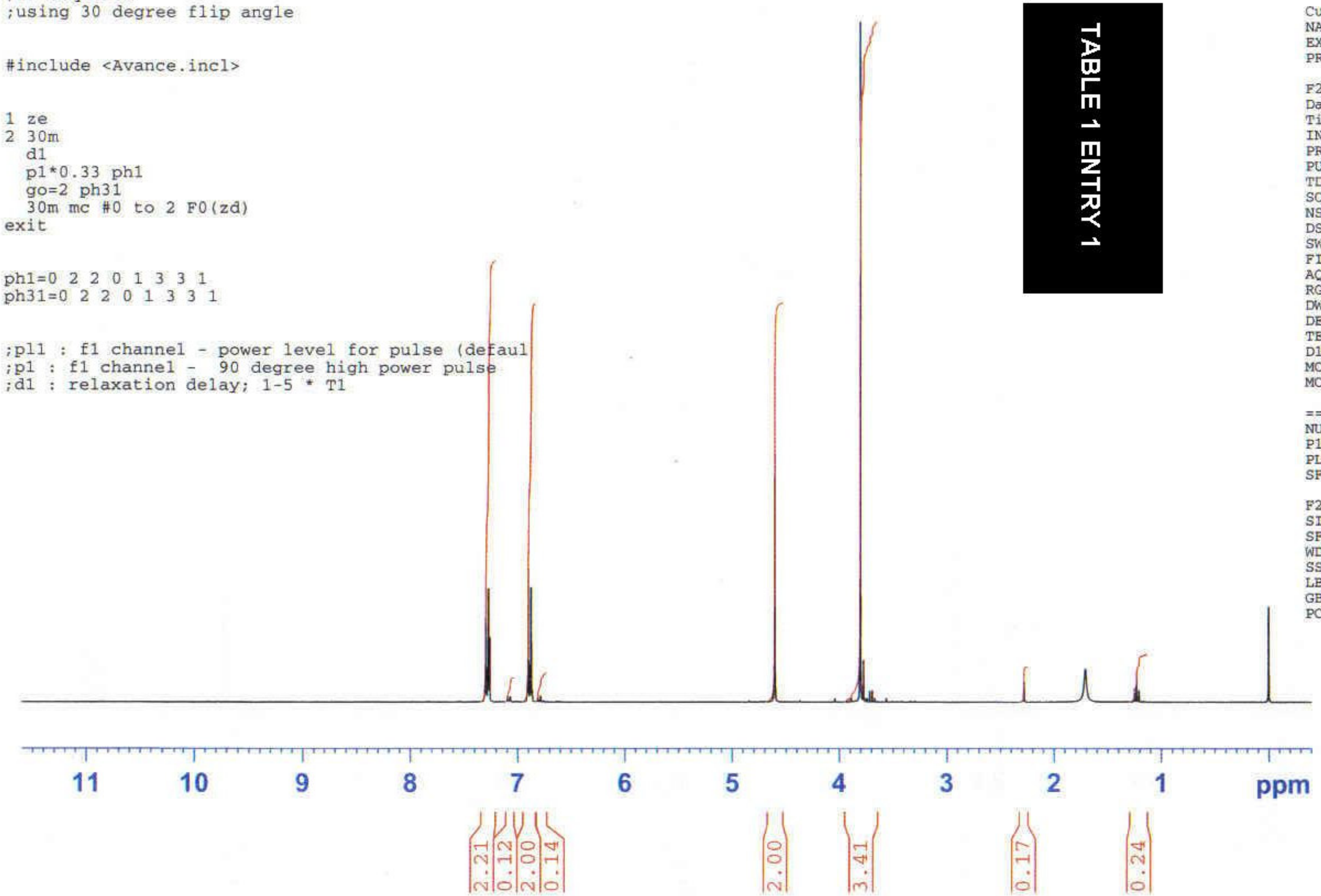


```
Current Data Parameters
NAME          10375595
EXPNO               10
PROCNO               1

F2 - Acquisition Parameters
Date_         20051111
Time             15.37
INSTRUM         dpx300
PROBHD   5 mm QNP 1H/1
PULPROG           zg30
TD               48848
SOLVENT          CDCl3
NS                  64
DS                   2
SWH           7183.908 Hz
FIDRES        0.147067 Hz
AQ           3.3998709 sec
RG               322.5
DW              69.600 usec
DE                6.00 usec
TE               299.2 K
D1          6.59999990 sec
MCREST      0.00000000 sec
MCWRK       0.01500000 sec

======== CHANNEL f1 ========
NUC1                1H
P1                9.40 usec
PL1               0.00 dB
SFO1       300.1330010 MHz

F2 - Processing parameters
SI               65536
SF         300.1300071 MHz
WDW                 EM
SSB                  0
LB                0.30 Hz
GB                   0
PC                2.00
```

S.M

11/11/05

10375595, NBZ5781/4/1, BRUKER DPX300, QNP, H1 SPECTRUM, SOLV;- CDC13/TMS

Intertek ASG

```
;zg30
;avance-version (02/05/31)
;1D sequence
;using 30 degree flip angle

#include <Avance.incl>

1 ze
2 30m
  d1
  p1*0.33 ph1
  go=2 ph31
  30m mc #0 to 2 F0(zd)
exit

ph1=0 2 2 0 1 3 3 1
ph31=0 2 2 0 1 3 3 1

;pl1 : f1 channel - power level for pulse (defaul
;p1 : f1 channel -  90 degree high power pulse
;d1 : relaxation delay; 1-5 * T1
```

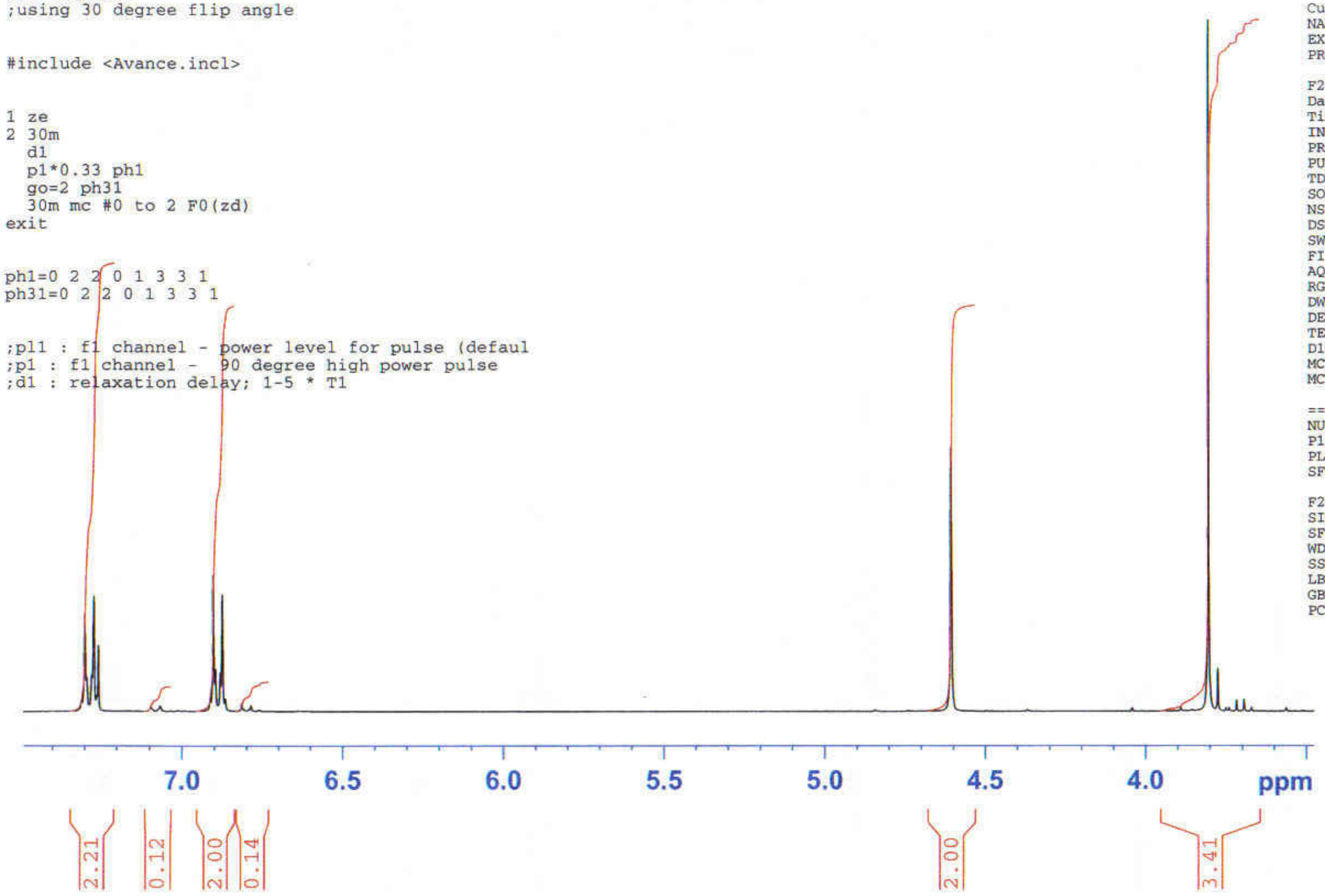


```
Current Data Parameters
NAME            10375595
EXPNO                 10
PROCNO                 1

F2 - Acquisition Parameters
Date_           20051111
Time               15.37
INSTRUM           dpx300
PROBHD   5 mm QNP  1H/1
PULPROG             zg30
TD                 48848
SOLVENT            CDC13
NS                    64
DS                     2
SWH             7183.908 Hz
FIDRES          0.147067 Hz
AQ             3.3998709 sec
RG                 322.5
DW                69.600 usec
DE                  6.00 usec
TE                 299.2 K
D1            6.59999990 sec
MCREST        0.00000000 sec
MCWRK         0.01500000 sec

======== CHANNEL f1 ========
NUC1                  1H
P1                  9.40 usec
PL1                 0.00 dB
SFO1         300.1330010 MHz

F2 - Processing parameters
SI                 65536
SF           300.1300071 MHz
WDW                   EM
SSB                    0
LB                  0.30 Hz
GB                     0
PC                  2.00
```

S.M

11/11/05

10376150, NBZ5781/10/4, BRUKER DPX300, QNP, H1 SPECTRUM, SOLV:-CDCL3/TMS

Intertek ASG

```
;zg30
;avance-version (02/05/31)
;1D sequence
;using 30 degree flip angle

#include <Avance.incl>

1 ze
2 30m
  d1
  p1*0.33 ph1
  go=2 ph31
  30m mc #0 to 2 F0(zd)
exit

ph1=0 2 2 0 1 3 3 1
ph31=0 2 2 0 1 3 3 1

;pl1 : f1 channel - power level for pulse (defaul
;p1 : f1 channel -  90 degree high power pulse
;d1 : relaxation delay; 1-5 * T1
```

TABLE 1 ENTRY 2

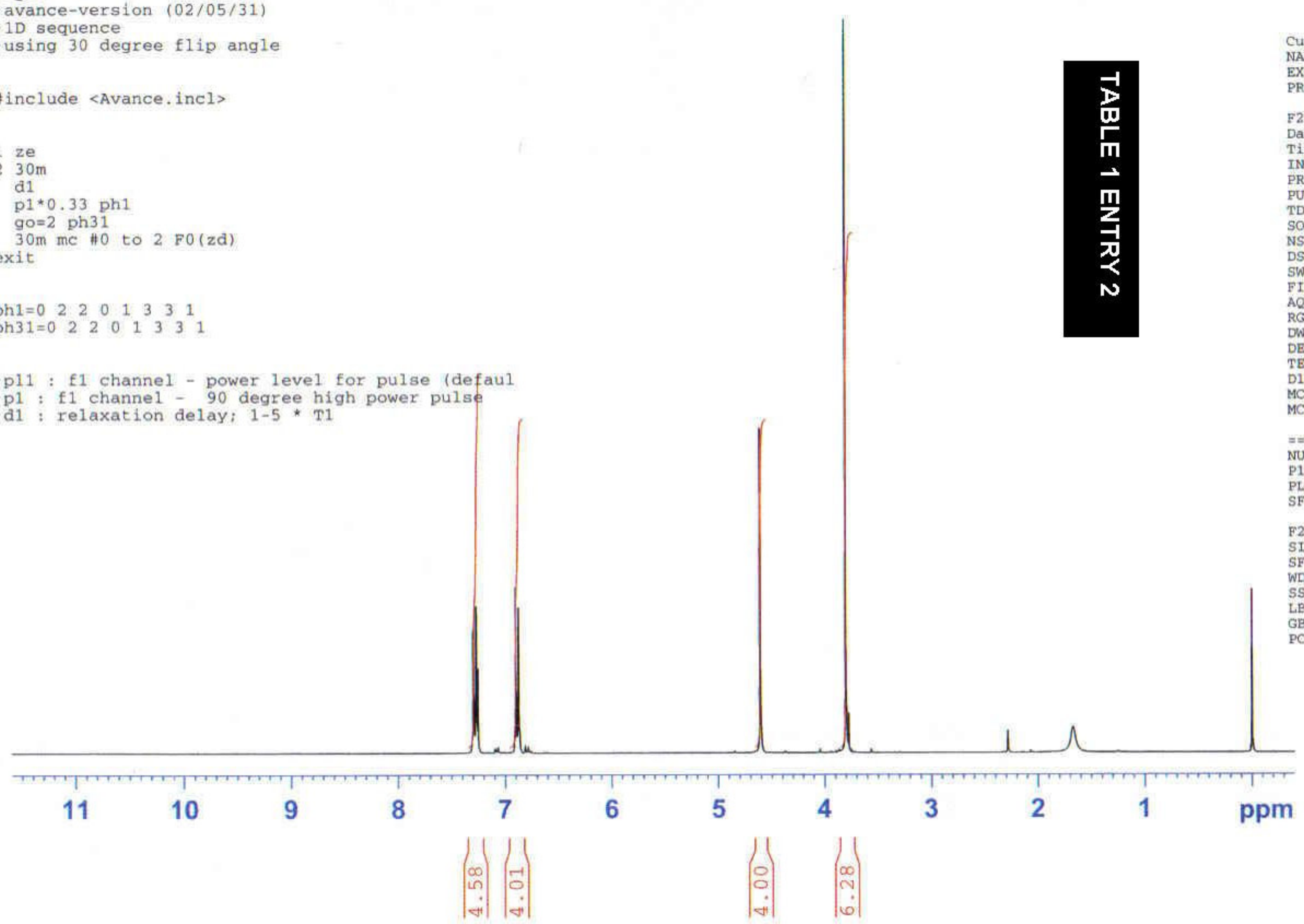


```
Current Data Parameters
NAME            10376150
EXPNO                 10
PROCNO                 1

F2 - Acquisition Parameters
Date_           20051125
Time               19.10
INSTRUM           dpx300
PROBHD    5 mm QNP  1H/1
PULPROG             zg30
TD                 48848
SOLVENT            CDCl3
NS                    64
DS                     2
SWH             7183.908 Hz
FIDRES          0.147067 Hz
AQ             3.3998709 sec
RG                 406.4
DW                69.600 usec
DE                  6.00 usec
TE                 299.2 K
D1            6.59999990 sec
MCREST        0.00000000 sec
MCWRK         0.01500000 sec

======== CHANNEL f1 ========
NUC1                  1H
P1                  9.40 usec
PL1                 0.00 dB
SFO1         300.1330010 MHz

F2 - Processing parameters
SI                 65536
SF           300.1300073 MHz
WDW                   EM
SSB                    0
LB                  0.30 Hz
GB                     0
PC                  2.00
```

10376150, NBZ5781/10/4, BRUKER DPX300, QNP, H1 SPECTRUM, SOLV:-CDCL3/TMS

Intertek ASG

```
Current Data Parameters
NAME              10376150
EXPNO                   10
PROCNO                   1

F2 - Acquisition Parameters
Date_             20051125
Time                 19.10
INSTRUM             dpx300
PROBHD     5 mm QNP  1H/1
PULPROG               zg30
TD                   48848
SOLVENT              CDCl3
NS                      64
DS                       2
SWH               7183.908 Hz
FIDRES            0.147067 Hz
AQ               3.3998709 sec
RG                   406.4
DW                  69.600 usec
DE                    6.00 usec
TE                   299.2 K
D1              6.59999990 sec
MCREST          0.00000000 sec
MCWRK           0.01500000 sec

======== CHANNEL f1 ========
NUC1                    1H
P1                    9.40 usec
PL1                   0.00 dB
SFO1           300.1330010 MHz

F2 - Processing parameters
SI                   65536
SF             300.1300073 MHz
WDW                     EM
SSB                      0
LB                    0.30 Hz
GB                       0
PC                    2.00
```

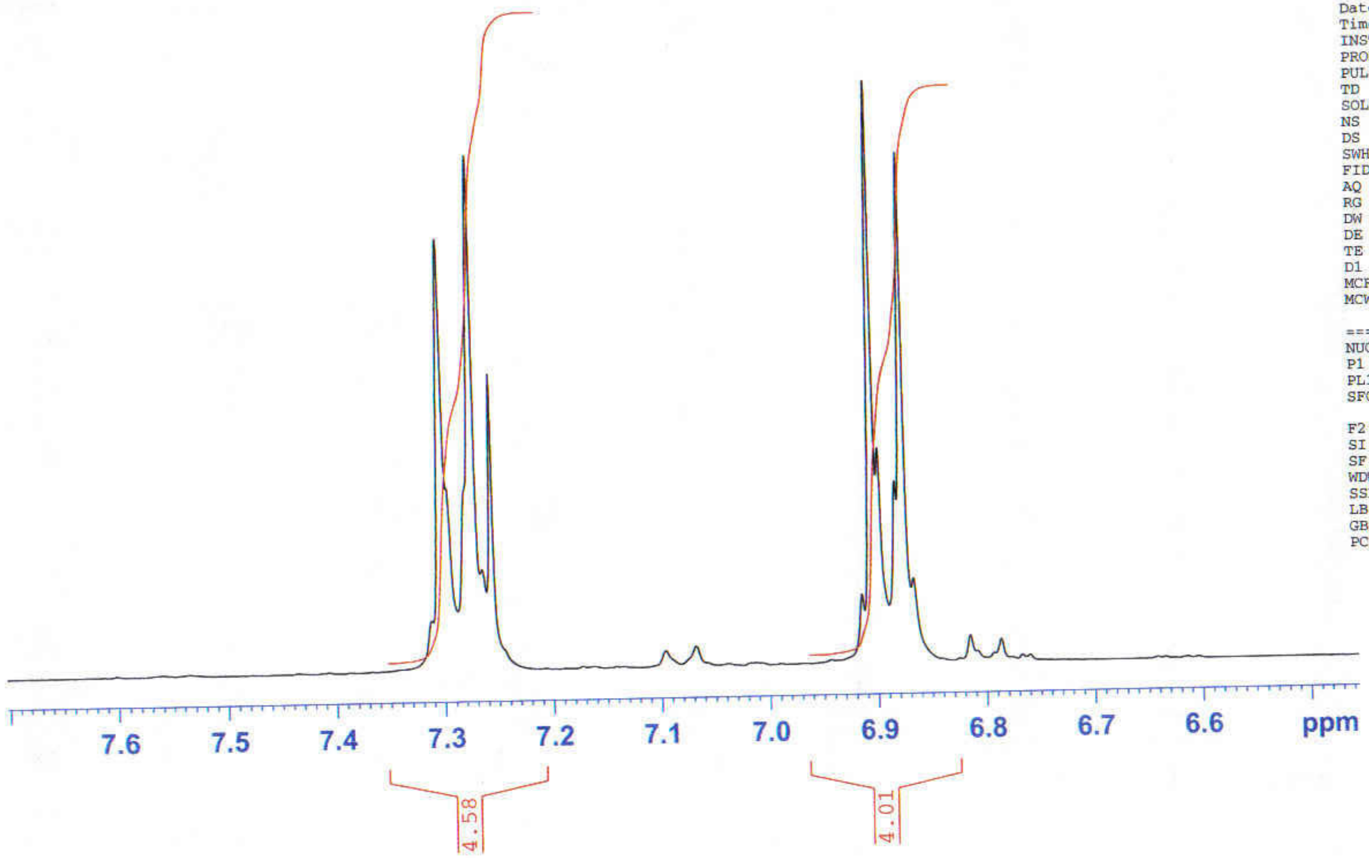

10375597, NBZ5781/4/2, BRUKER DPX300, QNP, H1 SPECTRUM, SOLV;- CDC13/TMS

Intertek ASG

```
;zg30
;avance-version (02/05/31)
;1D sequence
;using 30 degree flip angle

#include <Avance.incl>

1 ze
2 30m
  d1
  p1*0.33 ph1
  go=2 ph31
  30m mc #0 to 2 F0(zd)
exit

ph1=0 2 2 0 1 3 3 1
ph31=0 2 2 0 1 3 3 1

;pl1 : f1 channel - power level for pulse (defaul
;p1 : f1 channel -  90 degree high power pulse
;d1 : relaxation delay; 1-5 * T1
```

TABLE 1 ENTRY 3

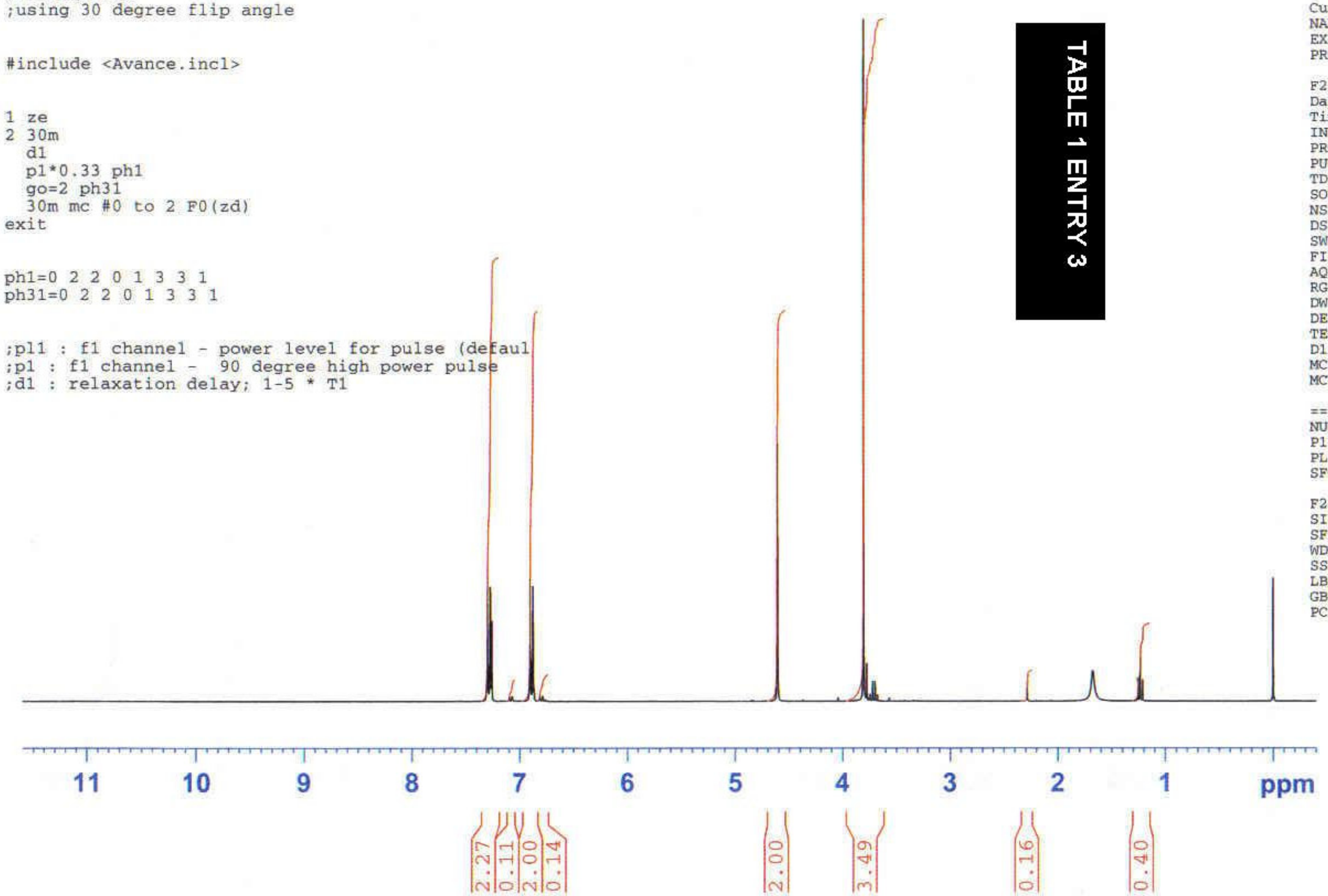


```
Current Data Parameters
NAME              10375597
EXPNO                   10
PROCNO                   1

F2 - Acquisition Parameters
Date_             20051111
Time                 16.03
INSTRUM             dpx300
PROBHD     5 mm QNP  1H/1
PULPROG               zg30
TD                   48848
SOLVENT              CDC13
NS                      64
DS                       2
SWH               7183.908 Hz
FIDRES            0.147067 Hz
AQ               3.3998709 sec
RG                     362
DW                  69.600 usec
DE                    6.00 usec
TE                   299.2 K
D1              6.59999990 sec
MCREST          0.00000000 sec
MCWRK           0.01500000 sec

======== CHANNEL f1 ========
NUC1                    1H
P1                    9.40 usec
PL1                   0.00 dB
SFO1           300.1330010 MHz

F2 - Processing parameters
SI                   65536
SF             300.1300068 MHz
WDW                     EM
SSB                      0
LB                    0.30 Hz
GB                       0
PC                    2.00
```

S.M

11/11/05

10375597, NBZ5781/4/2, BRUKER DPX300, QNP, H1 SPECTRUM, SOLV;- CDCl3/TMS

Intertek ASG

```
;zg30
;avance-version (02/05/31)
;1D sequence
;using 30 degree flip angle

#include <Avance.incl>

1 ze
2 30m
  d1
  p1*0.33 ph1
  go=2 ph31
  30m mc #0 to 2 F0(zd)
exit

ph1=0 2 2 0 1 3 3 1
ph31=0 2 2 0 1 3 3 1

;pl1 : f1 channel - power level for pulse (defaul
;p1 : f1 channel -  90 degree high power pulse
;d1 : relaxation delay; 1-5 * T1
```

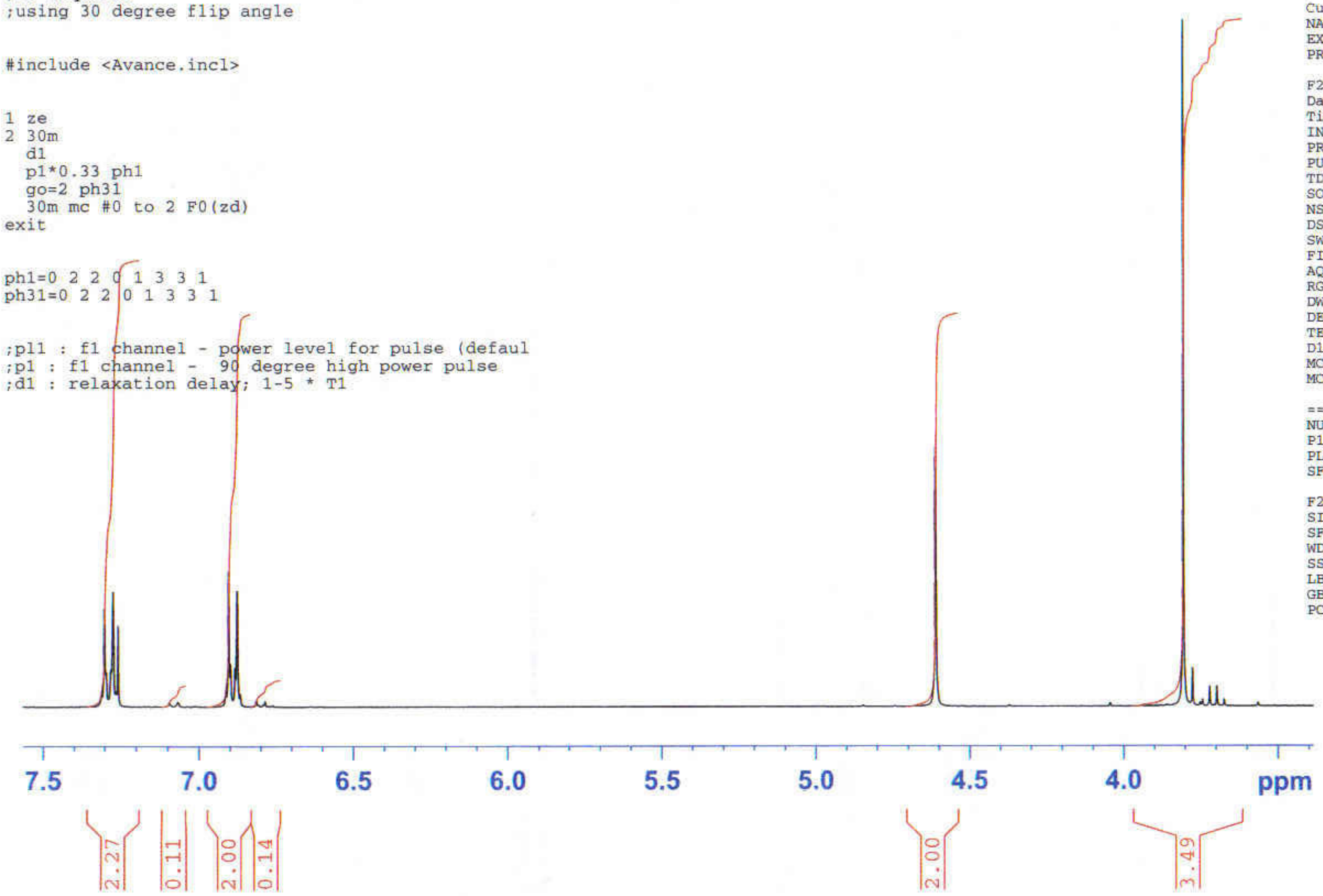


```
Current Data Parameters
NAME              10375597
EXPNO                   10
PROCNO                   1

F2 - Acquisition Parameters
Date_             20051111
Time                 16.03
INSTRUM             dpx300
PROBHD      5 mm QNP  1H/1
PULPROG               zg30
TD                   48848
SOLVENT              CDCl3
NS                      64
DS                       2
SWH               7183.908 Hz
FIDRES            0.147067 Hz
AQ               3.3998709 sec
RG                     362
DW                  69.600 usec
DE                    6.00 usec
TE                   299.2 K
D1              6.59999990 sec
MCREST          0.00000000 sec
MCWRK           0.01500000 sec

======== CHANNEL f1 ========
NUC1                    1H
P1                    9.40 usec
PL1                   0.00 dB
SFO1           300.1330010 MHz

F2 - Processing parameters
SI                   65536
SF             300.1300068 MHz
WDW                     EM
SSB                      0
LB                    0.30 Hz
GB                       0
PC                    2.00
```

S.M [illegible]
11|11|05

10376711, NBZ5781/14/1, BRUKER DPX300, QNP, H1 SPECTRUM, SOLV:-CDCL3/TMS

Intertek ASG

TABLE 1 ENTRY 4

```
;zg30
;avance-version (02/05/31)
;1D sequence
;using 30 degree flip angle

#include <Avance.incl>

1 ze
2 30m
  d1
  p1*0.33 ph1
  go=2 ph31
  30m mc #0 to 2 F0(zd)
exit

ph1=0 2 2 0 1 3 3 1
ph31=0 2 2 0 1 3 3 1

;pl1 : f1 channel - power level for pulse (defaul
;p1 : f1 channel -  90 degree high power pulse
;d1 : relaxation delay; 1-5 * T1
```

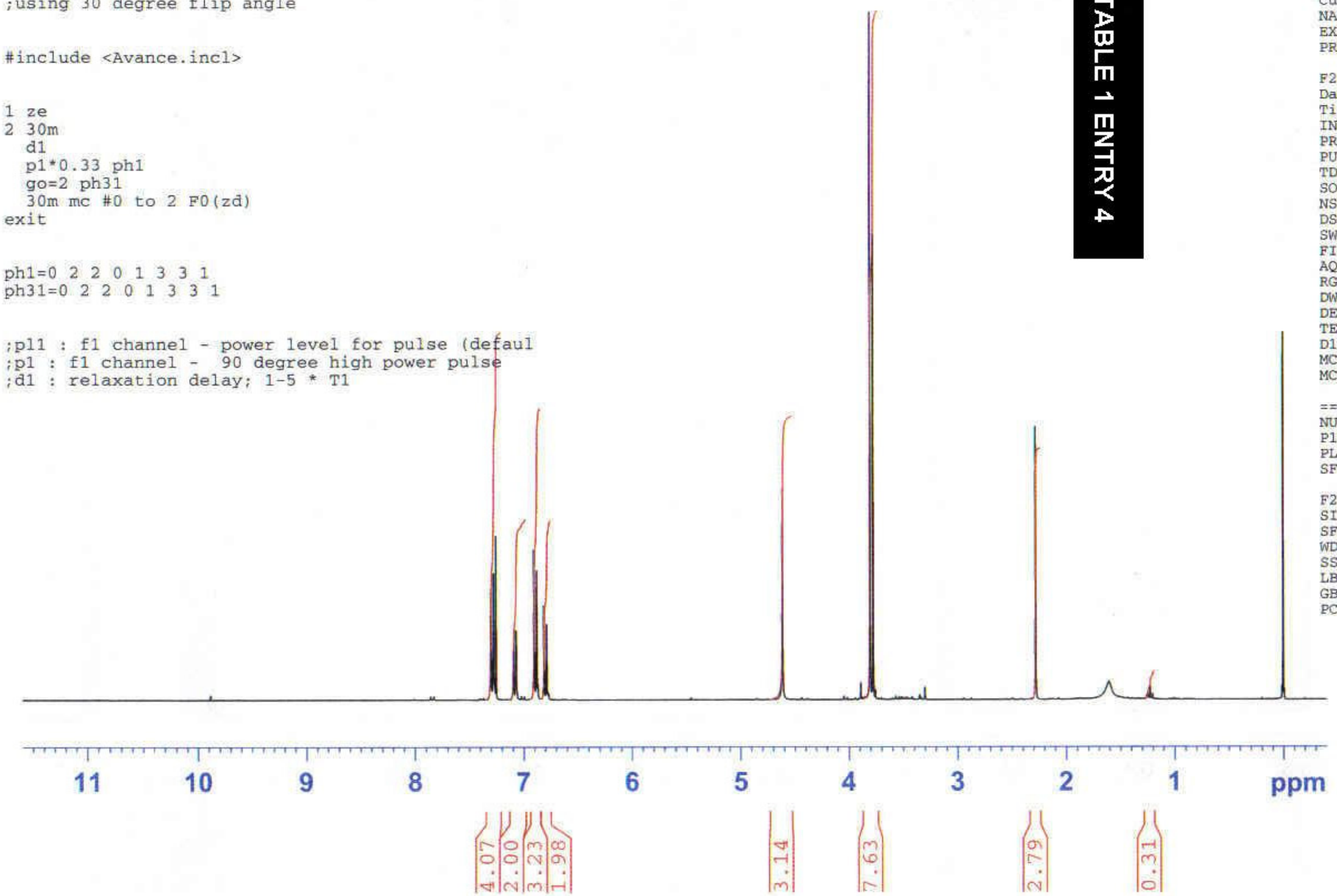


```
Current Data Parameters
NAME            10376711
EXPNO                 10
PROCNO                 1

F2 - Acquisition Parameters
Date_           20051207
Time               15.23
INSTRUM           dpx300
PROBHD   5 mm QNP   1H/1
PULPROG             zg30
TD                 48848
SOLVENT            CDCl3
NS                    64
DS                     2
SWH             7183.908 Hz
FIDRES          0.147067 Hz
AQ             3.3998709 sec
RG                   512
DW                69.600 usec
DE                  6.00 usec
TE                 299.2 K
D1            6.59999990 sec
MCREST        0.00000000 sec
MCWRK         0.01500000 sec

======== CHANNEL f1 ========
NUC1                  1H
P1                  9.40 usec
PL1                 0.00 dB
SFO1         300.1330010 MHz

F2 - Processing parameters
SI                 65536
SF           300.1300075 MHz
WDW                   EM
SSB                    0
LB                  0.30 Hz
GB                     0
PC                  2.00
```

S.n

7/12/05

10376711, NBZ5781/14/1, BRUKER DPX300, QNP, H1 SPECTRUM, SOLV:-CDCL3/TMS

Intertek ASG

```
;zg30
;avance-version (02/05/31)
;1D sequence
;using 30 degree flip angle

#include <Avance.incl>

1 ze
2 30m
  d1
  p1*0.33 ph1
  go=2 ph31
  30m mc #0 to 2 F0(zd)
exit

ph1=0 2 2 0 1 3 3 1
ph31=0 2 2 0 1 3 3 1

;pl1 : f1 channel - power level for pulse (defaul
;p1 : f1 channel -  90 degree high power pulse
;d1 : relaxation delay; 1-5 * T1
```

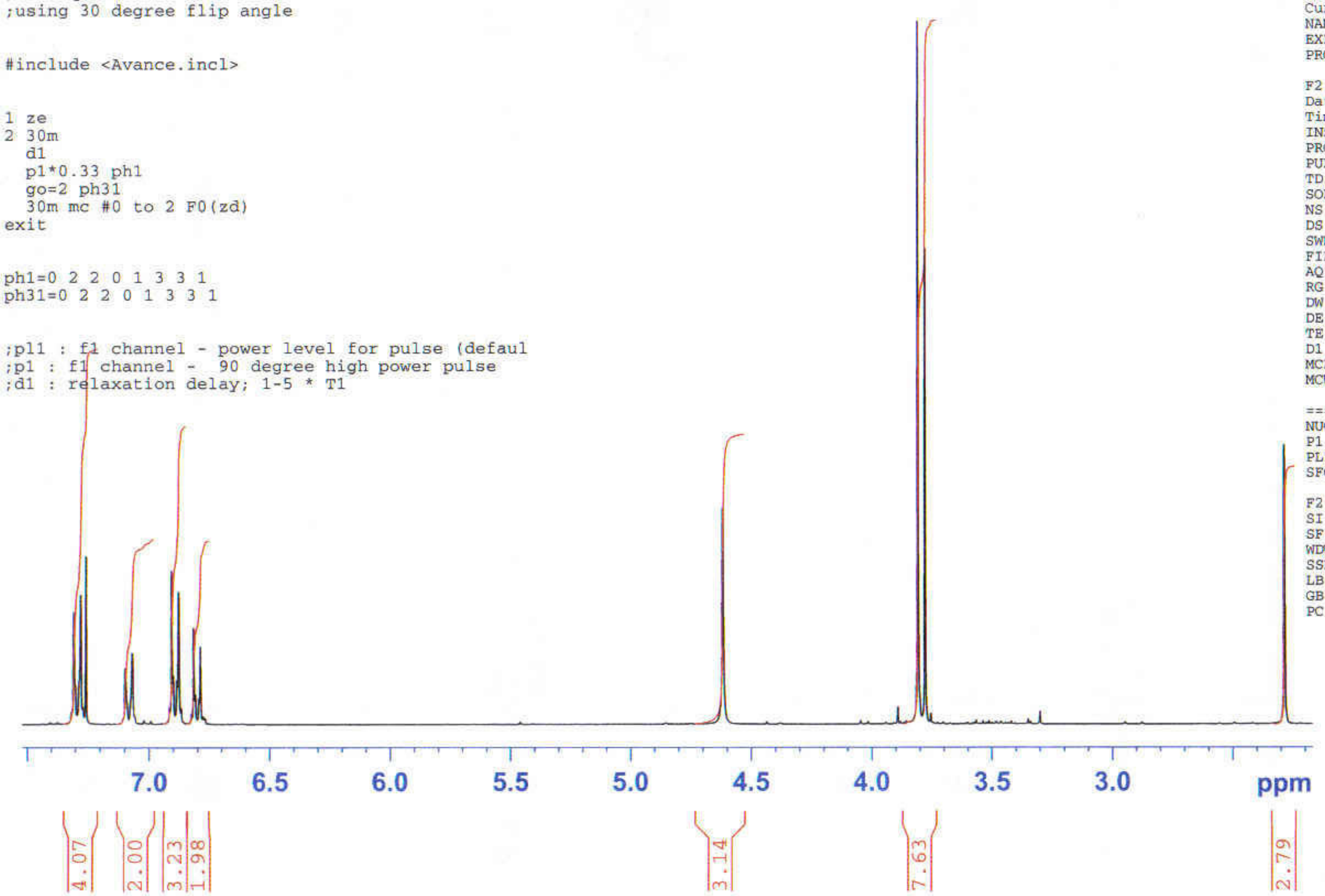


```
Current Data Parameters
NAME          10376711
EXPNO               10
PROCNO               1

F2 - Acquisition Parameters
Date_         20051207
Time             15.23
INSTRUM         dpx300
PROBHD   5 mm QNP  1H/1
PULPROG           zg30
TD               48848
SOLVENT          CDCl3
NS                  64
DS                   2
SWH           7183.908 Hz
FIDRES        0.147067 Hz
AQ           3.3998709 sec
RG                 512
DW              69.600 usec
DE                6.00 usec
TE               299.2 K
D1          6.59999990 sec
MCREST      0.00000000 sec
MCWRK       0.01500000 sec

======== CHANNEL f1 ========
NUC1                1H
P1                9.40 usec
PL1               0.00 dB
SFO1       300.1330010 MHz

F2 - Processing parameters
SI               65536
SF         300.1300075 MHz
WDW                 EM
SSB                  0
LB                0.30 Hz
GB                   0
PC                2.00
```

S.A 7/12/05

10376147, NBZ5781/10/1, BRUKER DPX300, QNP, H1 SPECTRUM, SOLV:-CDCL3/TMS

Intertek ASG

```
;zg30
;avance-version (02/05/31)
;1D sequence
;using 30 degree flip angle

#include <Avance.incl>

1 ze
2 30m
  d1
  p1*0.33 ph1
  go=2 ph31
  30m mc #0 to 2 F0(zd)
exit

ph1=0 2 2 0 1 3 3 1
ph31=0 2 2 0 1 3 3 1

;pl1 : f1 channel - power level for pulse (defaul
;p1 : f1 channel -  90 degree high power pulse
;d1 : relaxation delay; 1-5 * T1
```

TABLE 1 ENTRY 5

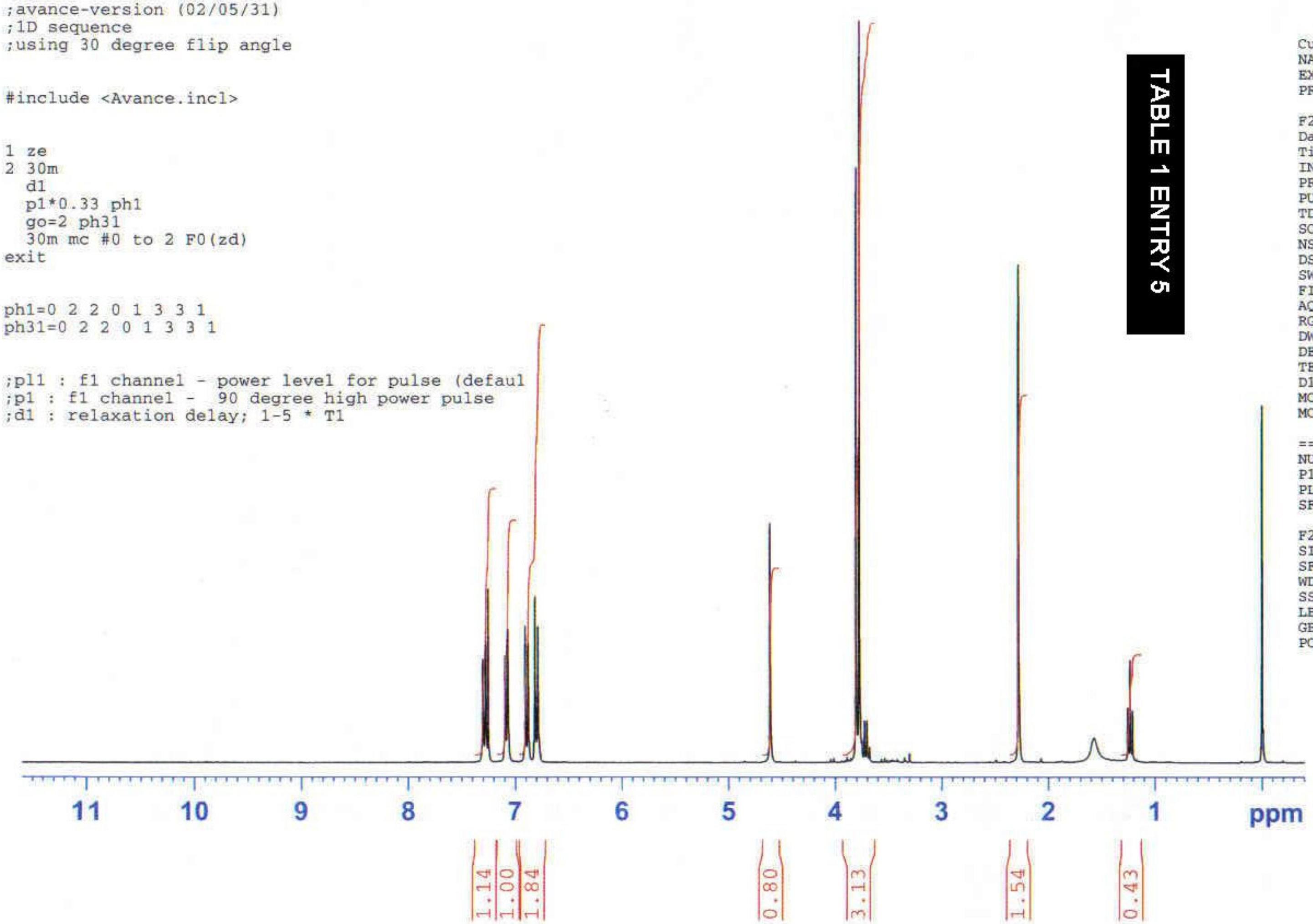


```
Current Data Parameters
NAME            10376147
EXPNO                 10
PROCNO                 1

F2 - Acquisition Parameters
Date_           20051125
Time               18.17
INSTRUM           dpx300
PROBHD    5 mm QNP  1H/1
PULPROG             zg30
TD                 48848
SOLVENT            CDCl3
NS                    64
DS                     2
SWH             7183.908 Hz
FIDRES          0.147067 Hz
AQ             3.3998709 sec
RG                 456.1
DW                69.600 usec
DE                  6.00 usec
TE                 299.2 K
D1            6.59999990 sec
MCREST        0.00000000 sec
MCWRK         0.01500000 sec

======== CHANNEL f1 ========
NUC1                  1H
P1                  9.40 usec
PL1                 0.00 dB
SFO1         300.1330010 MHz

F2 - Processing parameters
SI                 65536
SF           300.1300077 MHz
WDW                   EM
SSB                    0
LB                  0.30 Hz
GB                     0
PC                  2.00
```

D. Muir 28/11/05

10376147, NBZ5781/10/1, BRUKER DPX300, QNP, H1 SPECTRUM, SOLV:-CDCL3/TMS

Intertek ASG

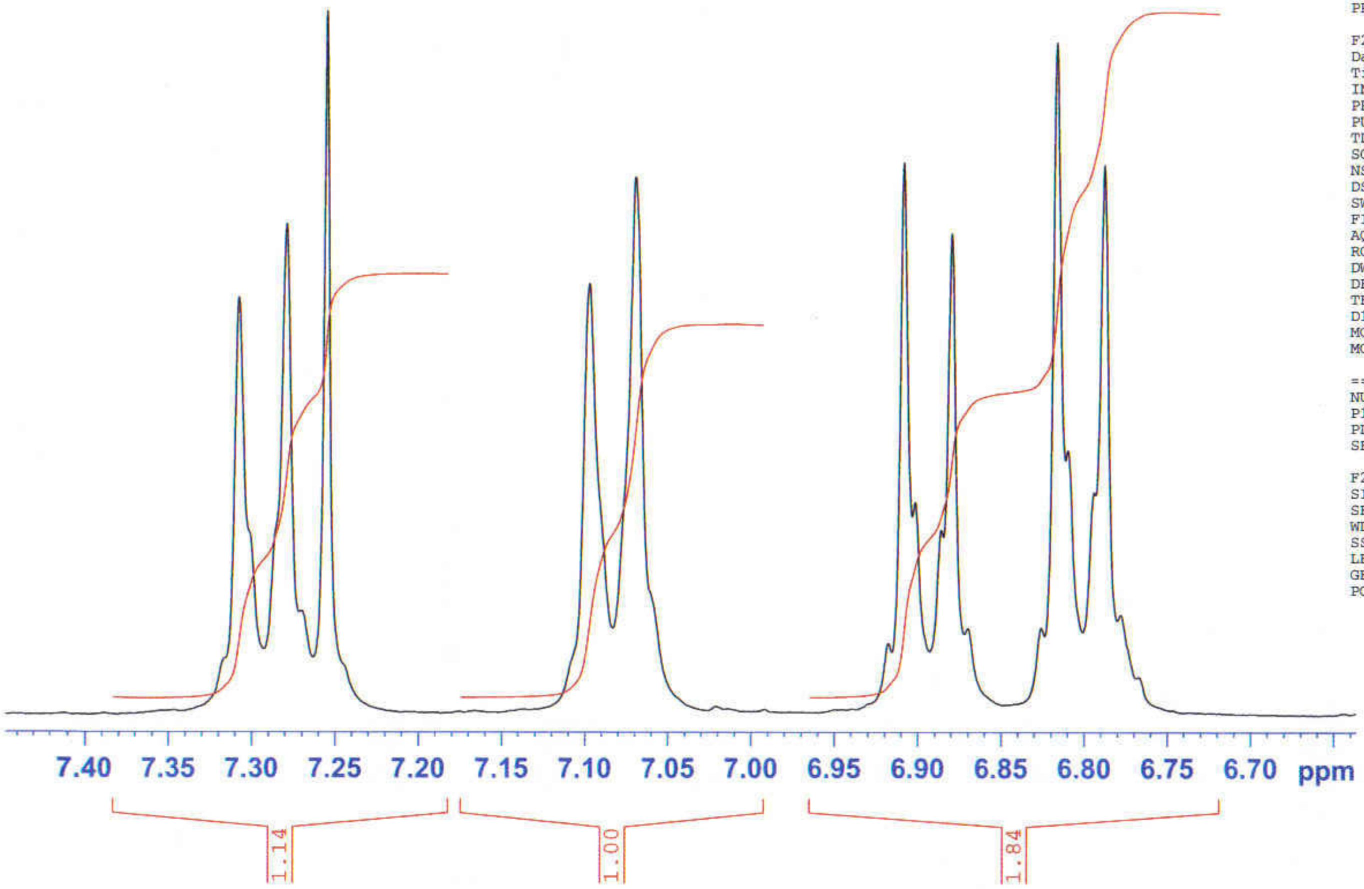


```
Current Data Parameters
NAME           10376147
EXPNO                10
PROCNO                1

F2 - Acquisition Parameters
Date_          20051125
Time              18.17
INSTRUM          dpx300
PROBHD   5 mm QNP  1H/1
PULPROG            zg30
TD                48848
SOLVENT           CDCl3
NS                   64
DS                    2
SWH            7183.908 Hz
FIDRES         0.147067 Hz
AQ            3.3998709 sec
RG                456.1
DW               69.600 usec
DE                 6.00 usec
TE                299.2 K
D1           6.59999990 sec
MCREST       0.00000000 sec
MCWRK        0.01500000 sec

======== CHANNEL f1 ========
NUC1                 1H
P1                 9.40 usec
PL1                0.00 dB
SFO1        300.1330010 MHz

F2 - Processing parameters
SI                65536
SF          300.1300077 MHz
WDW                  EM
SSB                   0
LB                 0.30 Hz
GB                    0
PC                 2.00
```

28/11/05

10376147, NBZ5781/10/1, BRUKER DPX300, QNP, H1 SPECTRUM, SOLV:-CDCL3/TMS

Intertek ASG

```
Current Data Parameters
NAME              10376147
EXPNO                   10
PROCNO                   1

F2 - Acquisition Parameters
Date_             20051125
Time                 18.17
INSTRUM             dpx300
PROBHD    5 mm QNP  1H/1
PULPROG               zg30
TD                   48848
SOLVENT              CDCl3
NS                      64
DS                       2
SWH               7183.908 Hz
FIDRES            0.147067 Hz
AQ               3.3998709 sec
RG                   456.1
DW                  69.600 usec
DE                    6.00 usec
TE                   299.2 K
D1              6.59999990 sec
MCREST          0.00000000 sec
MCWRK           0.01500000 sec

======== CHANNEL f1 ========
NUC1                    1H
P1                    9.40 usec
PL1                   0.00 dB
SFO1           300.1330010 MHz

F2 - Processing parameters
SI                   65536
SF             300.1300077 MHz
WDW                     EM
SSB                      0
LB                    0.30 Hz
GB                       0
PC                    2.00
```

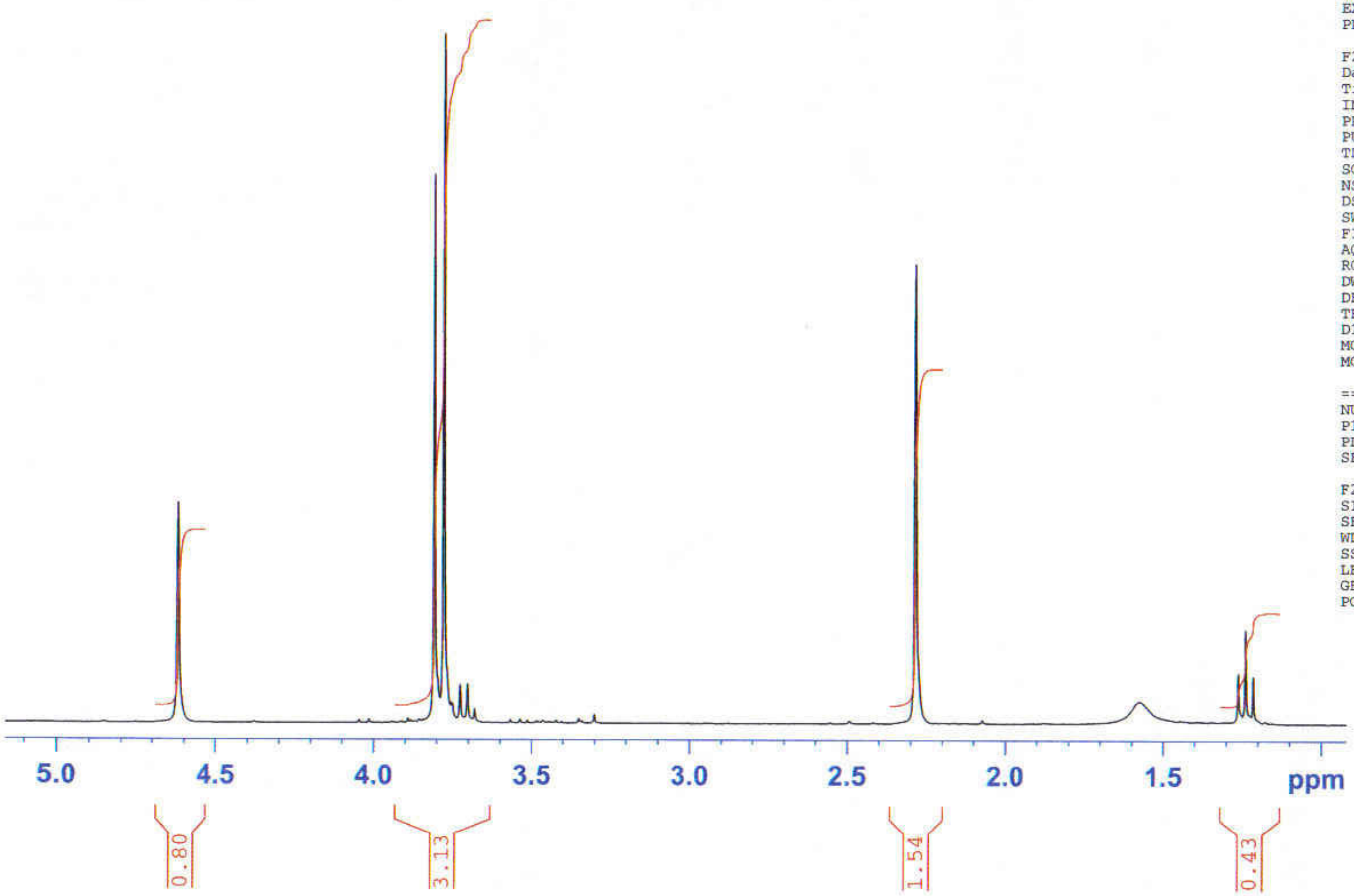

10371786, NBZ5769/42/A, BRUKER DPX300, QNP. H1 SPECTRUM, SOLV:-CDCL3/TMS

Intertek ASG

```
;zg30
;avance-version (02/05/31)
;1D sequence
;using 30 degree flip angle

#include <Avance.incl>

1 ze
2 30m
  d1
  p1*0.33 ph1
  go=2 ph31
  30m mc #0 to 2 F0(zd)
exit

ph1=0 2 2 0 1 3 3 1
ph31=0 2 2 0 1 3 3 1

;pl1 : f1 channel - power level for pulse (defaul
;p1 : f1 channel -  90 degree high power pulse
;d1 : relaxation delay; 1-5 * T1
```

TABLE 1 ENTRY 6

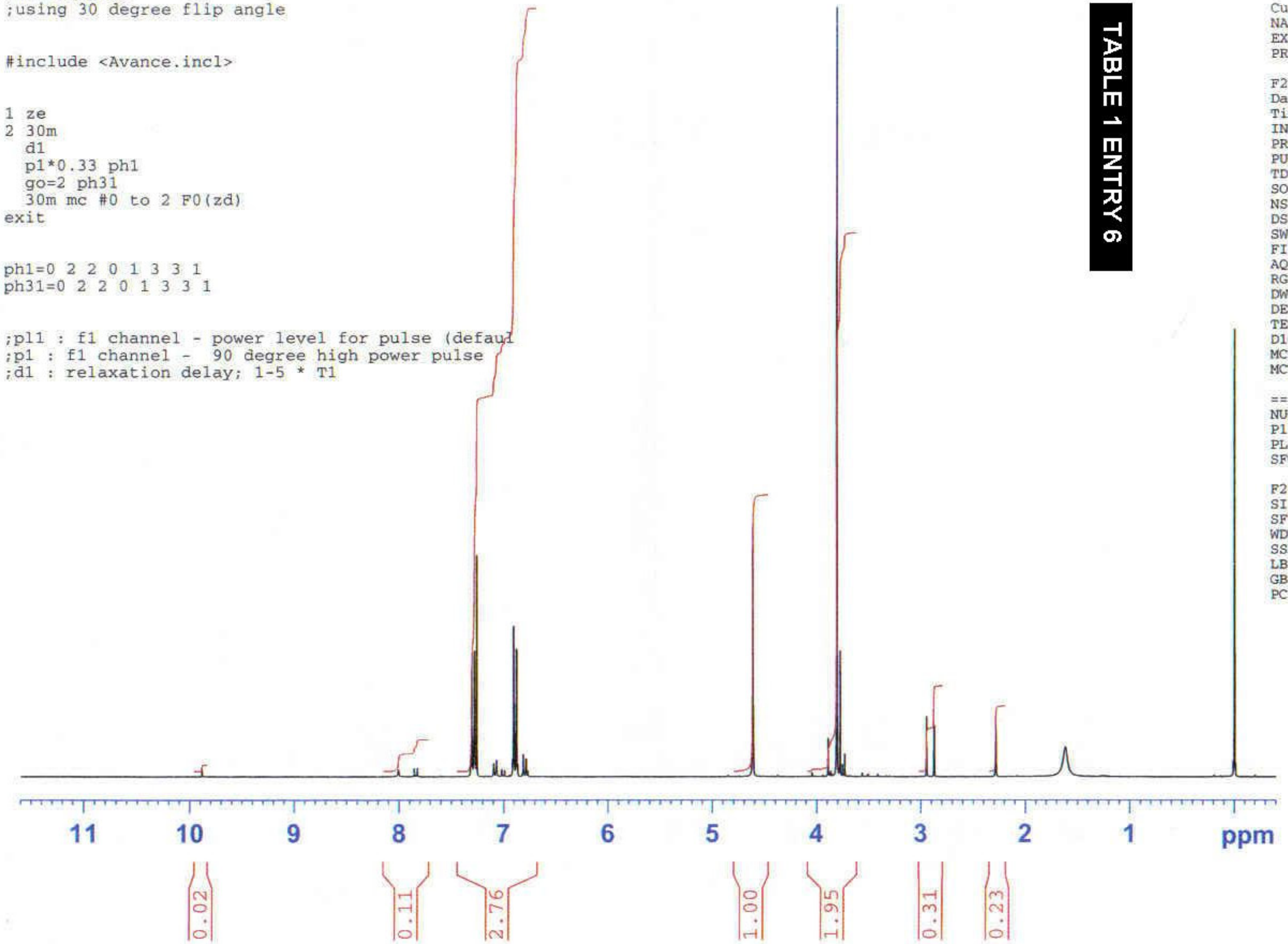


```
Current Data Parameters
NAME              10371786
EXPNO                   10
PROCNO                   1

F2 - Acquisition Parameters
Date_             20050819
Time                 18.34
INSTRUM             dpx300
PROBHD     5 mm QNP  1H/1
PULPROG               zg30
TD                   48848
SOLVENT              CDCl3
NS                      64
DS                       2
SWH               7183.908 Hz
FIDRES            0.147067 Hz
AQ               3.3998709 sec
RG                   645.1
DW                  69.600 usec
DE                    6.00 usec
TE                   299.2 K
D1              6.59999990 sec
MCREST          0.00000000 sec
MCWRK           0.01500000 sec

======== CHANNEL f1 ========
NUC1                    1H
P1                    9.40 usec
PL1                   0.00 dB
SFO1           300.1330010 MHz

F2 - Processing parameters
SI                   65536
SF             300.1300064 MHz
WDW                     EM
SSB                      0
LB                    0.30 Hz
GB                       0
PC                    2.00
```

D. Mutt 22/8/05

10371786, NBZ5769/42/A, BRUKER DPX300, QNP. H1 SPECTRUM, SOLV:-CDCL3/TMS

Intertek ASG

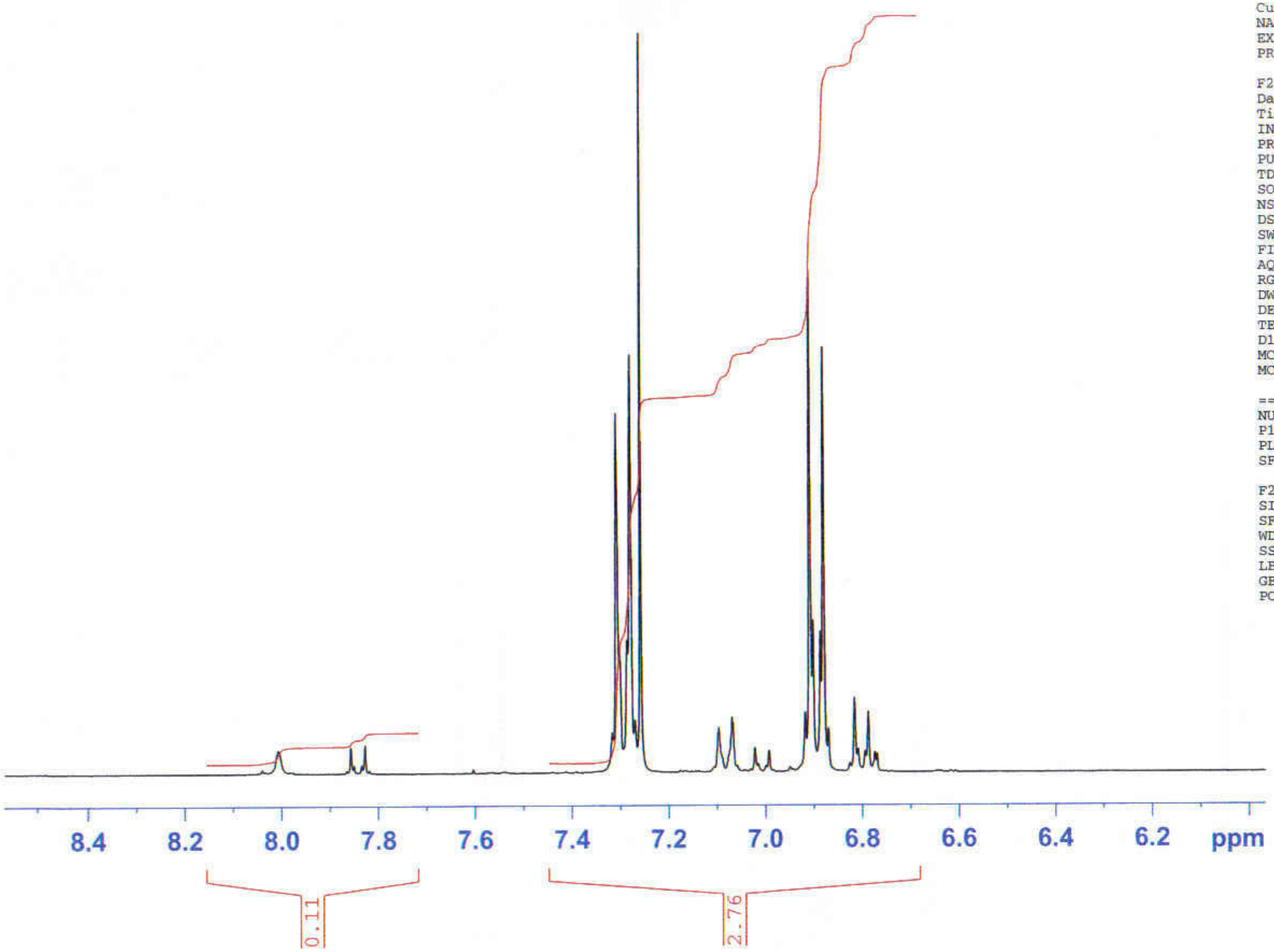

Current Data Parameters		
NAME	10371786	
EXPNO	10	
PROCNO	1	
F2 - Acquisition Parameters		
Date_	20050819	
Time	18.34	
INSTRUM	dpx300	
PROBHD	5 mm QNP 1H/1	
PULPROG	zg30	
TD	48848	
SOLVENT	CDCl3	
NS	64	
DS	2	
SWH	7183.908	Hz
FIDRES	0.147067	Hz
AQ	3.3998709	sec
RG	645.1	
DW	69.600	usec
DE	6.00	usec
TE	299.2	K
D1	6.59999990	sec
MCREST	0.00000000	sec
MCWRK	0.01500000	sec
======== CHANNEL f1 ========		
NUC1	1H	
P1	9.40	usec
PL1	0.00	dB
SFO1	300.1330010	MHz
F2 - Processing parameters		
SI	65536	
SF	300.1300064	MHz
WDW	EM	
SSB	0	
LB	0.30	Hz
GB	0	
PC	2.00	

D. Newt
22/8/05

10368723, NBZ5769-9-3, BRUKER DPX300, QNP, H1 SPECTRUM, SOLV: CDCL3/TMS

Intertek ASG

TABLE 1 ENTRY 7

```
;zg30
;avance-version (02/05/31)
;1D sequence
;using 30 degree flip angle

#include <Avance.incl>

1 ze
2 30m
  d1
  p1*0.33 ph1
  go=2 ph31
  30m mc #0 to 2 F0(zd)
exit

ph1=0 2 2 0 1 3 3 1
ph31=0 2 2 0 1 3 3 1

;pl1 : f1 channel - power level for pulse (defau
;p1 : f1 channel -  90 degree high power pulse
;d1 : relaxation delay; 1-5 * T1
```

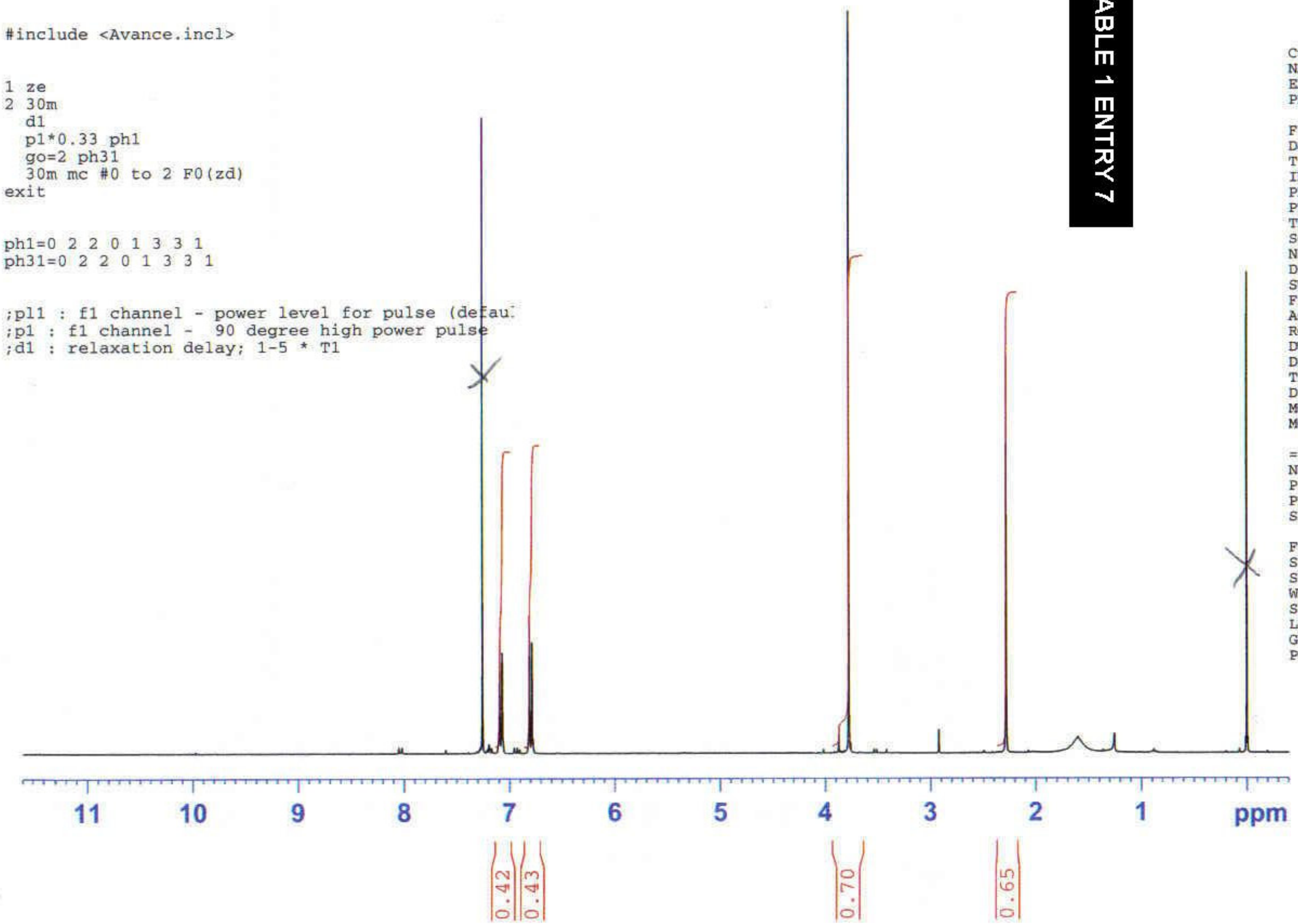


```
Current Data Parameters
NAME              10368723
EXPNO                   10
PROCNO                   1

F2 - Acquisition Parameters
Date_             20050609
Time                 14.46
INSTRUM             dpx300
PROBHD     5 mm QNP  1H/1
PULPROG               zg30
TD                   48848
SOLVENT              CDCl3
NS                      64
DS                       2
SWH               7183.908 Hz
FIDRES            0.147067 Hz
AQ               3.3998709 sec
RG                  1290.2
DW                  69.600 usec
DE                    6.00 usec
TE                   299.2 K
D1              6.59999990 sec
MCREST          0.00000000 sec
MCWRK           0.01500000 sec

======== CHANNEL f1 ========
NUC1                    1H
P1                    9.40 usec
PL1                   0.00 dB
SFO1           300.1330010 MHz

F2 - Processing parameters
SI                   65536
SF             300.1300066 MHz
WDW                     EM
SSB                      0
LB                    0.30 Hz
GB                       0
PC                    2.00
```

D Muff
10/6/05

10368723, NBZ5769-9-3, BRUKER DPX300, QNP, H1 SPECTRUM, SOLV: CDCL3/TMS

Intertek ASG

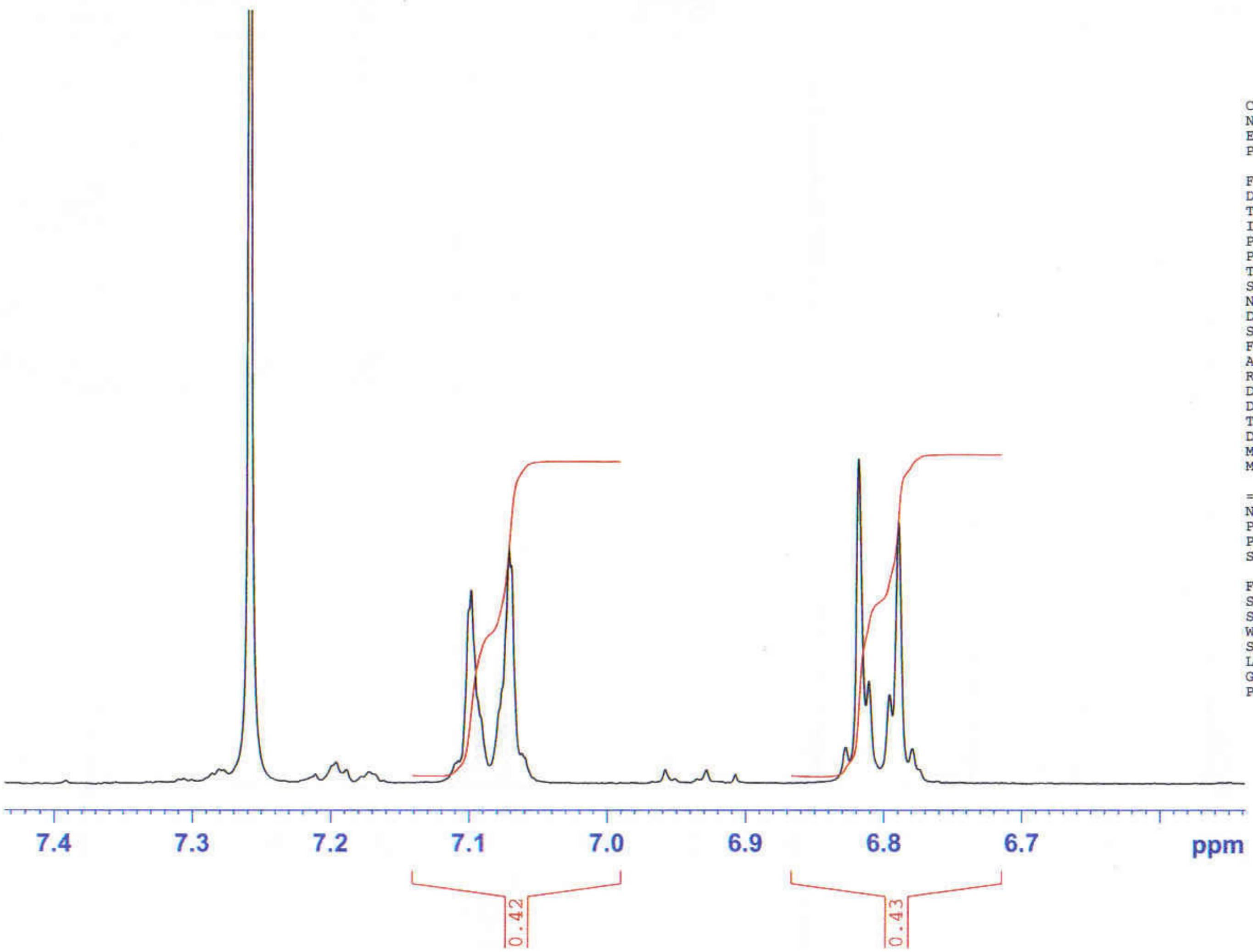

Current Data Parameters	
NAME	10368723
EXPNO	10
PROCNO	1
F2 - Acquisition Parameters	
Date_	20050609
Time	14.46
INSTRUM	dpx300
PROBHD	5 mm QNP 1H/1
PULPROG	zg30
TD	48848
SOLVENT	CDCl3
NS	64
DS	2
SWH	7183.908 Hz
FIDRES	0.147067 Hz
AQ	3.3998709 sec
RG	1290.2
DW	69.600 usec
DE	6.00 usec
TE	299.2 K
D1	6.59999990 sec
MCREST	0.00000000 sec
MCWRK	0.01500000 sec
======== CHANNEL f1 ========	
NUC1	1H
P1	9.40 usec
PL1	0.00 dB
SFO1	300.1330010 MHz
F2 - Processing parameters	
SI	65536
SF	300.1300066 MHz
WDW	EM
SSB	0
LB	0.30 Hz
GB	0
PC	2.00

D. Nlwtt [illegible]
10/6/05

10371787, NBZ5769/42/B, BRUKER DPX300, QNP. H1 SPECTRUM, SOLV:-CDCL3/TMS

Intertek ASG

TABLE 1 ENTRY 8

```
;zg30
;avance-version (02/05/31)
;1D sequence
;using 30 degree flip angle

#include <Avance.incl>

1 ze
2 30m
  d1
  p1*0.33 ph1
  go=2 ph31
  30m mc #0 to 2 F0(zd)
exit

ph1=0 2 2 0 1 3 3 1
ph31=0 2 2 0 1 3 3 1

;pl1 : f1 channel - power level for pulse (defaul
;p1 : f1 channel -  90 degree high power pulse
;d1 : relaxation delay; 1-5 * T1
```

```
Current Data Parameters
NAME          10371787
EXPNO               10
PROCNO               1

F2 - Acquisition Parameters
Date_         20050819
Time             18.51
INSTRUM         dpx300
PROBHD   5 mm QNP  1H/1
PULPROG           zg30
TD               48848
SOLVENT          CDC13
NS                  64
DS                   2
SWH           7183.908 Hz
FIDRES        0.147067 Hz
AQ           3.3998709 sec
RG                 512
DW              69.600 usec
DE                6.00 usec
TE               299.2 K
D1          6.59999990 sec
MCREST      0.00000000 sec
MCWRK       0.01500000 sec

======== CHANNEL f1 ========
NUC1                1H
P1                9.40 usec
PL1               0.00 dB
SFO1       300.1330010 MHz

F2 - Processing parameters
SI               65536
SF         300.1300078 MHz
WDW                 EM
SSB                  0
LB                0.30 Hz
GB                   0
PC                2.00
```

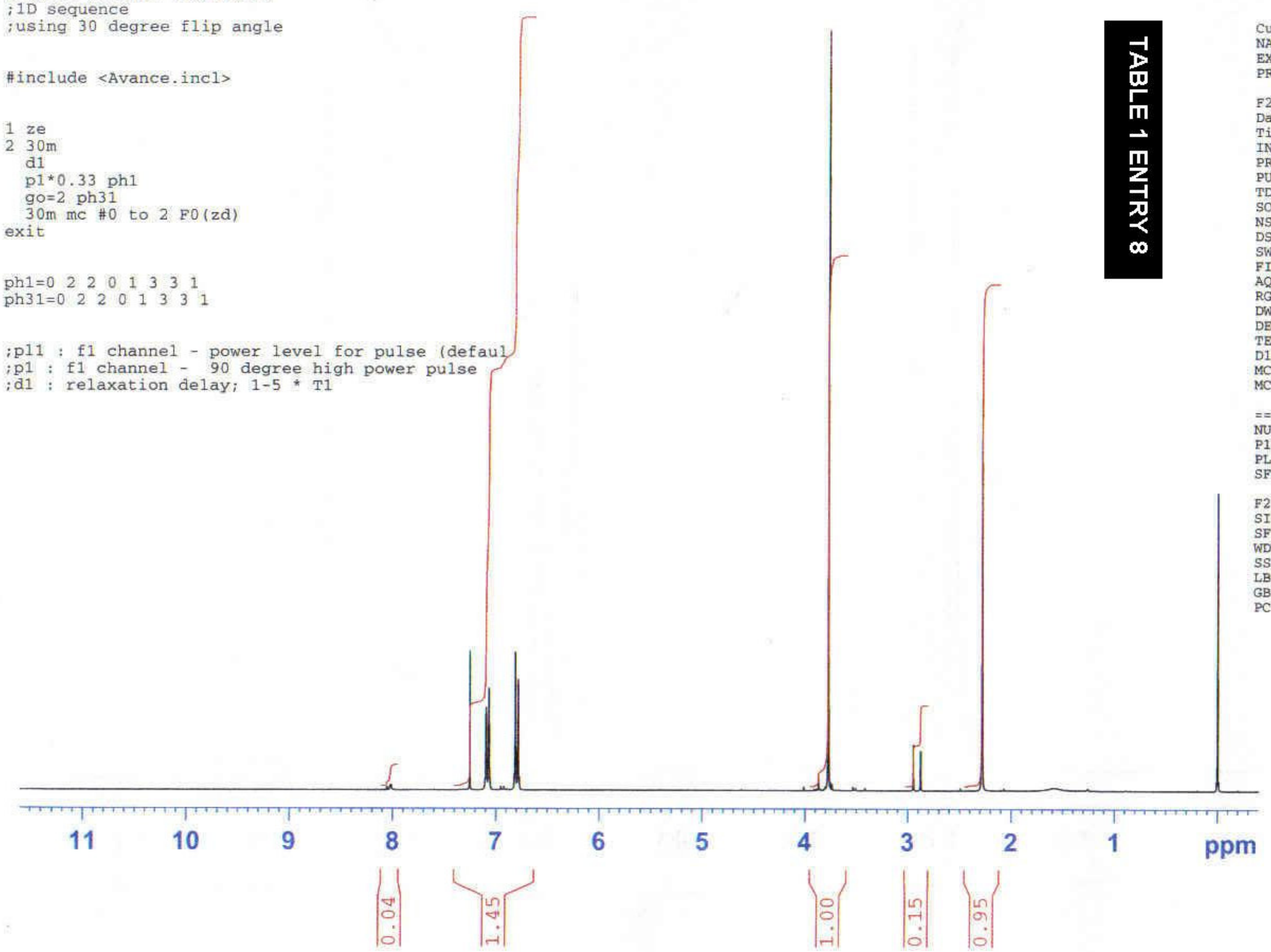

10371787, NBZ5769/42/B, BRUKER DPX300, QNP. H1 SPECTRUM, SOLV:-CDCL3/TMS

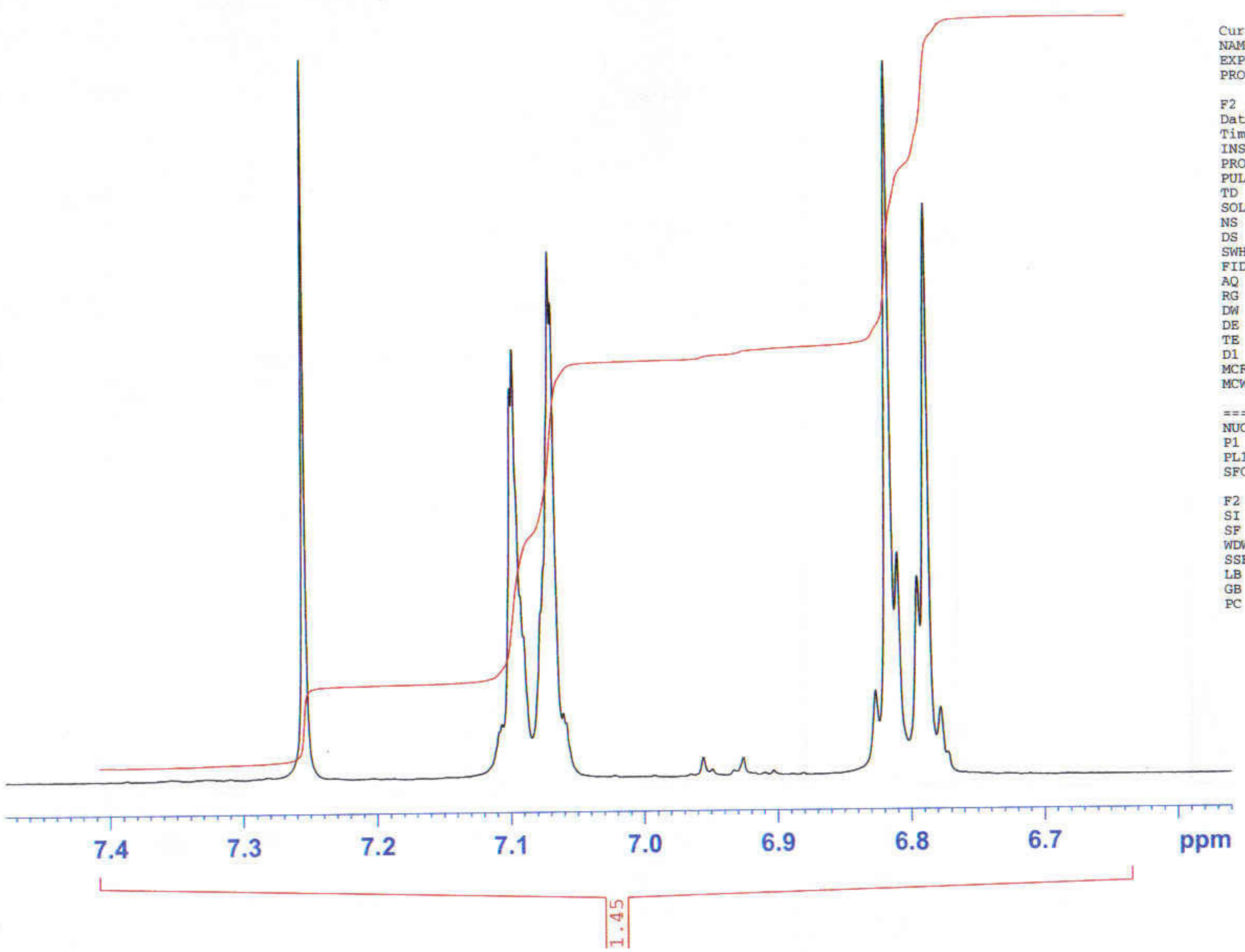


```
Current Data Parameters
NAME              10371787
EXPNO                   10
PROCNO                   1

F2 - Acquisition Parameters
Date_             20050819
Time                 18.51
INSTRUM             dpx300
PROBHD      5 mm QNP  1H/1
PULPROG               zg30
TD                   48848
SOLVENT              CDCl3
NS                      64
DS                       2
SWH               7183.908 Hz
FIDRES            0.147067 Hz
AQ               3.3998709 sec
RG                     512
DW                  69.600 usec
DE                    6.00 usec
TE                   299.2 K
D1              6.59999990 sec
MCREST          0.00000000 sec
MCWRK           0.01500000 sec

======== CHANNEL f1 ========
NUC1                    1H
P1                    9.40 usec
PL1                   0.00 dB
SFO1           300.1330010 MHz

F2 - Processing parameters
SI                   65536
SF             300.1300078 MHz
WDW                     EM
SSB                      0
LB                    0.30 Hz
GB                       0
PC                    2.00
```

10368721, NBZ5769-9-1, BRUKER DPX300, QNP, H1 SPECTRUM, SOLV: CDCL3/TMS

Intertek ASG

TABLE 1 ENTRY 9

```
;zg30
;avance-version (02/05/31)
;1D sequence
;using 30 degree flip angle

#include <Avance.incl>

1 ze
2 30m
  d1
  p1*0.33 ph1
  go=2 ph31
  30m mc #0 to 2 F0(zd)
exit

ph1=0 2 2 0 1 3 3 1
ph31=0 2 2 0 1 3 3 1

;pl1 : f1 channel - power level for pulse (defau
;p1 : f1 channel -  90 degree high power pulse
;d1 : relaxation delay; 1-5 * T1
```

```
Current Data Parameters
NAME            10368721
EXPNO                 10
PROCNO                 1

F2 - Acquisition Parameters
Date_           20050609
Time               14.12
INSTRUM           dpx300
PROBHD    5 mm QNP  1H/1
PULPROG             zg30
TD                 48848
SOLVENT            CDCl3
NS                    64
DS                     2
SWH             7183.908 Hz
FIDRES          0.147067 Hz
AQ             3.3998709 sec
RG                 724.1
DW                69.600 usec
DE                  6.00 usec
TE                 299.2 K
D1            6.59999990 sec
MCREST        0.00000000 sec
MCWRK         0.01500000 sec

======== CHANNEL f1 ========
NUC1                  1H
P1                  9.40 usec
PL1                 0.00 dB
SFO1         300.1330010 MHz

F2 - Processing parameters
SI                 65536
SF           300.1300065 MHz
WDW                   EM
SSB                    0
LB                  0.30 Hz
GB                     0
PC                  2.00
```

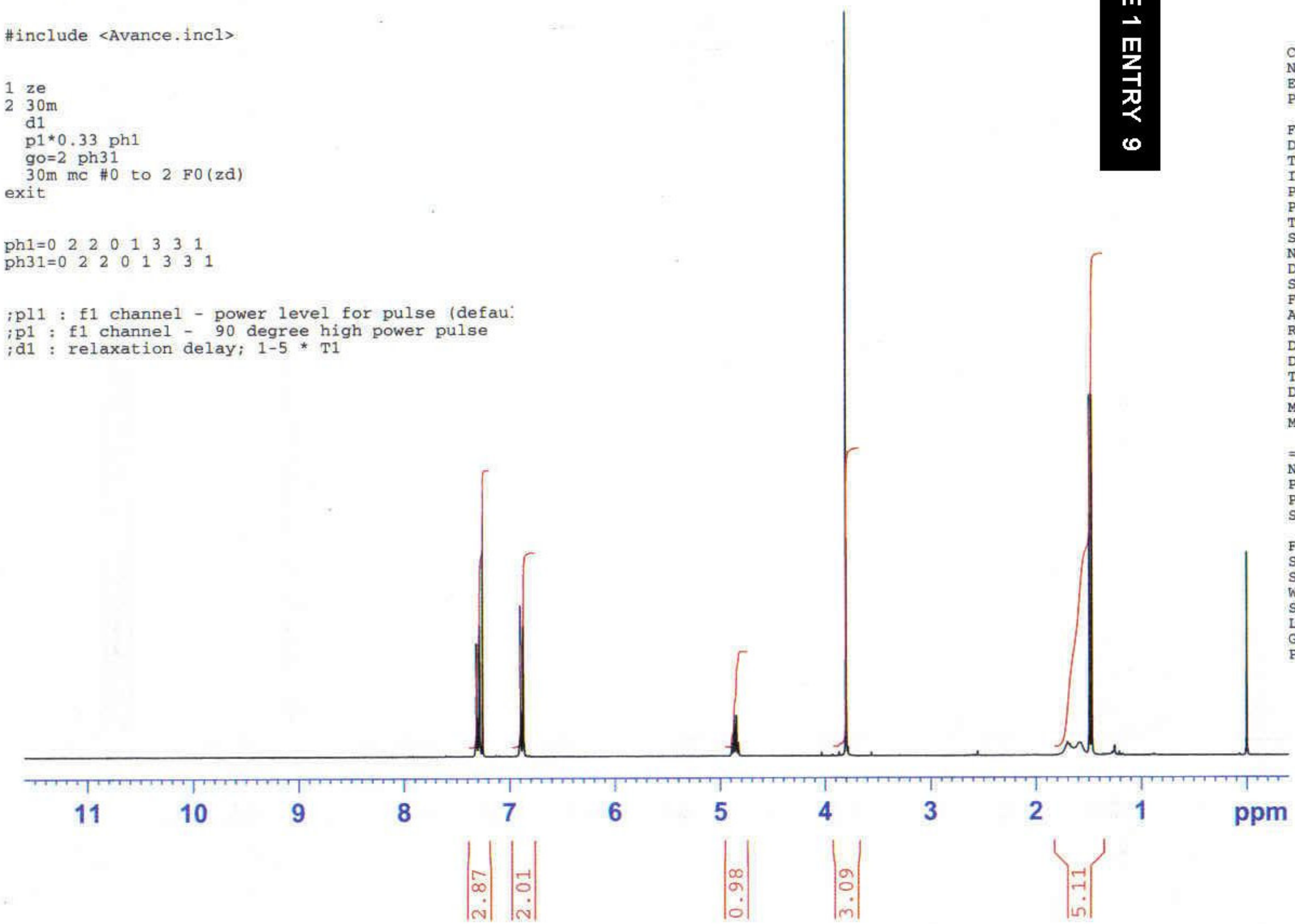

D. Nut 10/6/05

10368721, NBZ5769-9-1, BRUKER DPX300, QNP, H1 SPECTRUM, SOLV: CDCL3/TMS

Intertek ASG

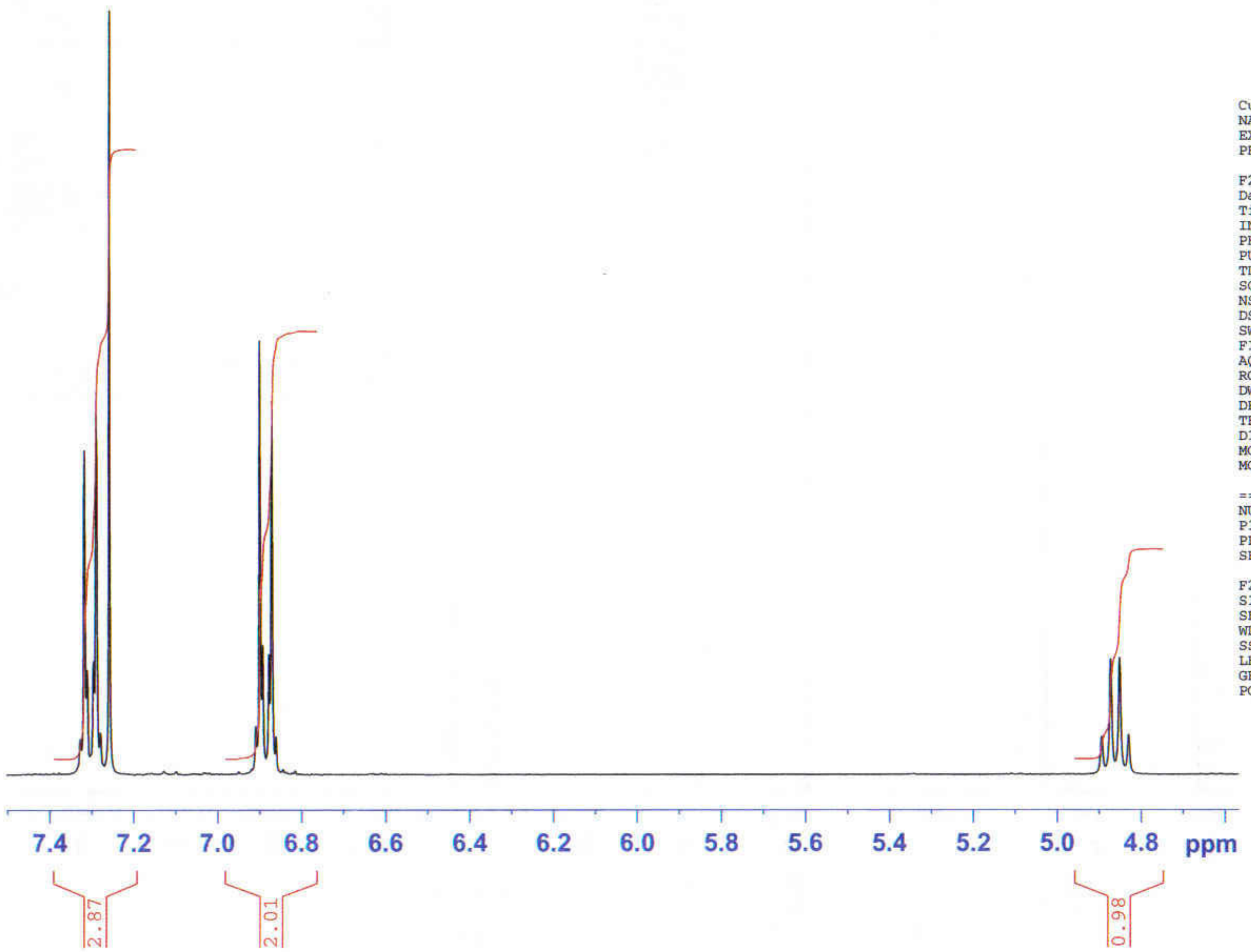

Current Data Parameters	
NAME	10368721
EXPNO	10
PROCNO	1
F2 - Acquisition Parameters	
Date_	20050609
Time	14.12
INSTRUM	dpx300
PROBHD	5 mm QNP 1H/1
PULPROG	zg30
TD	48848
SOLVENT	CDCl3
NS	64
DS	2
SWH	7183.908 Hz
FIDRES	0.147067 Hz
AQ	3.3998709 sec
RG	724.1
DW	69.600 usec
DE	6.00 usec
TE	299.2 K
D1	6.59999990 sec
MCREST	0.00000000 sec
MCWRK	0.01500000 sec
======== CHANNEL f1 ========	
NUC1	1H
P1	9.40 usec
PL1	0.00 dB
SFO1	300.1330010 MHz
F2 - Processing parameters	
SI	65536
SF	300.1300065 MHz
WDW	EM
SSB	0
LB	0.30 Hz
GB	0
PC	2.00

[illegible]
10/6/05

10383512, RX0002/40A, BRUKER DPX300, QNP, H1 SPECTRUM, SOLV;- CDCl3/TMS

TABLE 2 ENTRY 1

```
;zg30
;avance-version (02/05/31)
;1D sequence
;using 30 degree flip angle

#include <Avance.incl>

1 ze
2 30m
  d1
  p1*0.33 ph1
  go=2 ph31
  30m mc #0 to 2 F0(zd)
exit

ph1=0 2 2 0 1 3 3 1
ph31=0 2 2 0 1 3 3 1

;pl1 : f1 channel - power leve
;p1 : f1 channel -  90 degree
;d1 : relaxation delay; 1-5 *
```

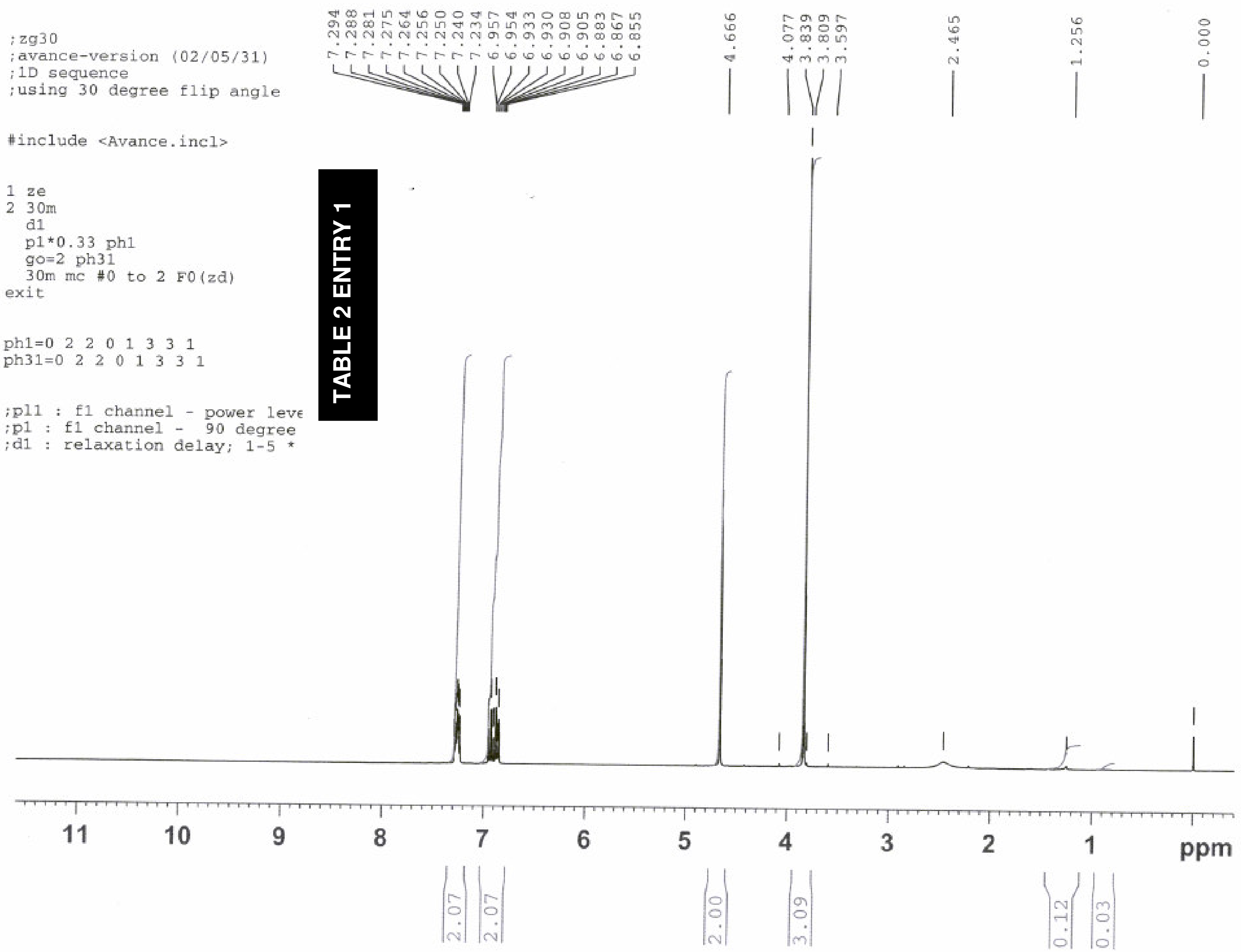

Intertek ASG

Current Data Parameters	
NAME	10383512
EXPNO	10
PROCNO	1
**F2 - Acquisition Parameters**	
Date_	20060713
Time	12.01
INSTRUM	dpx300
PROBHD	5 mm QNP 1H/13
PULPROG	zg30
TD	48848
SOLVENT	CDCl3
NS	64
DS	2
SWH	7183.908 Hz
FIDRES	0.147067 Hz
AQ	3.3998709 sec
RG	101.6
DW	69.600 usec
DE	6.00 usec
TE	299.2 K
D1	6.59999990 sec
MCREST	0.00000000 sec
MCWRK	0.01500000 sec
**======== CHANNEL f1 ========**	
NUC1	1H
P1	9.40 usec
PL1	0.00 dB
SFO1	300.1330010 MHz
**F2 - Processing parameters**	
SI	65536
SF	300.1300116 MHz
WDW	EM
SSB	0
LB	0.30 Hz
GB	0
PC	300.00

S.N

13/7/06

10383512, RX0002/40A, BRUKER DPX300, QNP, H1 SPECTRUM, SOLV;- CDCl3/TMS

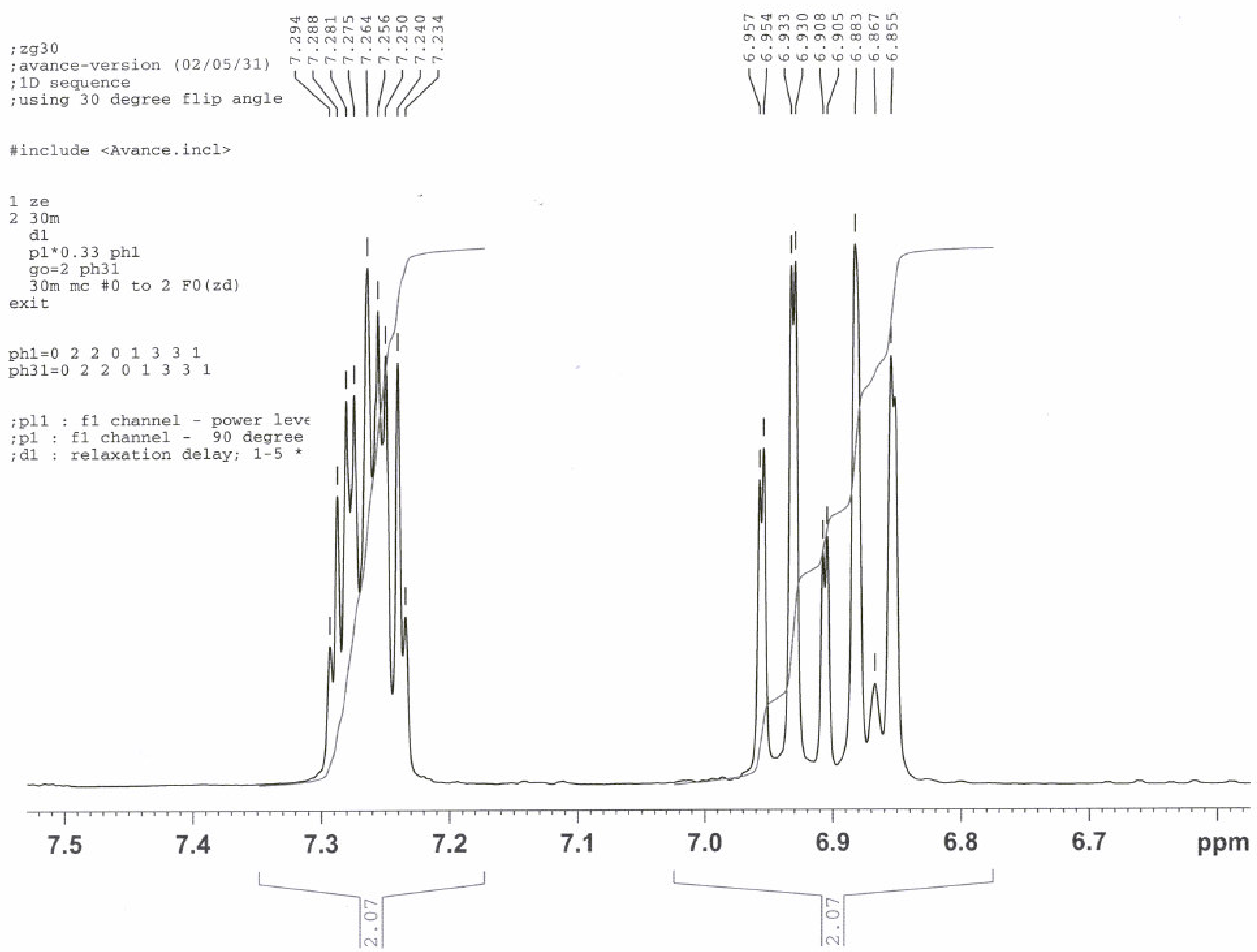

Intertek ASG

Current Data Parameters	
NAME	10383512
EXPNO	10
PROCNO	1

F2 - Acquisition Parameters	
Date_	20060713
Time	12.01
INSTRUM	dpx300
PROBHD	5 mm QNP 1H/13
PULPROG	zg30
TD	48848
SOLVENT	CDCl3
NS	64
DS	2
SWH	7183.908 Hz
FIDRES	0.147067 Hz
AQ	3.3998709 sec
RG	101.6
DW	69.600 usec
DE	6.00 usec
TE	299.2 K
D1	6.59999990 sec
MCREST	0.00000000 sec
MCWRK	0.01500000 sec

======== CHANNEL f1 ========	
NUC1	1H
P1	9.40 usec
PL1	0.00 dB
SFO1	300.1330010 MHz

F2 - Processing parameters	
SI	65536
SF	300.1300116 MHz
WDW	EM
SSB	0
LB	0.30 Hz
GB	0
PC	300.00

S.N
13/7/06

10383512, RX0002/40A, BRUKER DPX300, QNP, H1 SPECTRUM, SOLV;- CDCl3/TMS

```
;zg30
;avance-version (02/05/31)
;1D sequence
;using 30 degree flip angle

#include <Avance.incl>

1 ze
2 30m
  d1
  p1*0.33 ph1
  go=2 ph31
  30m mc #0 to 2 F0(zd)
exit

ph1=0 2 2 0 1 3 3 1
ph31=0 2 2 0 1 3 3 1

;pl1 : f1 channel - power leve
;p1 : f1 channel -  90 degree
;d1 : relaxation delay; 1-5 *
```

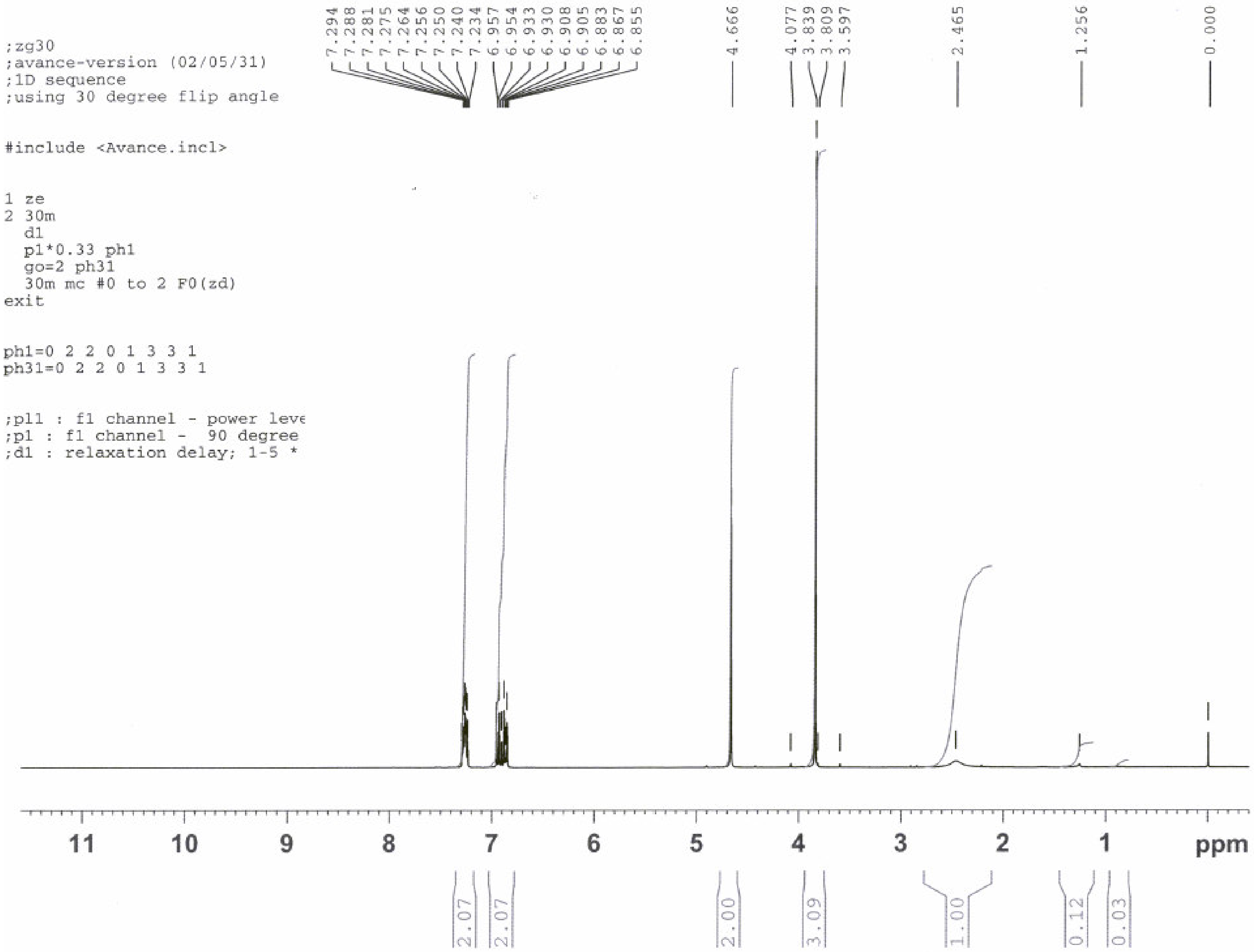

Intertek ASG

Current Data Parameters	
NAME	10383512
EXPNO	10
PROCNO	1

F2 - Acquisition Parameters	
Date_	20060713
Time	12.01
INSTRUM	dpx300
PROBHD	5 mm QNP 1H/13
PULPROG	zg30
TD	48848
SOLVENT	CDCl3
NS	64
DS	2
SWH	7183.908 Hz
FIDRES	0.147067 Hz
AQ	3.3998709 sec
RG	101.6
DW	69.600 usec
DE	6.00 usec
TE	299.2 K
D1	6.59999990 sec
MCREST	0.00000000 sec
MCWRK	0.01500000 sec

======== CHANNEL f1 ========	
NUC1	1H
P1	9.40 usec
PL1	0.00 dB
SFO1	300.1330010 MHz

F2 - Processing parameters	
SI	65536
SF	300.1300116 MHz
WDW	EM
SSB	0
LB	0.30 Hz
GB	0
PC	300.00

S.M 20/7/06

10383516, RX0002/40D, BRUKER DPX300, QNP, H1 SPECTRUM, SOLV;- CDCl3/TMS

```
;zg30
;avance-version (02/05/31)
;1D sequence
;using 30 degree flip angle

#include <Avance.incl>

1 ze
2 30m
  d1
  p1*0.33 ph1
  go=2 ph31
  30m mc #0 to 2 F0(zd)
exit

ph1=0 2 2 0 1 3 3 1
ph31=0 2 2 0 1 3 3 1

;pl1 : f1 channel - power leve
;p1  : f1 channel -  90 degree
;d1  : relaxation delay; 1-5 *
```

TABLE 2 ENTRY 2

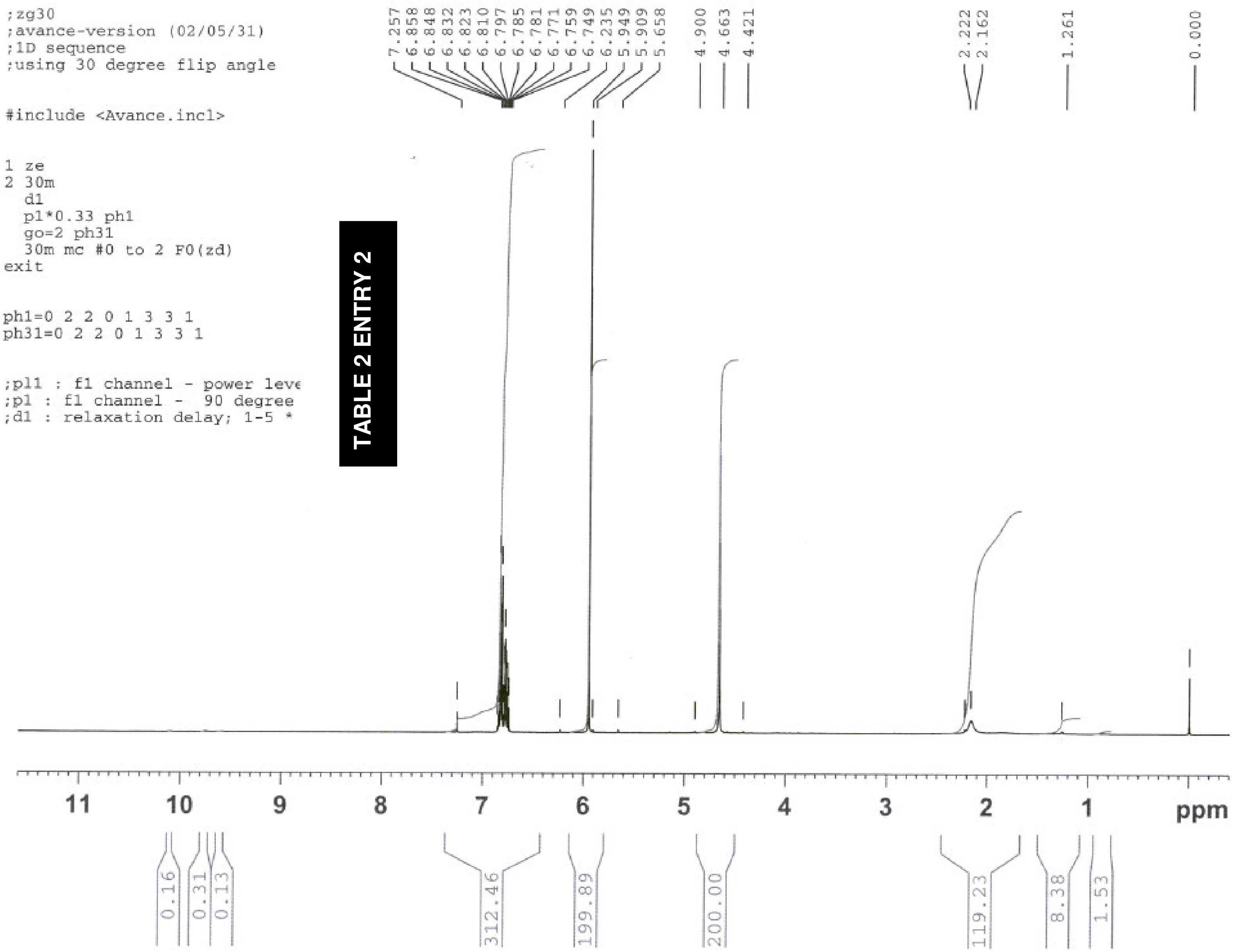

Intertek ASG

**Current Data Parameters**	
NAME	10383516
EXPNO	10
PROCNO	1
**F2 - Acquisition Parameters**	
Date_	20060713
Time	12.54
INSTRUM	dpx300
PROBHD	5 mm QNP 1H/13
PULPROG	zg30
TD	48848
SOLVENT	CDCl3
NS	64
DS	2
SWH	7183.908 Hz
FIDRES	0.147067 Hz
AQ	3.3998709 sec
RG	128
DW	69.600 usec
DE	6.00 usec
TE	299.2 K
D1	6.59999990 sec
MCREST	0.00000000 sec
MCWRK	0.01500000 sec
======== CHANNEL f1 ========	
NUC1	1H
P1	9.40 usec
PL1	0.00 dB
SFO1	300.1330010 MHz
**F2 - Processing parameters**	
SI	65536
SF	300.1300070 MHz
WDW	EM
SSB	0
LB	0.30 Hz
GB	0
PC	100.00

S.[illegible]

13/7/06

10383614, RX0002/42A, BRUKER DPX300, QNP, H1 SPECTRUM, SOLV:-CDCL3/TMS

```
;zg30
;avance-version (02/05/31)
;1D sequence
;using 30 degree flip angle

#include <Avance.incl>

1 ze
2 30m
  d1
  p1*0.33 ph1
  go=2 ph31
  30m mc #0 to 2 F0(zd)
exit

ph1=0 2 2 0 1 3 3 1
ph31=0 2 2 0 1 3 3 1

;pl1 : f1 channel - power leve
;p1 : f1 channel -  90 degree
;d1 : relaxation delay; 1-5 *
```

TABLE 2 ENTRY 3

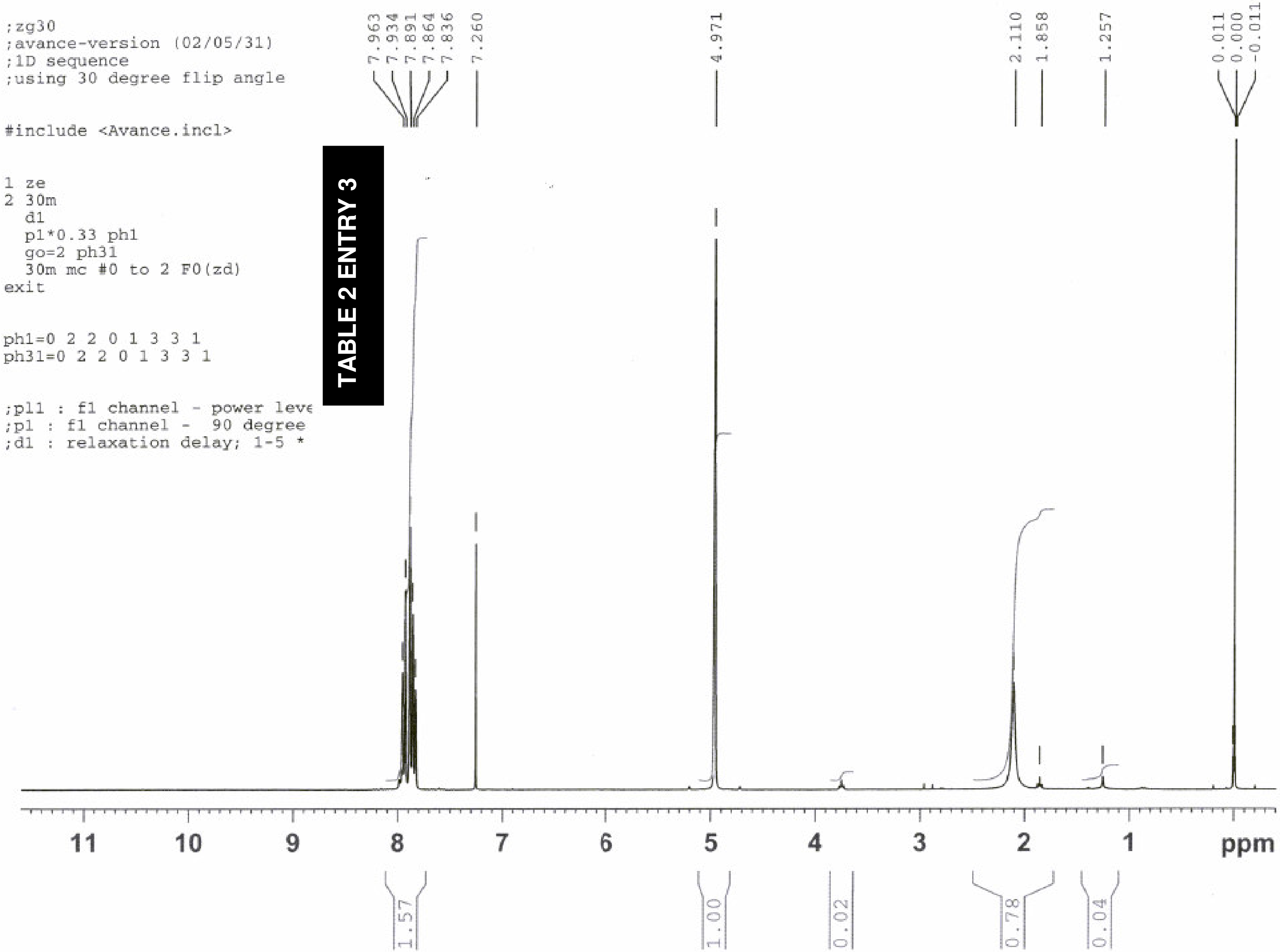

Intertek ASG

```
Current Data Parameters
NAME              10383614
EXPNO                   10
PROCNO                   1

F2 - Acquisition Parameters
Date_             20060717
Time                 12.53
INSTRUM             dpx300
PROBHD     5 mm QNP 1H/13
PULPROG               zg30
TD                   48848
SOLVENT              CDCl3
NS                      64
DS                       2
SWH              7183.908 Hz
FIDRES           0.147067 Hz
AQ              3.3998709 sec
RG                   724.1
DW                  69.600 usec
DE                    6.00 usec
TE                   299.2 K
D1             6.59999990 sec
MCREST         0.00000000 sec
MCWRK          0.01500000 sec

======== CHANNEL f1 ========
NUC1                    1H
P1                    9.40 usec
PL1                   0.00 dB
SFO1           300.1330010 MHz

F2 - Processing parameters
SI                   65536
SF             300.1300061 MHz
WDW                     EM
SSB                      0
LB                    0.30 Hz
GB                       0
PC                    2.00
```

D. [illegible]
18/7/06

10383614, RX0002/42A, BRUKER DPX300, QNP, H1 SPECTRUM, SOLV:-CDCL3/TMS

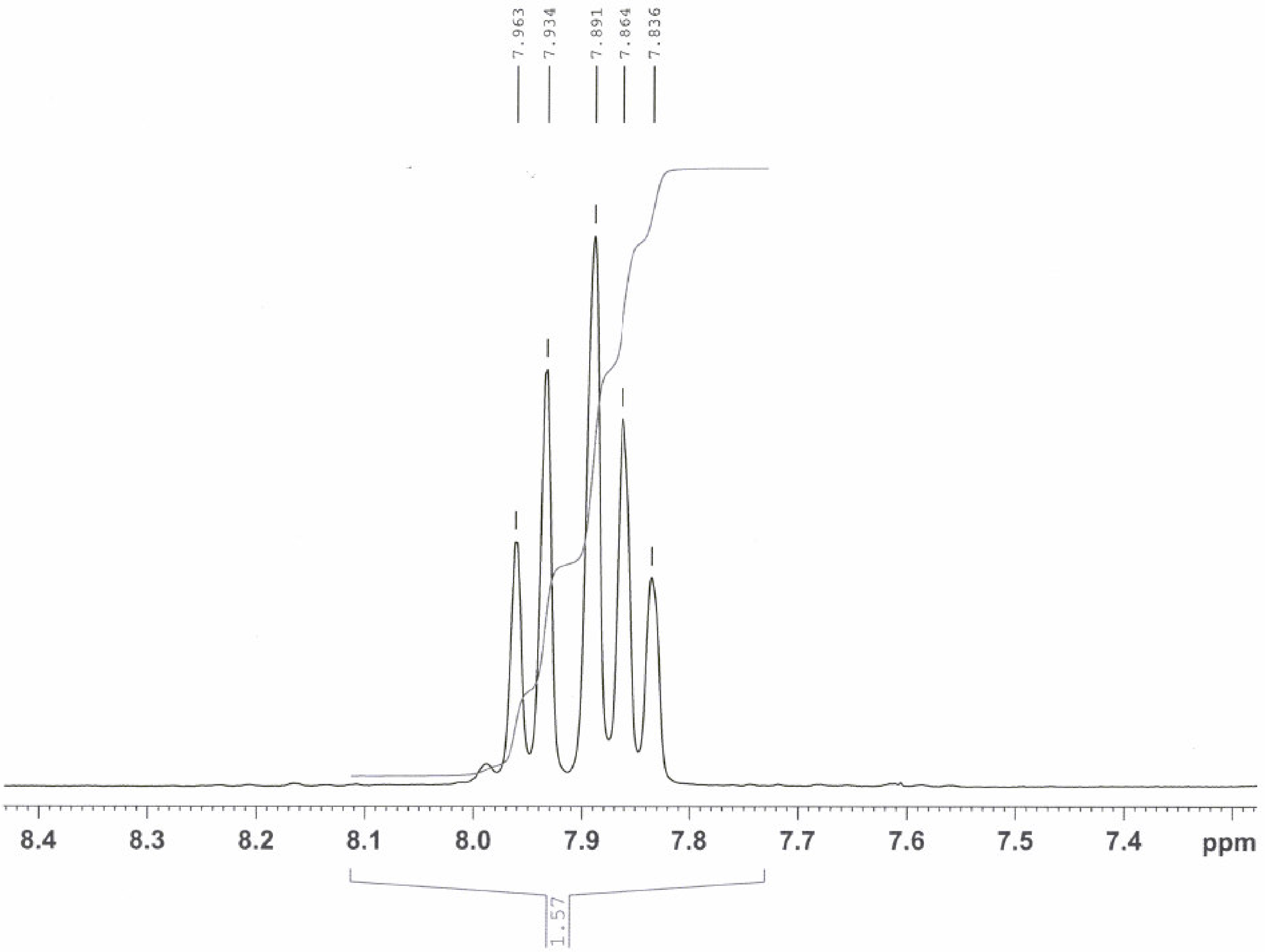

Intertek ASG

Current Data Parameters	
NAME	10383614
EXPNO	10
PROCNO	1
F2 - Acquisition Parameters	
Date_	20060717
Time	12.53
INSTRUM	dpx300
PROBHD	5 mm QNP 1H/13
PULPROG	zg30
TD	48848
SOLVENT	CDCl3
NS	64
DS	2
SWH	7183.908 Hz
FIDRES	0.147067 Hz
AQ	3.3998709 sec
RG	724.1
DW	69.600 usec
DE	6.00 usec
TE	299.2 K
D1	6.59999990 sec
MCREST	0.00000000 sec
MCWRK	0.01500000 sec
======== CHANNEL f1 ========	
NUC1	1H
P1	9.40 usec
PL1	0.00 dB
SFO1	300.1330010 MHz
F2 - Processing parameters	
SI	65536
SF	300.1300061 MHz
WDW	EM
SSB	0
LB	0.30 Hz
GB	0
PC	2.00

TABLE 2 ENTRY 4

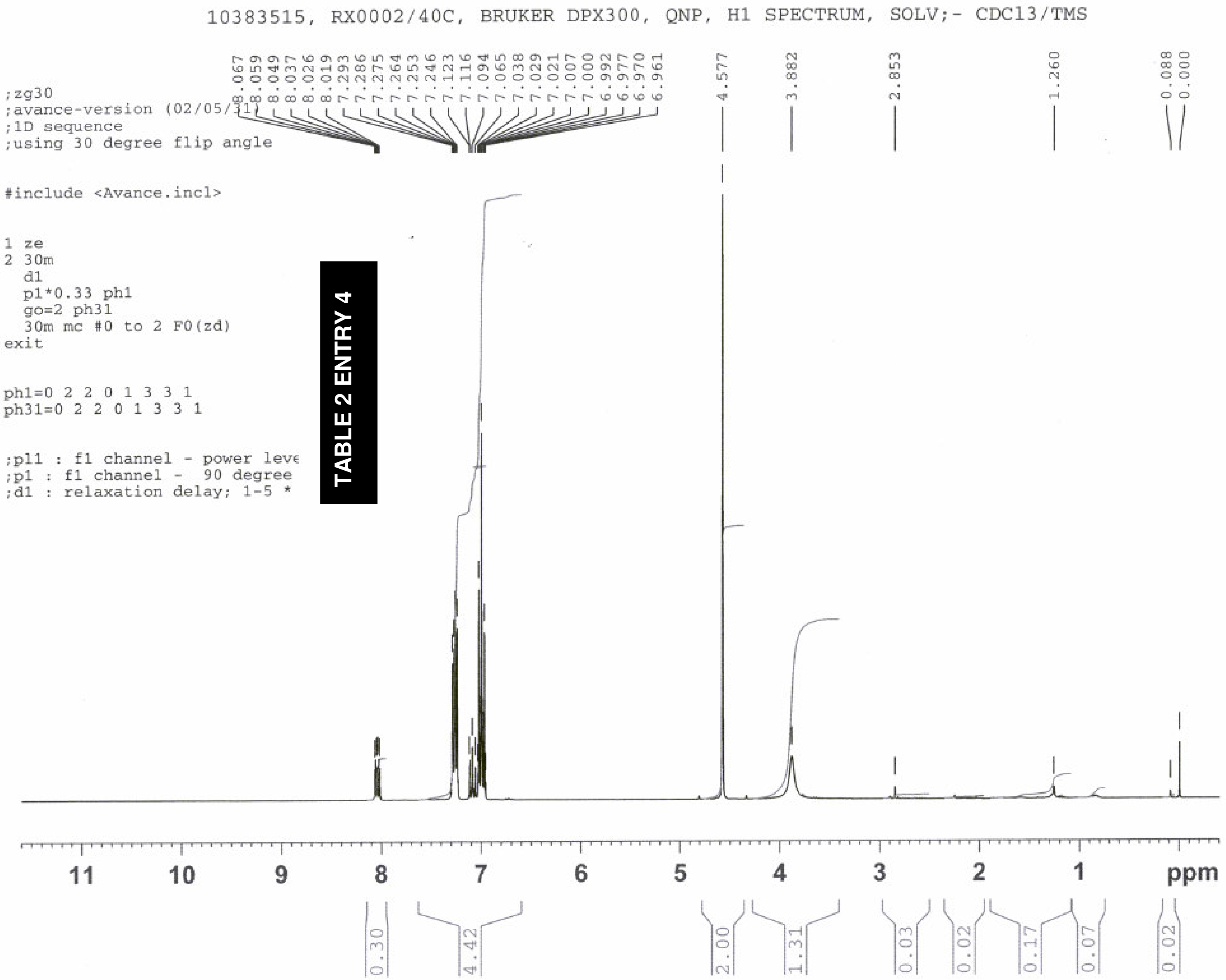

Intertek ASG

```
Current Data Parameters
NAME              10383515
EXPNO                   10
PROCNO                   1

F2 - Acquisition Parameters
Date_             20060713
Time                 13.12
INSTRUM             dpx300
PROBHD   5 mm QNP 1H/13
PULPROG               zg30
TD                   48848
SOLVENT              CDCl3
NS                      64
DS                       2
SWH              7183.908 Hz
FIDRES           0.147067 Hz
AQ              3.3998709 sec
RG                    71.8
DW                  69.600 usec
DE                    6.00 usec
TE                   299.2 K
D1             6.59999990 sec
MCREST         0.00000000 sec
MCWRK          0.01500000 sec

======== CHANNEL f1 ========
NUC1                    1H
P1                    9.40 usec
PL1                   0.00 dB
SFO1           300.1330010 MHz

F2 - Processing parameters
SI                   65536
SF             300.1300091 MHz
WDW                     EM
SSB                      0
LB                    0.30 Hz
GB                       0
PC                  250.00
```

S.n
13/7/06

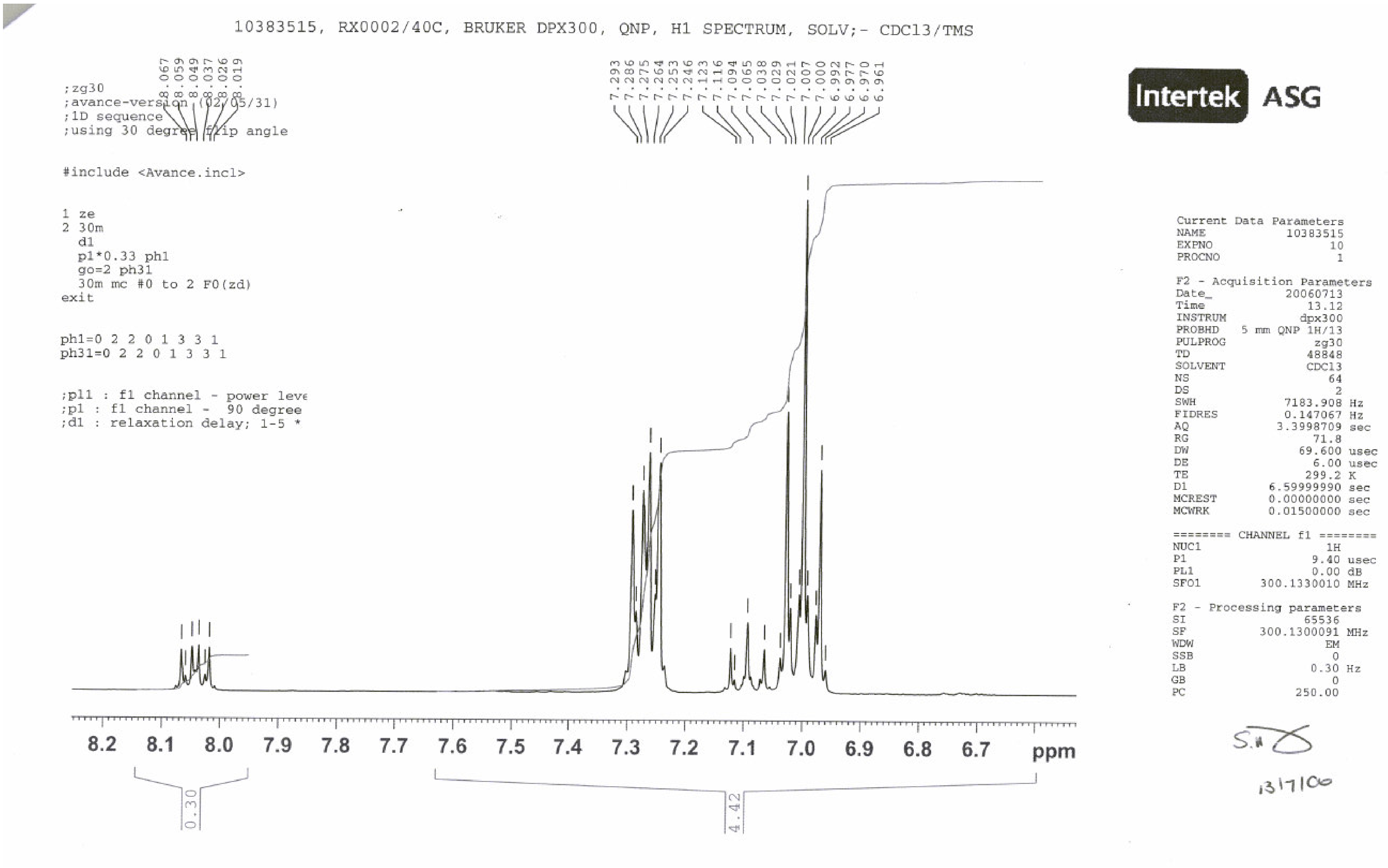
10383515, RX0002/40C, BRUKER DPX300, QNP, H1 SPECTRUM, SOLV;- CDC13/TMS
;zg30
;avance-version (02/05/31)
;1D sequence
;using 30 degree flip angle
#include <Avance.incl>
1 ze
2 30m
d1
p1*0.33 ph1
go=2 ph31
30m mc #0 to 2 F0(zd)
exit
ph1=0 2 2 0 1 3 3 1
ph31=0 2 2 0 1 3 3 1
;pl1 : f1 channel - power leve
;p1 : f1 channel - 90 degree
;d1 : relaxation delay; 1-5 *
8.067
8.059
8.049
8.037
8.026
8.019
7.293
7.286
7.275
7.264
7.253
7.246
7.123
7.116
7.094
7.065
7.038
7.029
7.021
7.007
7.000
6.992
6.977
6.970
6.961
8.2
8.1
8.0
7.9
7.8
7.7
7.6
7.5
7.4
7.3
7.2
7.1
7.0
6.9
6.8
6.7
ppm
0.30
4.42
Intertek ASG
Current Data Parameters
NAME 10383515
EXPNO 10
PROCNO 1
F2 - Acquisition Parameters
Date_ 20060713
Time 13.12
INSTRUM dpx300
PROBHD 5 mm QNP 1H/13
PULPROG zg30
TD 48848
SOLVENT CDC13
NS 64
DS 2
SWH 7183.908 Hz
FIDRES 0.147067 Hz
AQ 3.3998709 sec
RG 71.8
DW 69.600 usec
DE 6.00 usec
TE 299.2 K
D1 6.59999990 sec
MCREST 0.00000000 sec
MCWRK 0.01500000 sec
======== CHANNEL f1 ========
NUC1 1H
P1 9.40 usec
PL1 0.00 dB
SFO1 300.1330010 MHz
F2 - Processing parameters
SI 65536
SF 300.1300091 MHz
WDW EM
SSB 0
LB 0.30 Hz
GB 0
PC 250.00
13/7/06

10368288, NBZ5769/1/8, BRUKER DPX300, QNP, H1 SPECTRUM, SOLV:- CDCL3/TMS

Intertek ASG

Table 3 Entry 1

```
;zg30
;avance-version (02/05/31)
;1D sequence
;using 30 degree flip angle

#include <Avance.incl>

1 ze
2 30m
  d1
  p1*0.33 ph1
  go=2 ph31
  30m mc #0 to 2 F0(zd)
exit

ph1=0 2 2 0 1 3 3 1
ph31=0 2 2 0 1 3 3 1

;pl1 : f1 channel - power level for pulse (defau
;p1 : f1 channel -  90 degree high power pulse
;d1 : relaxation delay; 1-5 * T1
```

```
Current Data Parameters
NAME             10368288
EXPNO                  10
PROCNO                  1

F2 - Acquisition Parameters
Date_            20050526
Time                21.12
INSTRUM            dpx300
PROBHD   5 mm QNP  1H/1
PULPROG              zg30
TD                  48848
SOLVENT             CDCl3
NS                     64
DS                      2
SWH              7183.908 Hz
FIDRES           0.147067 Hz
AQ              3.3998709 sec
RG                  724.1
DW                 69.600 usec
DE                   6.00 usec
TE                  299.2 K
D1             6.59999990 sec
MCREST         0.00000000 sec
MCWRK          0.01500000 sec

======== CHANNEL f1 ========
NUC1                   1H
P1                   9.40 usec
PL1                  0.00 dB
SFO1          300.1330010 MHz

F2 - Processing parameters
SI                  65536
SF            300.1300079 MHz
WDW                    EM
SSB                     0
LB                   0.30 Hz
GB                      0
PC                   2.00
```

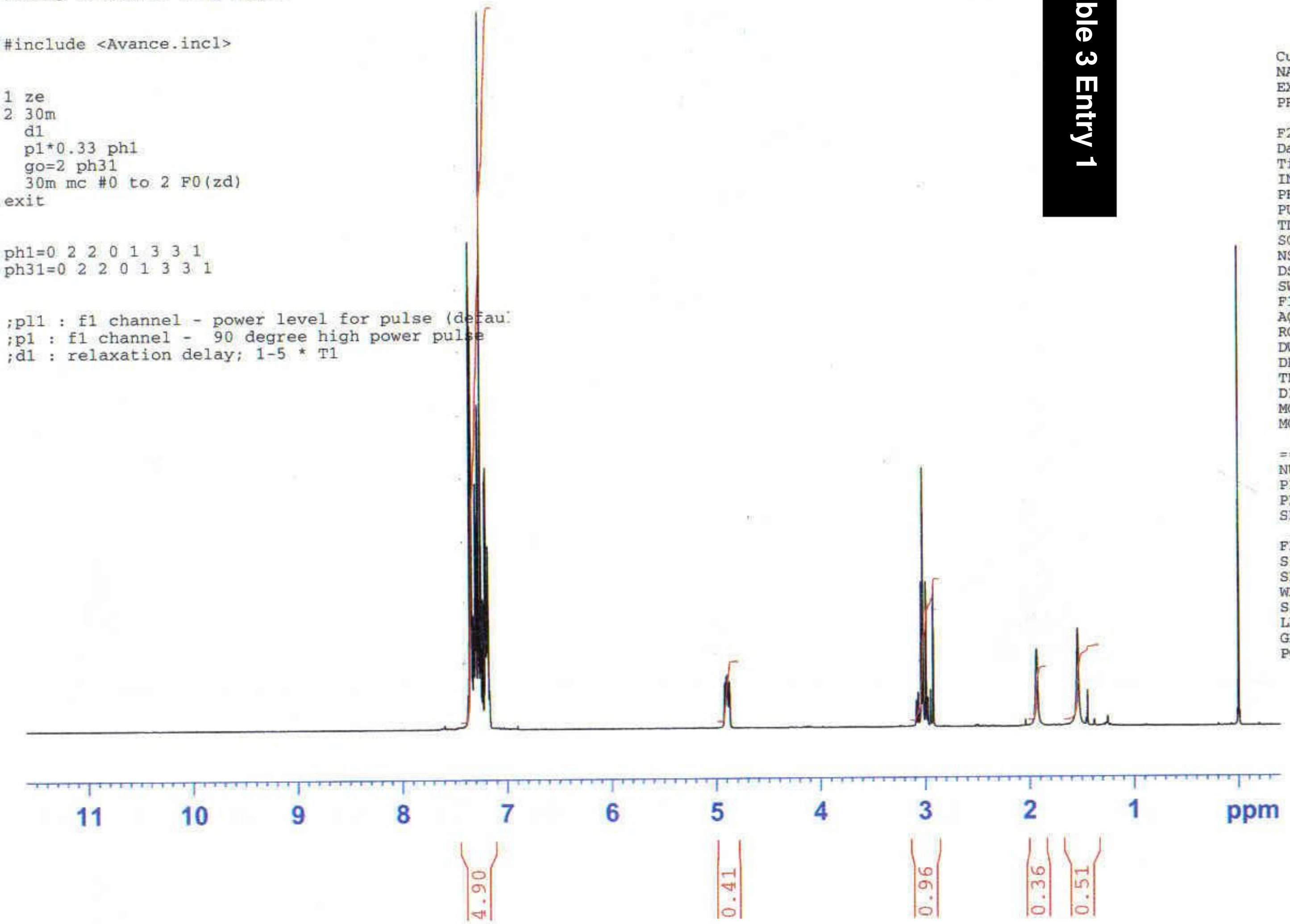

D. Mertz 27/5/05

10368288, NBZ5769/1/8, BRUKER DPX300, QNP, H1 SPECTRUM, SOLV:- CDCL3/TMS

Intertek ASG

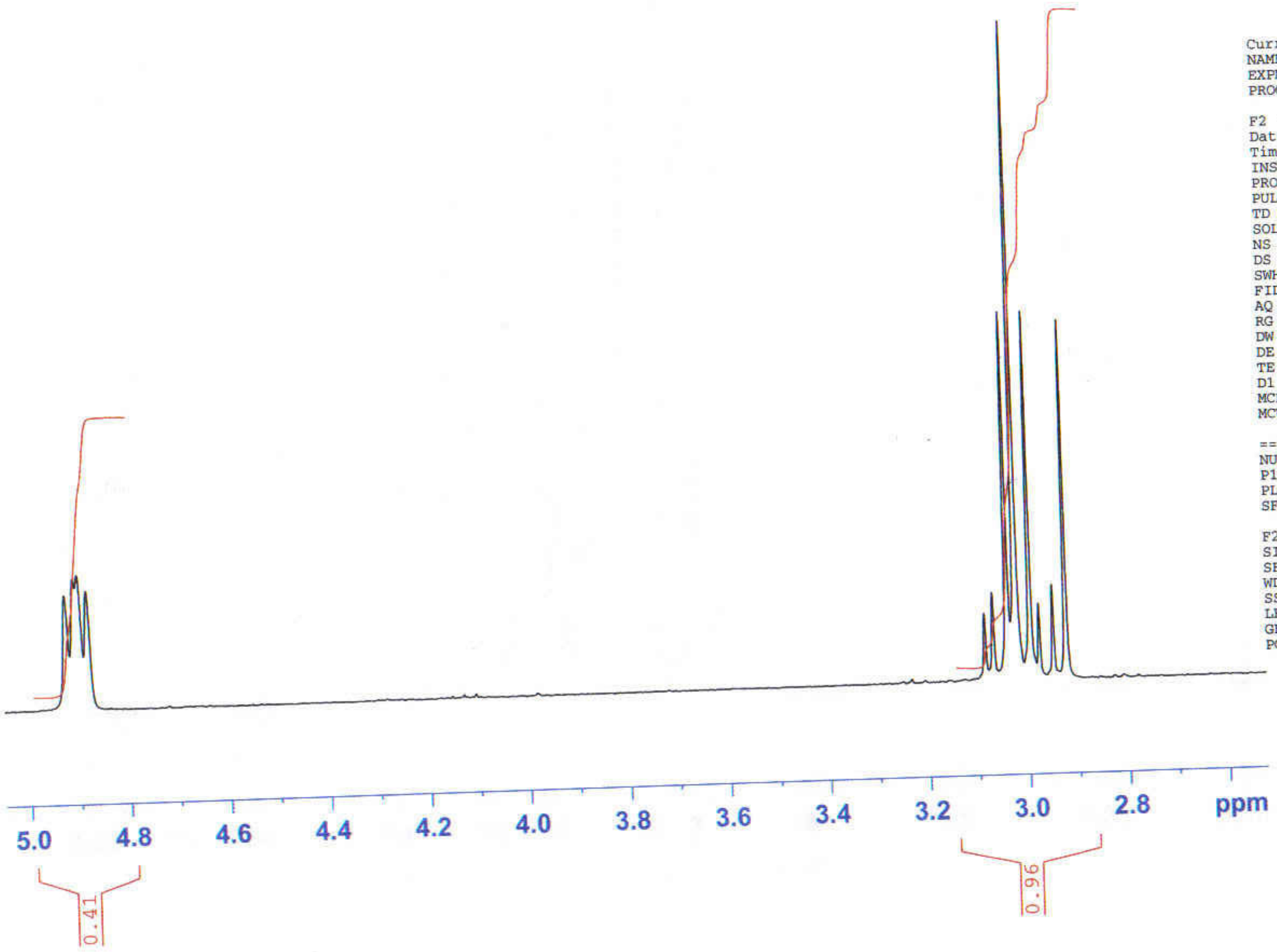

Current Data Parameters

NAME	10368288
EXPNO	10
PROCNO	1

F2 - Acquisition Parameters

Date_	20050526
Time	21.12
INSTRUM	dpx300
PROBHD	5 mm QNP 1H/1
PULPROG	zg30
TD	48848
SOLVENT	CDC13
NS	64
DS	2
SWH	7183.908 Hz
FIDRES	0.147067 Hz
AQ	3.3998709 sec
RG	724.1
DW	69.600 usec
DE	6.00 usec
TE	299.2 K
D1	6.59999990 sec
MCREST	0.00000000 sec
MCWRK	0.01500000 sec

======== CHANNEL f1 ========

NUC1	1H
P1	9.40 usec
PL1	0.00 dB
SFO1	300.1330010 MHz

F2 - Processing parameters

SI	65536
SF	300.1300079 MHz
WDW	EM
SSB	0
LB	0.30 Hz
GB	0
PC	2.00

D. [illegible]
27/5/05

10368288, NBZ5769/1/8, BRUKER DPX300, QNP, H1 SPECTRUM, SOLV:- CDCL3/TMS

Intertek ASG

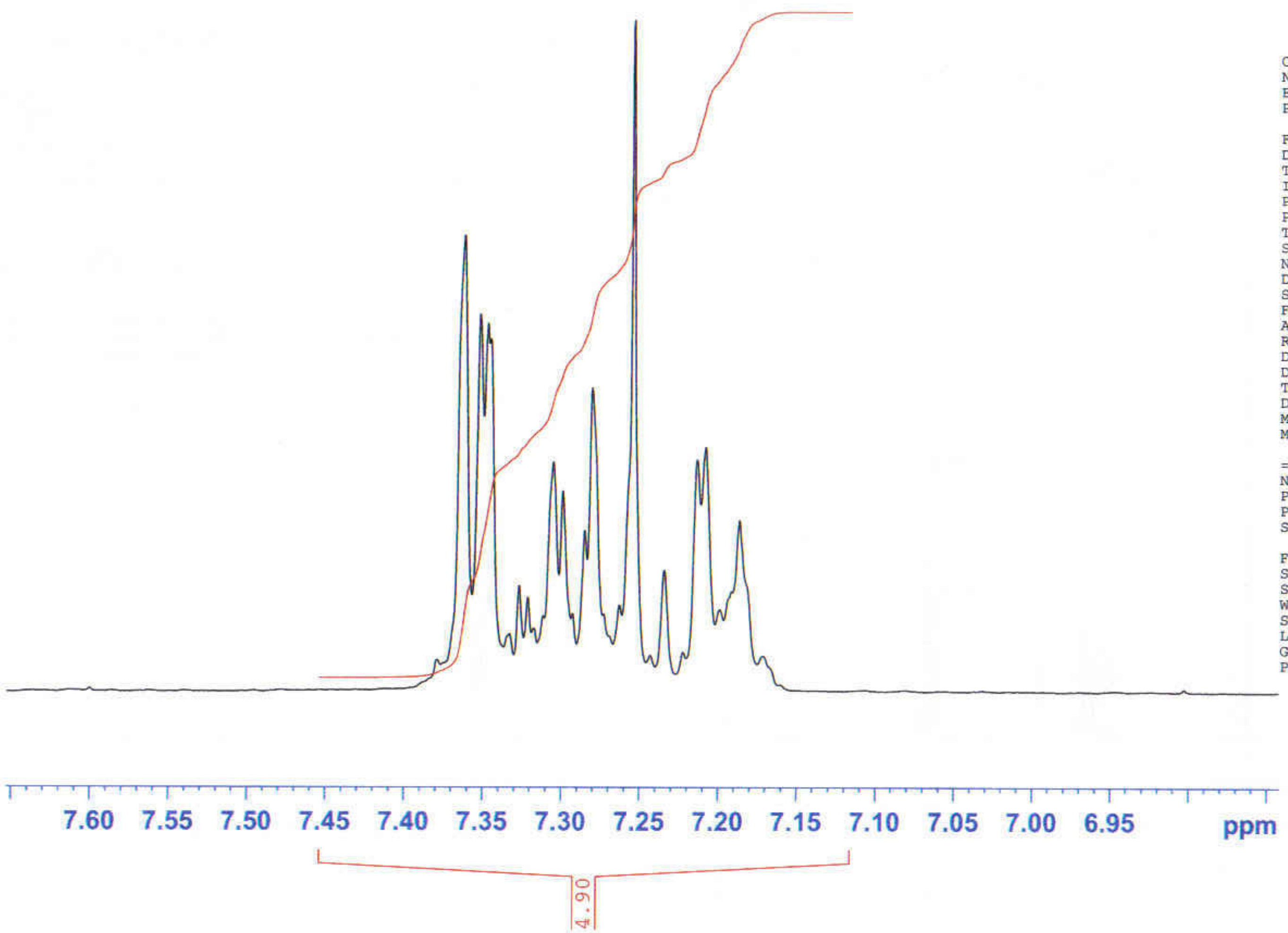

Current Data Parameters	
NAME	10368288
EXPNO	10
PROCNO	1
F2 - Acquisition Parameters	
Date_	20050526
Time	21.12
INSTRUM	dpx300
PROBHD	5 mm QNP 1H/1
PULPROG	zg30
TD	48848
SOLVENT	CDCl3
NS	64
DS	2
SWH	7183.908 Hz
FIDRES	0.147067 Hz
AQ	3.3998709 sec
RG	724.1
DW	69.600 usec
DE	6.00 usec
TE	299.2 K
D1	6.59999990 sec
MCREST	0.00000000 sec
MCWRK	0.01500000 sec
======== CHANNEL f1 ========	
NUC1	1H
P1	9.40 usec
PL1	0.00 dB
SFO1	300.1330010 MHz
F2 - Processing parameters	
SI	65536
SF	300.1300079 MHz
WDW	EM
SSB	0
LB	0.30 Hz
GB	0
PC	2.00

[illegible] 27/5/05

10368724, NBZ5769-9-4, BRUKER DPX300, QNP, H1 SPECTRUM, SOLV: CDCL3/TMS

Intertek ASG

Table 3 Entry 2

```
;zg30
;avance-version (02/05/31)
;1D sequence
;using 30 degree flip angle

#include <Avance.incl>

1 ze
2 30m
  d1
  p1*0.33 ph1
  go=2 ph31
  30m mc #0 to 2 F0(zd)
exit

ph1=0 2 2 0 1 3 3 1
ph31=0 2 2 0 1 3 3 1

;pl1 : f1 channel - power level for pulse (defau
;p1 : f1 channel -  90 degree high power pulse
;d1 : relaxation delay; 1-5 * T1
```

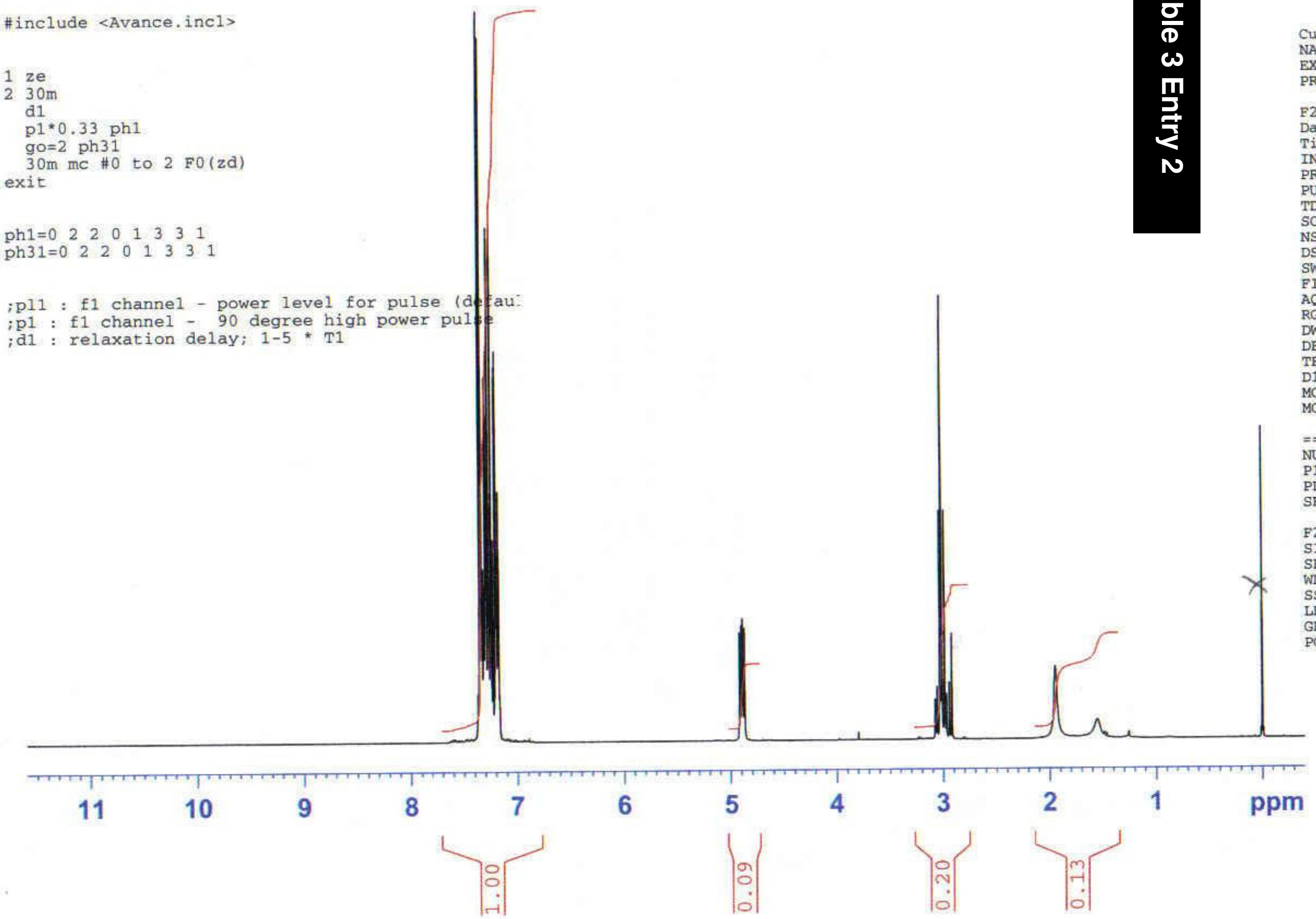


```
Current Data Parameters
NAME              10368724
EXPNO                   10
PROCNO                   1

F2 - Acquisition Parameters
Date_             20050609
Time                 15.04
INSTRUM             dpx300
PROBHD      5 mm QNP  1H/1
PULPROG               zg30
TD                   48848
SOLVENT              CDC13
NS                      64
DS                       2
SWH               7183.908 Hz
FIDRES            0.147067 Hz
AQ               3.3998709 sec
RG                   406.4
DW                  69.600 usec
DE                    6.00 usec
TE                   299.2 K
D1              6.59999990 sec
MCREST          0.00000000 sec
MCWRK           0.01500000 sec

======== CHANNEL f1 ========
NUC1                    1H
P1                    9.40 usec
PL1                   0.00 dB
SFO1           300.1330010 MHz

F2 - Processing parameters
SI                   65536
SF             300.1300101 MHz
WDW                     EM
SSB                      0
LB                    0.30 Hz
GB                       0
PC                    2.00
```

10/6/05

10368724, NBZ5769-9-4, BRUKER DPX300, QNP, H1 SPECTRUM, SOLV: CDCL3/TMS

Intertek ASG

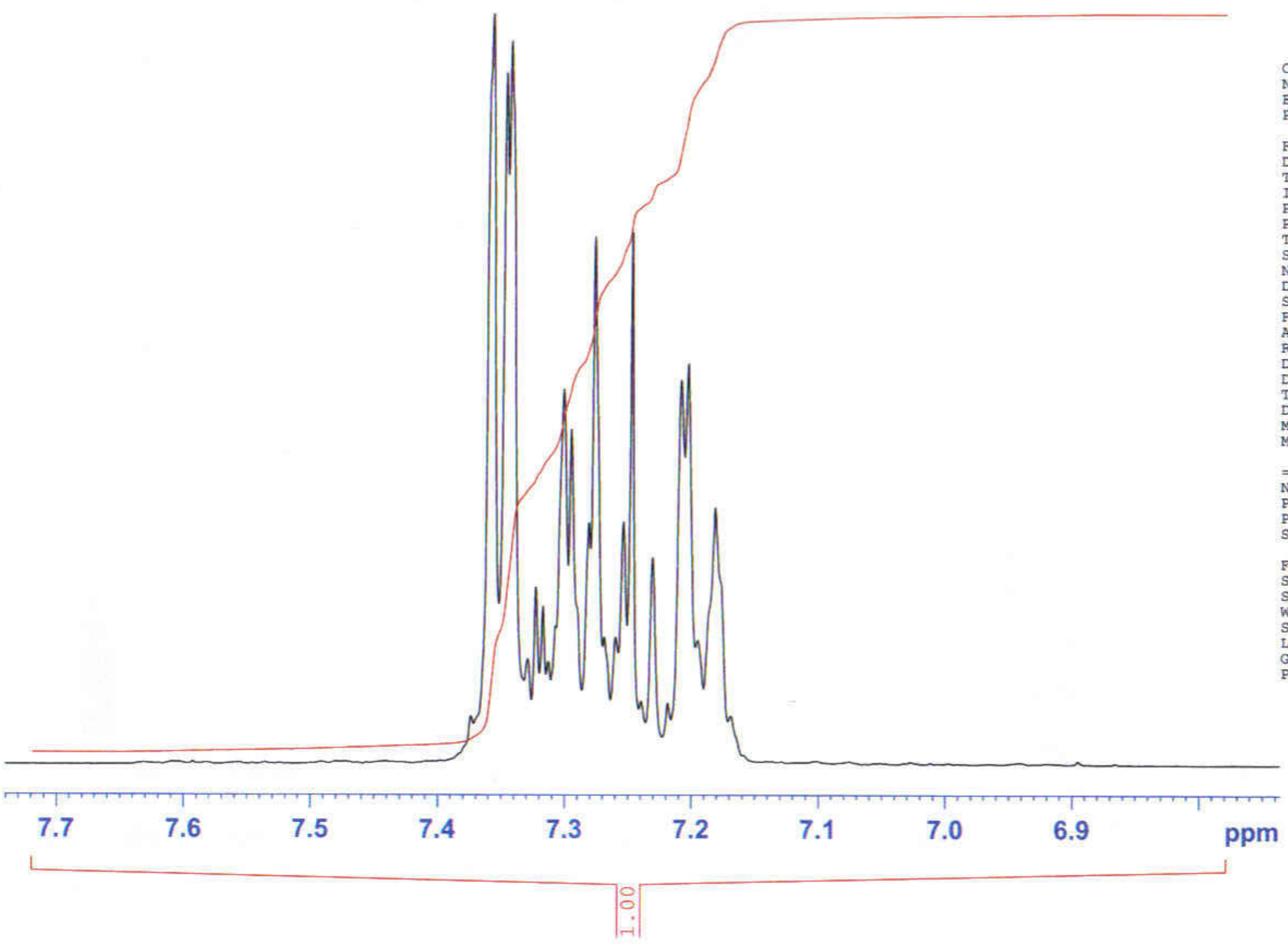

Current Data Parameters

NAME	10368724
EXPNO	10
PROCNO	1

F2 - Acquisition Parameters

Date_	20050609
Time	15.04
INSTRUM	dpx300
PROBHD	5 mm QNP 1H/1
PULPROG	zg30
TD	48848
SOLVENT	CDCl3
NS	64
DS	2
SWH	7183.908 Hz
FIDRES	0.147067 Hz
AQ	3.3998709 sec
RG	406.4
DW	69.600 usec
DE	6.00 usec
TE	299.2 K
D1	6.59999990 sec
MCREST	0.00000000 sec
MCWRK	0.01500000 sec

======== CHANNEL f1 ========

NUC1	1H
P1	9.40 usec
PL1	0.00 dB
SFO1	300.1330010 MHz

F2 - Processing parameters

SI	65536
SF	300.1300101 MHz
WDW	EM
SSB	0
LB	0.30 Hz
GB	0
PC	2.00

D. Meuf[illegible] 10/6/05

10368724, NBZ5769-9-4, BRUKER DPX300, QNP, H1 SPECTRUM, SOLV: CDCL3/TMS

Intertek ASG

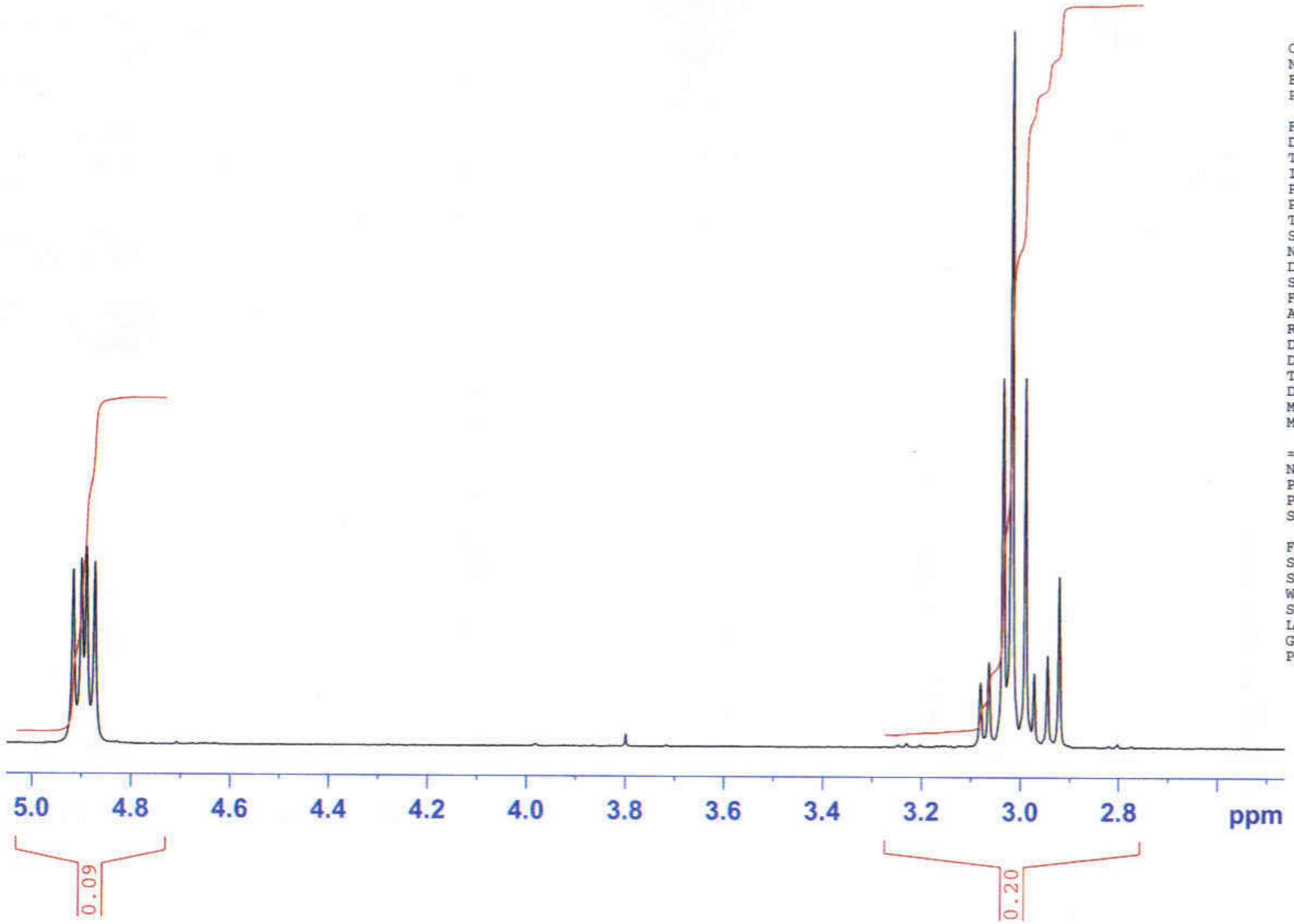


```
Current Data Parameters
NAME             10368724
EXPNO                  10
PROCNO                  1

F2 - Acquisition Parameters
Date_            20050609
Time                15.04
INSTRUM            dpx300
PROBHD    5 mm QNP  1H/1
PULPROG              zg30
TD                  48848
SOLVENT             CDC13
NS                     64
DS                      2
SWH              7183.908 Hz
FIDRES           0.147067 Hz
AQ              3.3998709 sec
RG                  406.4
DW                 69.600 usec
DE                   6.00 usec
TE                  299.2 K
D1             6.59999990 sec
MCREST         0.00000000 sec
MCWRK          0.01500000 sec

======== CHANNEL f1 ========
NUC1                   1H
P1                   9.40 usec
PL1                  0.00 dB
SFO1          300.1330010 MHz

F2 - Processing parameters
SI                  65536
SF            300.1300101 MHz
WDW                    EM
SSB                     0
LB                   0.30 Hz
GB                      0
PC                   2.00
```

D. Nutt
10/6/05

10368725, NBZ5769-9-5, BRUKER DPX300, QNP, H1 SPECTRUM, SOLV: CDCL3/TMS

Intertek ASG

```
;zg30
;avance-version (02/05/31)
;1D sequence
;using 30 degree flip angle

#include <Avance.incl>

1 ze
2 30m
  d1
  p1*0.33 ph1
  go=2 ph31
  30m mc #0 to 2 F0(zd)
exit

ph1=0 2 2 0 1 3 3 1
ph31=0 2 2 0 1 3 3 1

;pl1 : f1 channel - power level for pulse (defau
;p1 : f1 channel -  90 degree high power pulse
;d1 : relaxation delay; 1-5 * T1
```

Table 3 Entry 3

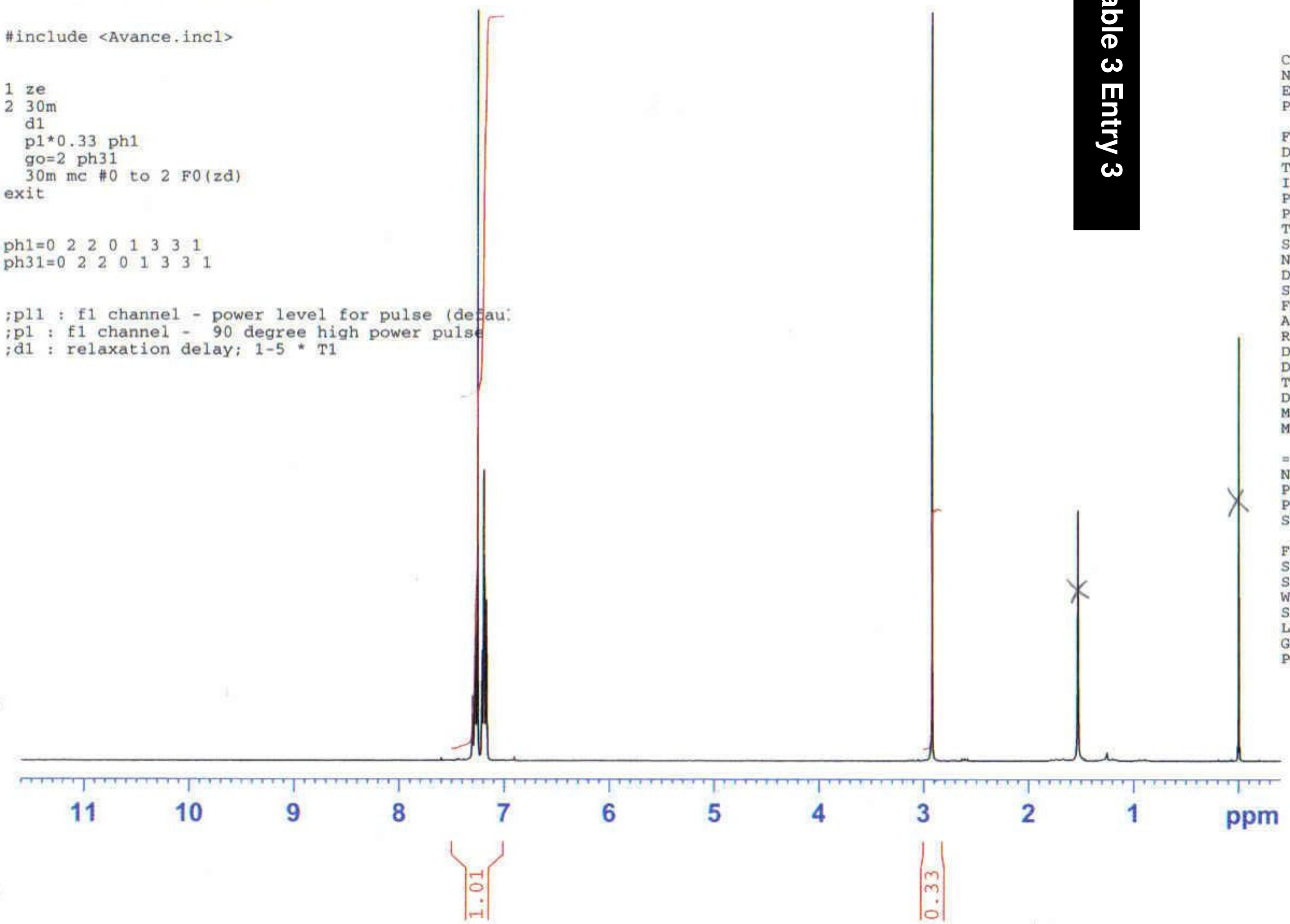


```
Current Data Parameters
NAME              10368725
EXPNO                   10
PROCNO                   1

F2 - Acquisition Parameters
Date_             20050609
Time                 15.21
INSTRUM             dpx300
PROBHD     5 mm QNP  1H/1
PULPROG               zg30
TD                   48848
SOLVENT              CDCl3
NS                      64
DS                       2
SWH               7183.908 Hz
FIDRES            0.147067 Hz
AQ               3.3998709 sec
RG                  1290.2
DW                  69.600 usec
DE                    6.00 usec
TE                   299.2 K
D1              6.59999990 sec
MCREST          0.00000000 sec
MCWRK           0.01500000 sec

======== CHANNEL f1 ========
NUC1                    1H
P1                    9.40 usec
PL1                   0.00 dB
SFO1           300.1330010 MHz

F2 - Processing parameters
SI                   65536
SF             300.1300074 MHz
WDW                     EM
SSB                      0
LB                    0.30 Hz
GB                       0
PC                    2.00
```

D. Nalt
10/6/05

10368725, NBZ5769-9-5, BRUKER DPX300, QNP, H1 SPECTRUM, SOLV: CDCL3/TMS

Intertek ASG

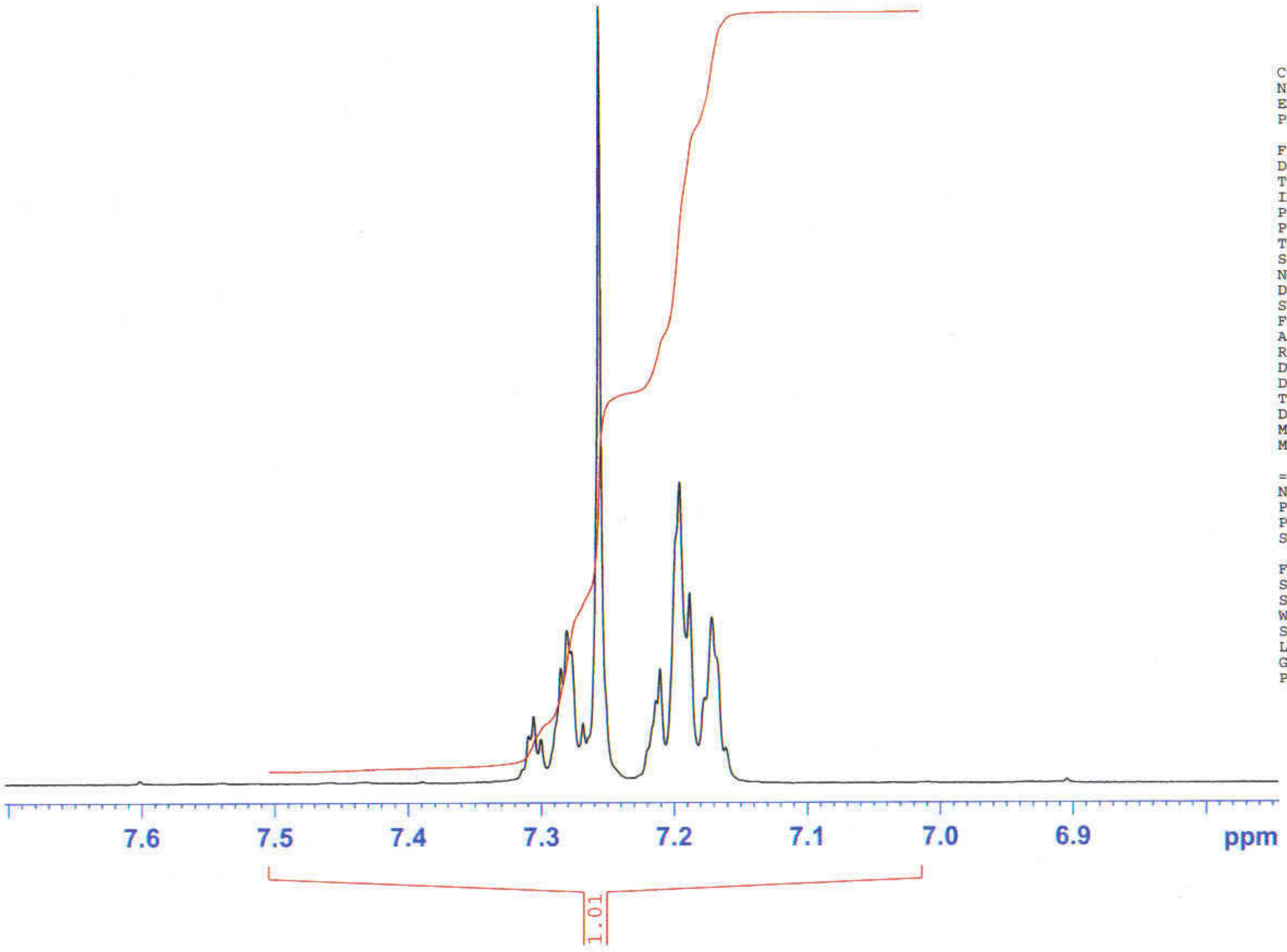


```
Current Data Parameters
NAME          10368725
EXPNO               10
PROCNO               1

F2 - Acquisition Parameters
Date_         20050609
Time             15.21
INSTRUM         dpx300
PROBHD   5 mm QNP 1H/1
PULPROG           zg30
TD               48848
SOLVENT          CDCl3
NS                  64
DS                   2
SWH           7183.908 Hz
FIDRES        0.147067 Hz
AQ           3.3998709 sec
RG              1290.2
DW              69.600 usec
DE                6.00 usec
TE               299.2 K
D1          6.59999990 sec
MCREST      0.00000000 sec
MCWRK       0.01500000 sec

======== CHANNEL f1 ========
NUC1                1H
P1                9.40 usec
PL1               0.00 dB
SFO1       300.1330010 MHz

F2 - Processing parameters
SI               65536
SF         300.1300074 MHz
WDW                 EM
SSB                  0
LB                0.30 Hz
GB                   0
PC                2.00
```

D. Albert
10/6/05

10368961, NBZ5769-11-1, BRUKER DPX300, QNP, H1 SPECTRUM, SOLV:-CDCL3/TMS

Intertek ASG

Table 3 Entry 4

```
;zg30
;avance-version (02/05/31)
;1D sequence
;using 30 degree flip angle

#include <Avance.incl>

1 ze
2 30m
  d1
  p1*0.33 ph1
  go=2 ph31
  30m mc #0 to 2 F0(zd)
exit

ph1=0 2 2 0 1 3 3 1
ph31=0 2 2 0 1 3 3 1

;pl1 : f1 channel - power level for pulse (defau
;p1 : f1 channel -  90 degree high power pulse
;d1 : relaxation delay; 1-5 * T1
```

```
Current Data Parameters
NAME            10368961
EXPNO                 10
PROCNO                 1

F2 - Acquisition Parameters
Date_           20050615
Time               12.27
INSTRUM           dpx300
PROBHD    5 mm QNP  1H/1
PULPROG             zg30
TD                 48848
SOLVENT            CDCl3
NS                    64
DS                     2
SWH             7183.908 Hz
FIDRES          0.147067 Hz
AQ             3.3998709 sec
RG                1290.2
DW                69.600 usec
DE                  6.00 usec
TE                 299.2 K
D1            6.59999990 sec
MCREST        0.00000000 sec
MCWRK         0.01500000 sec

======== CHANNEL f1 ========
NUC1                  1H
P1                  9.40 usec
PL1                 0.00 dB
SFO1         300.1330010 MHz

F2 - Processing parameters
SI                 65536
SF           300.1300068 MHz
WDW                   EM
SSB                    0
LB                  0.30 Hz
GB                     0
PC                  2.00
```

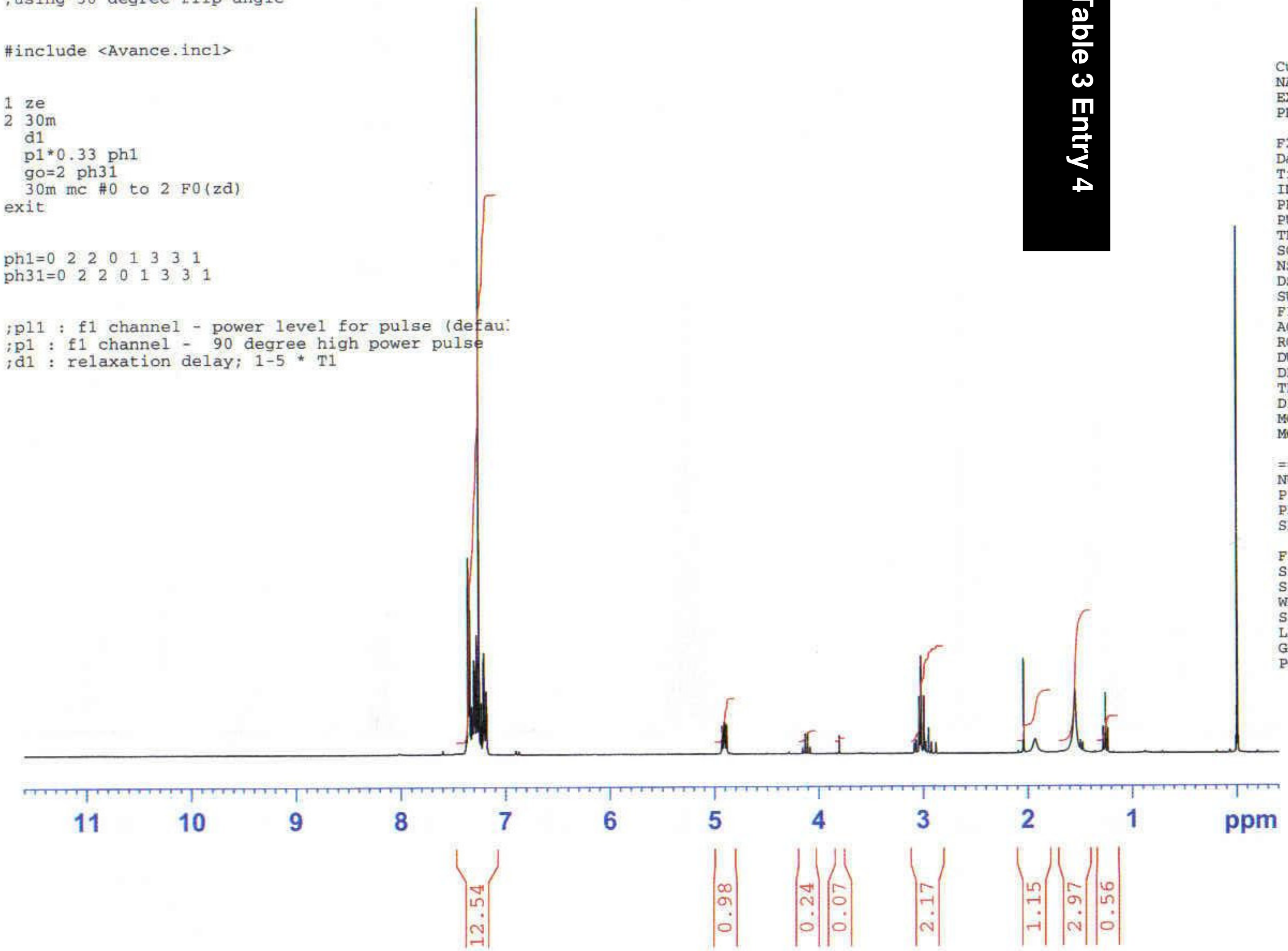

D. Mutt
16/6/05

10368961, NBZ5769-11-1, BRUKER DPX300, QNP, H1 SPECTRUM, SOLV:-CDCL3/TMS

Intertek ASG

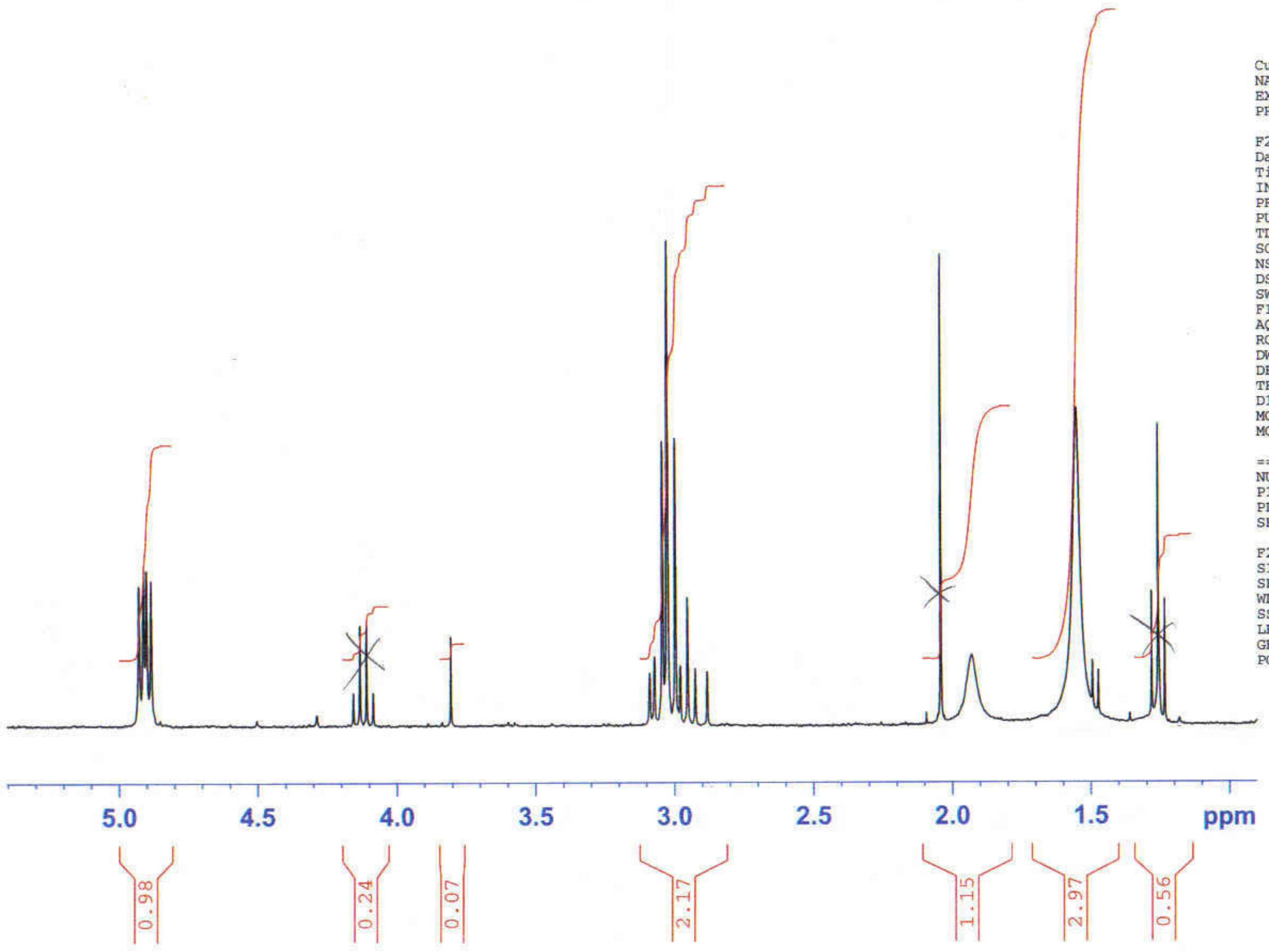


```
Current Data Parameters
NAME             10368961
EXPNO                  10
PROCNO                  1

F2 - Acquisition Parameters
Date_            20050615
Time                12.27
INSTRUM            dpx300
PROBHD    5 mm QNP  1H/1
PULPROG              zg30
TD                  48848
SOLVENT             CDCl3
NS                     64
DS                      2
SWH              7183.908 Hz
FIDRES           0.147067 Hz
AQ              3.3998709 sec
RG                 1290.2
DW                 69.600 usec
DE                   6.00 usec
TE                  299.2 K
D1             6.59999990 sec
MCREST         0.00000000 sec
MCWRK          0.01500000 sec

======== CHANNEL f1 ========
NUC1                   1H
P1                   9.40 usec
PL1                  0.00 dB
SFO1          300.1330010 MHz

F2 - Processing parameters
SI                  65536
SF            300.1300068 MHz
WDW                    EM
SSB                     0
LB                   0.30 Hz
GB                      0
PC                   2.00
```

10368961, NBZ5769-11-1, BRUKER DPX300, QNP, H1 SPECTRUM, SOLV:-CDCL3/TMS

Intertek ASG

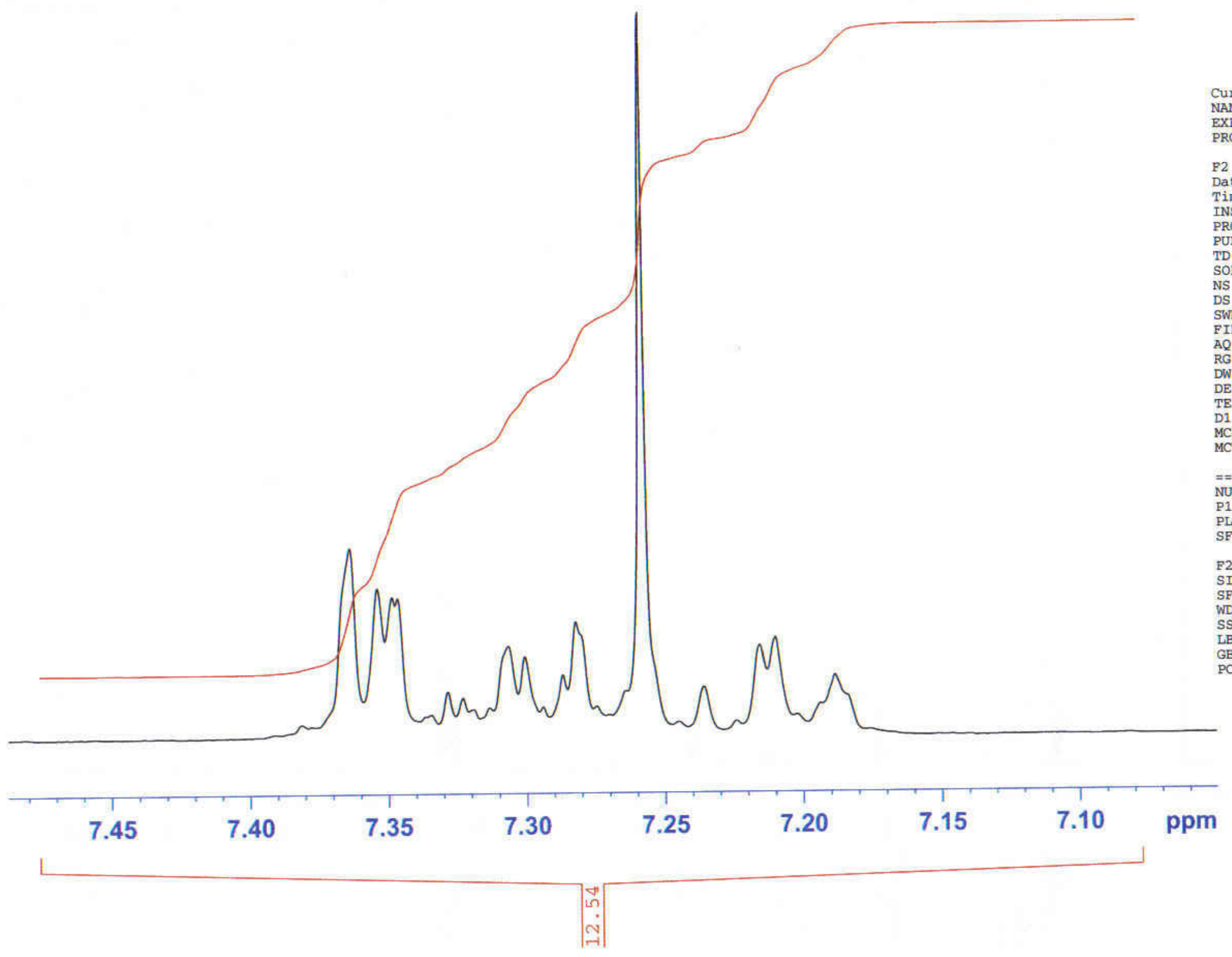


```
Current Data Parameters
NAME          10368961
EXPNO               10
PROCNO               1

F2 - Acquisition Parameters
Date_         20050615
Time             12.27
INSTRUM         dpx300
PROBHD   5 mm QNP 1H/1
PULPROG           zg30
TD               48848
SOLVENT          CDCl3
NS                  64
DS                   2
SWH           7183.908 Hz
FIDRES        0.147067 Hz
AQ           3.3998709 sec
RG              1290.2
DW              69.600 usec
DE                6.00 usec
TE               299.2 K
D1          6.59999990 sec
MCREST      0.00000000 sec
MCWRK       0.01500000 sec

======== CHANNEL f1 ========
NUC1                1H
P1                9.40 usec
PL1               0.00 dB
SFO1       300.1330010 MHz

F2 - Processing parameters
SI               65536
SF         300.1300068 MHz
WDW                 EM
SSB                  0
LB                0.30 Hz
GB                   0
PC                2.00
```

D. Mut[illegible]
16/6/05

10383689, RX0002/44A, BRUKER DPX300, QNP, H1 SPECTRUM, SOLV:-CDCL3/TMS

TABLE 4 ENTRY 1

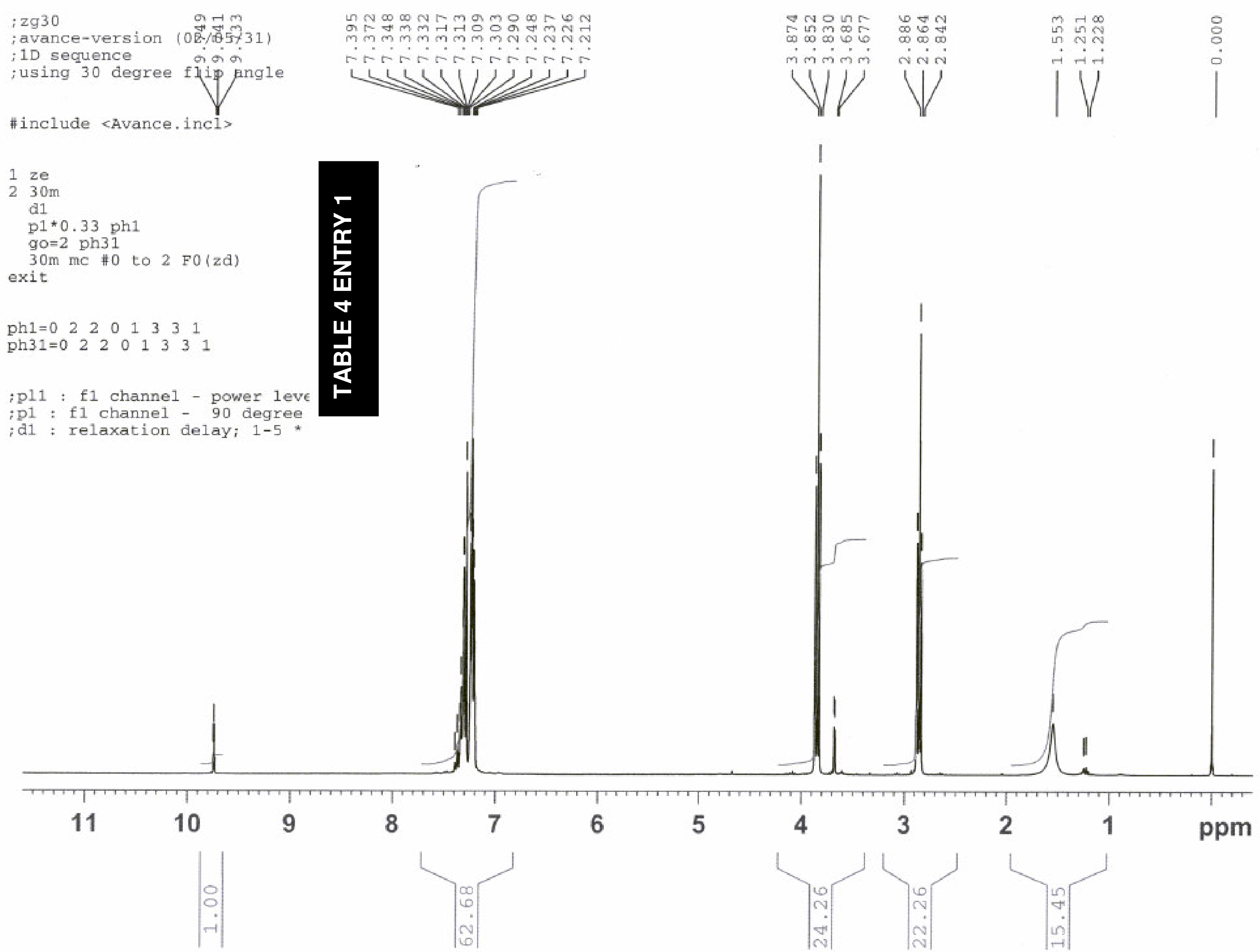

Intertek ASG

Current Data Parameters	
NAME	10383689
EXPNO	10
PROCNO	1
F2 - Acquisition Parameters	
Date_	20060719
Time	12.00
INSTRUM	dpx300
PROBHD	5 mm QNP 1H/13
PULPROG	zg30
TD	48848
SOLVENT	CDCl3
NS	64
DS	2
SWH	7183.908 Hz
FIDRES	0.147067 Hz
AQ	3.3998709 sec
RG	322.5
DW	69.600 usec
DE	6.00 usec
TE	299.2 K
D1	6.59999990 sec
MCREST	0.00000000 sec
MCWRK	0.01500000 sec
======== CHANNEL f1 ========	
NUC1	1H
P1	9.40 usec
PL1	0.00 dB
SFO1	300.1330010 MHz
F2 - Processing parameters	
SI	65536
SF	300.1300095 MHz
WDW	EM
SSB	0
LB	0.30 Hz
GB	0
PC	20.00

19/7/06

10383689, RX0002/44A, BRUKER DPX300, QNP, H1 SPECTRUM, SOLV:-CDCL3/TMS

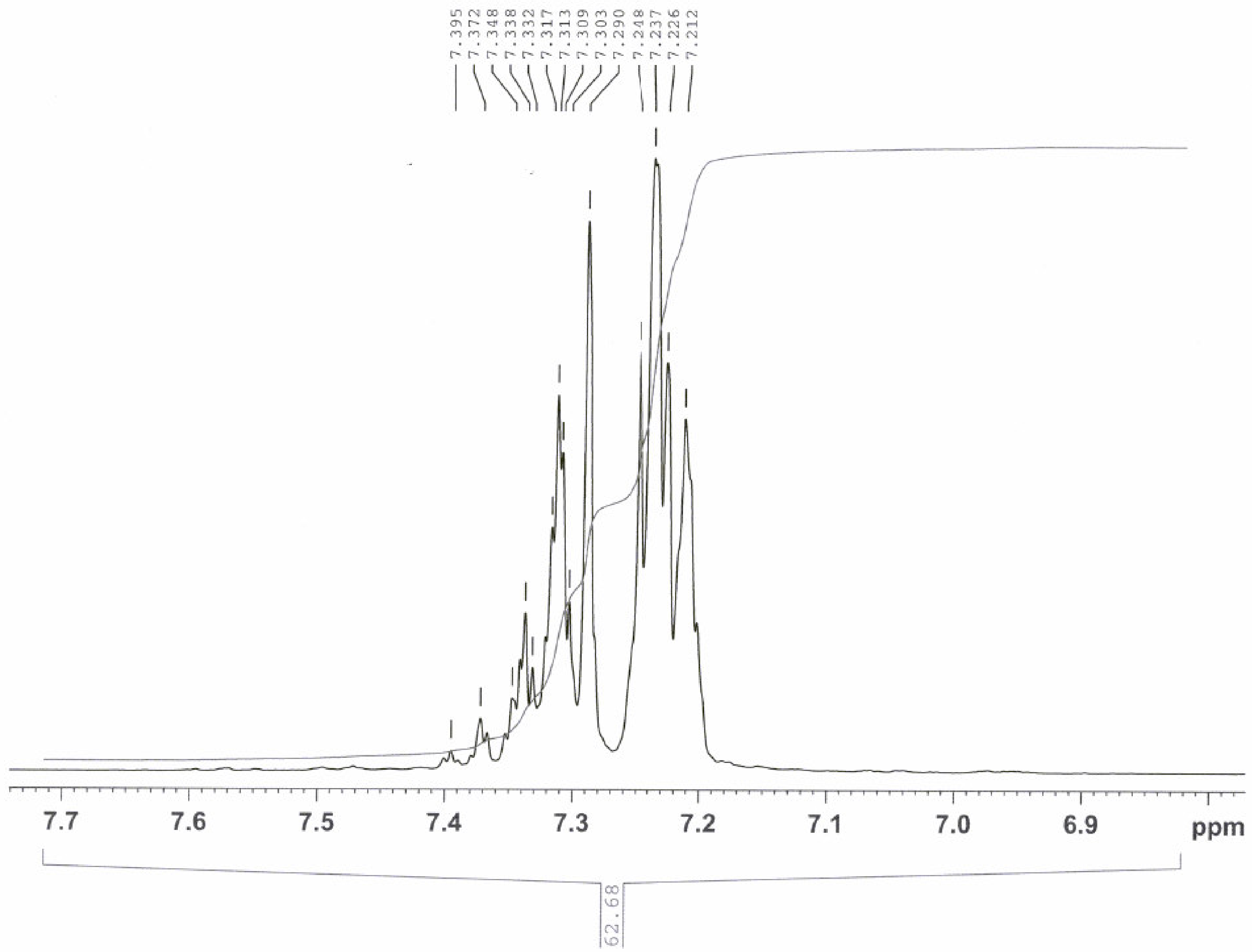

Intertek ASG

Current Data Parameters

NAME	10383689
EXPNO	10
PROCNO	1

F2 - Acquisition Parameters

Date_	20060719
Time	12.00
INSTRUM	dpx300
PROBHD	5 mm QNP 1H/13
PULPROG	zg30
TD	48848
SOLVENT	CDCl3
NS	64
DS	2
SWH	7183.908 Hz
FIDRES	0.147067 Hz
AQ	3.3998709 sec
RG	322.5
DW	69.600 usec
DE	6.00 usec
TE	299.2 K
D1	6.59999990 sec
MCREST	0.00000000 sec
MCWRK	0.01500000 sec

======== CHANNEL f1 ========

NUC1	1H
P1	9.40 usec
PL1	0.00 dB
SFO1	300.1330010 MHz

F2 - Processing parameters

SI	65536
SF	300.1300095 MHz
WDW	EM
SSB	0
LB	0.30 Hz
GB	0
PC	20.00

D. [illegible]
19/7/06

10383689, RX0002/44A, BRUKER DPX300, QNP, H1 SPECTRUM, SOLV:-CDCL3/TMS

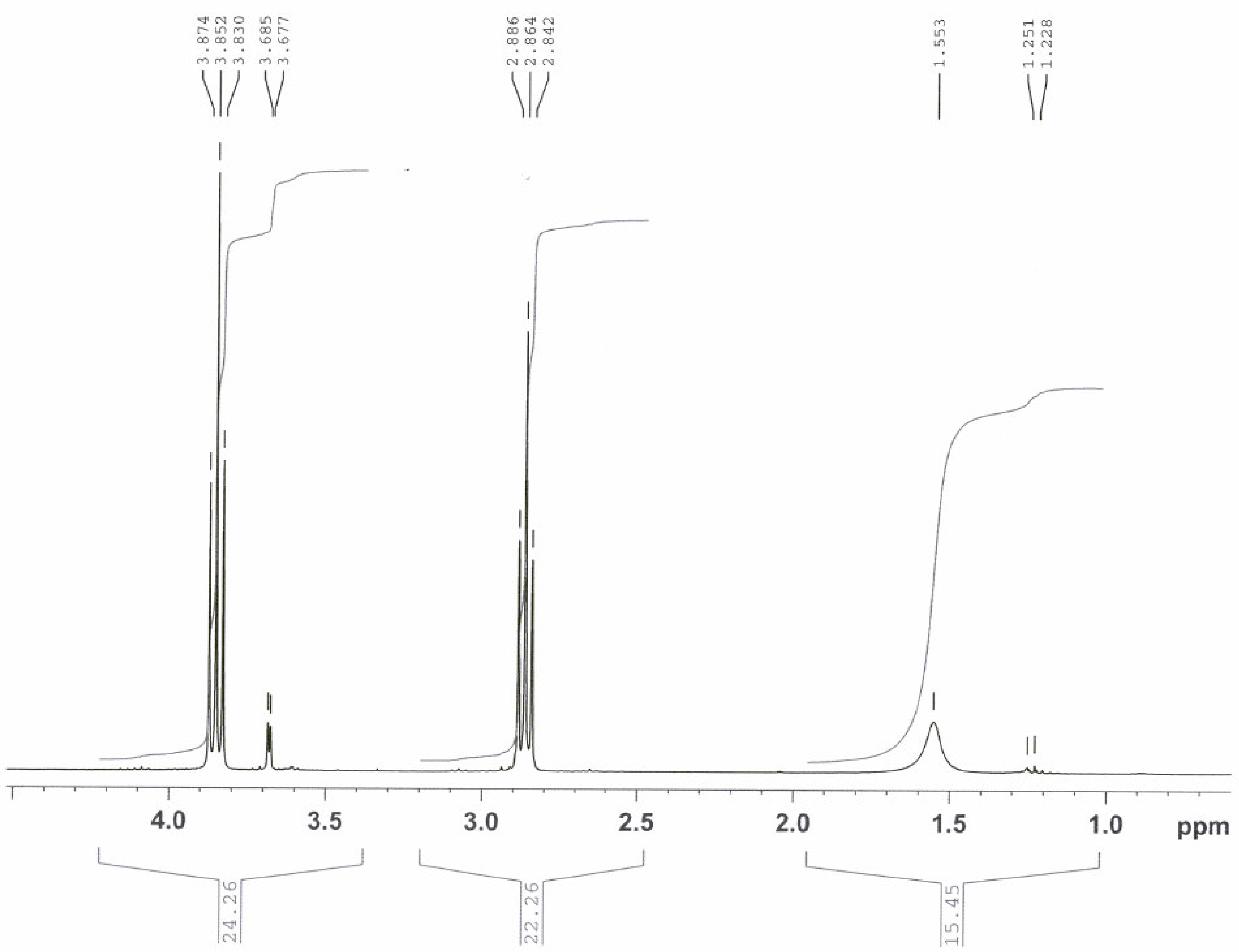

Intertek ASG

Current Data Parameters	
NAME	10383689
EXPNO	10
PROCNO	1
F2 - Acquisition Parameters	
Date_	20060719
Time	12.00
INSTRUM	dpx300
PROBHD	5 mm QNP 1H/13
PULPROG	zg30
TD	48848
SOLVENT	CDCl3
NS	64
DS	2
SWH	7183.908 Hz
FIDRES	0.147067 Hz
AQ	3.3998709 sec
RG	322.5
DW	69.600 usec
DE	6.00 usec
TE	299.2 K
D1	6.59999990 sec
MCREST	0.00000000 sec
MCWRK	0.01500000 sec
======== CHANNEL f1 ========	
NUC1	1H
P1	9.40 usec
PL1	0.00 dB
SFO1	300.1330010 MHz
F2 - Processing parameters	
SI	65536
SF	300.1300095 MHz
WDW	EM
SSB	0
LB	0.30 Hz
GB	0
PC	20.00

D. [illegible]
19/7/06

10383690, RX0002/44B, BRUKER DPX300, QNP, H1 SPECTRUM, SOLV:-CDCL3/TMS

```
;zg30
;avance-version (02/05/31)
;1D sequence
;using 30 degree flip angle

#include <Avance.incl>

1 ze
2 30m
  d1
  p1*0.33 ph1
  go=2 ph31
  30m mc #0 to 2 F0(zd)
exit

ph1=0 2 2 0 1 3 3 1
ph31=0 2 2 0 1 3 3 1

;pl1 : f1 channel - power leve
;p1 : f1 channel -  90 degree
;d1 : relaxation delay; 1-5 *
```

TABLE 4 ENTRY 2

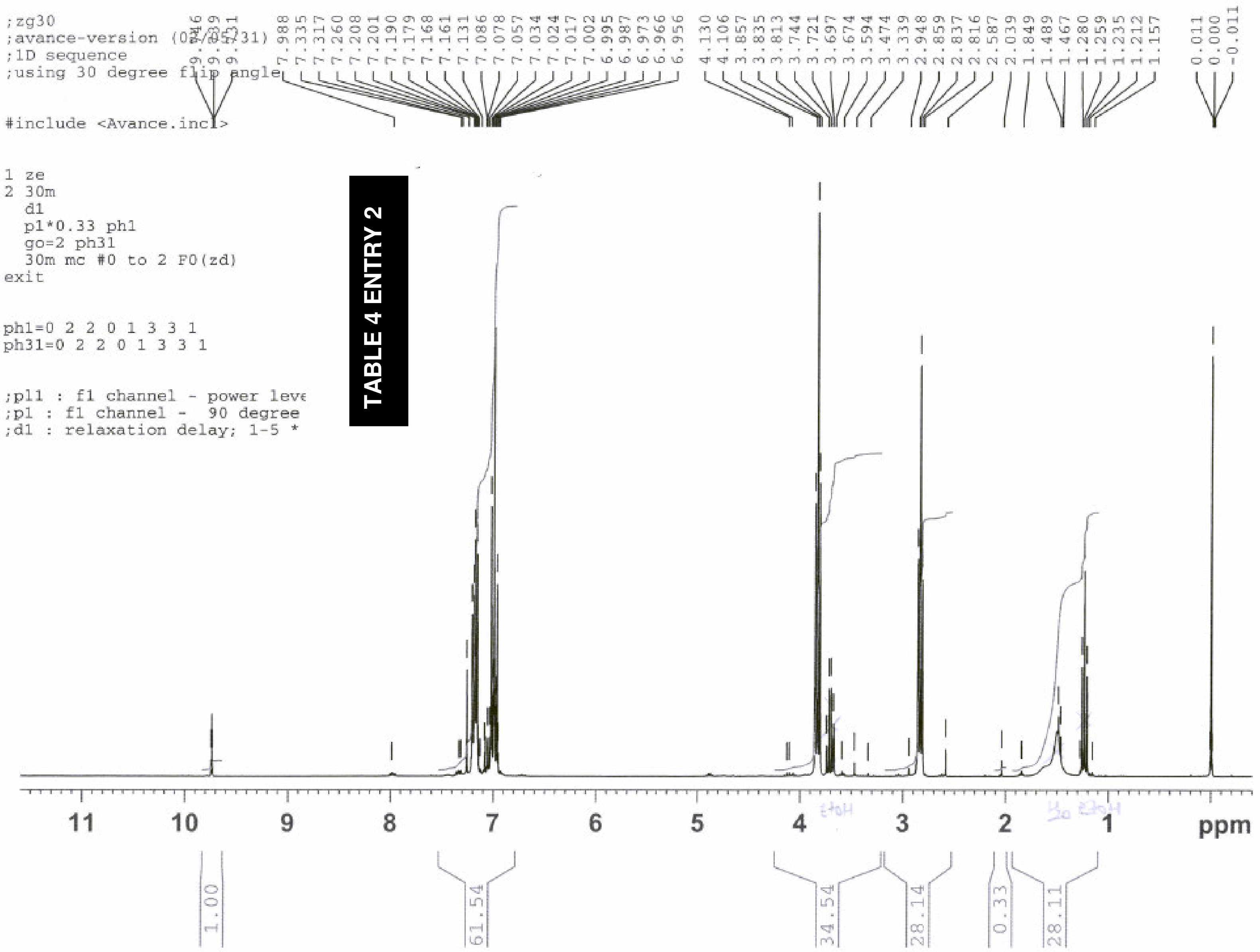

Intertek ASG

Current Data Parameters	
NAME	10383690
EXPNO	10
PROCNO	1

F2 - Acquisition Parameters	
Date_	20060719
Time	12.18
INSTRUM	dpx300
PROBHD	5 mm QNP 1H/13
PULPROG	zg30
TD	48848
SOLVENT	CDCl3
NS	64
DS	2
SWH	7183.908 Hz
FIDRES	0.147067 Hz
AQ	3.3998709 sec
RG	322.5
DW	69.600 usec
DE	6.00 usec
TE	299.2 K
D1	6.59999990 sec
MCREST	0.00000000 sec
MCWRK	0.01500000 sec

======== CHANNEL f1 ========	
NUC1	1H
P1	9.40 usec
PL1	0.00 dB
SFO1	300.1330010 MHz

F2 - Processing parameters	
SI	65536
SF	300.1300062 MHz
WDW	EM
SSB	0
LB	0.30 Hz
GB	0
PC	2.00

19/7/06

10383690, RX0002/44B, BRUKER DPX300, QNP, H1 SPECTRUM, SOLV:-CDCL3/TMS

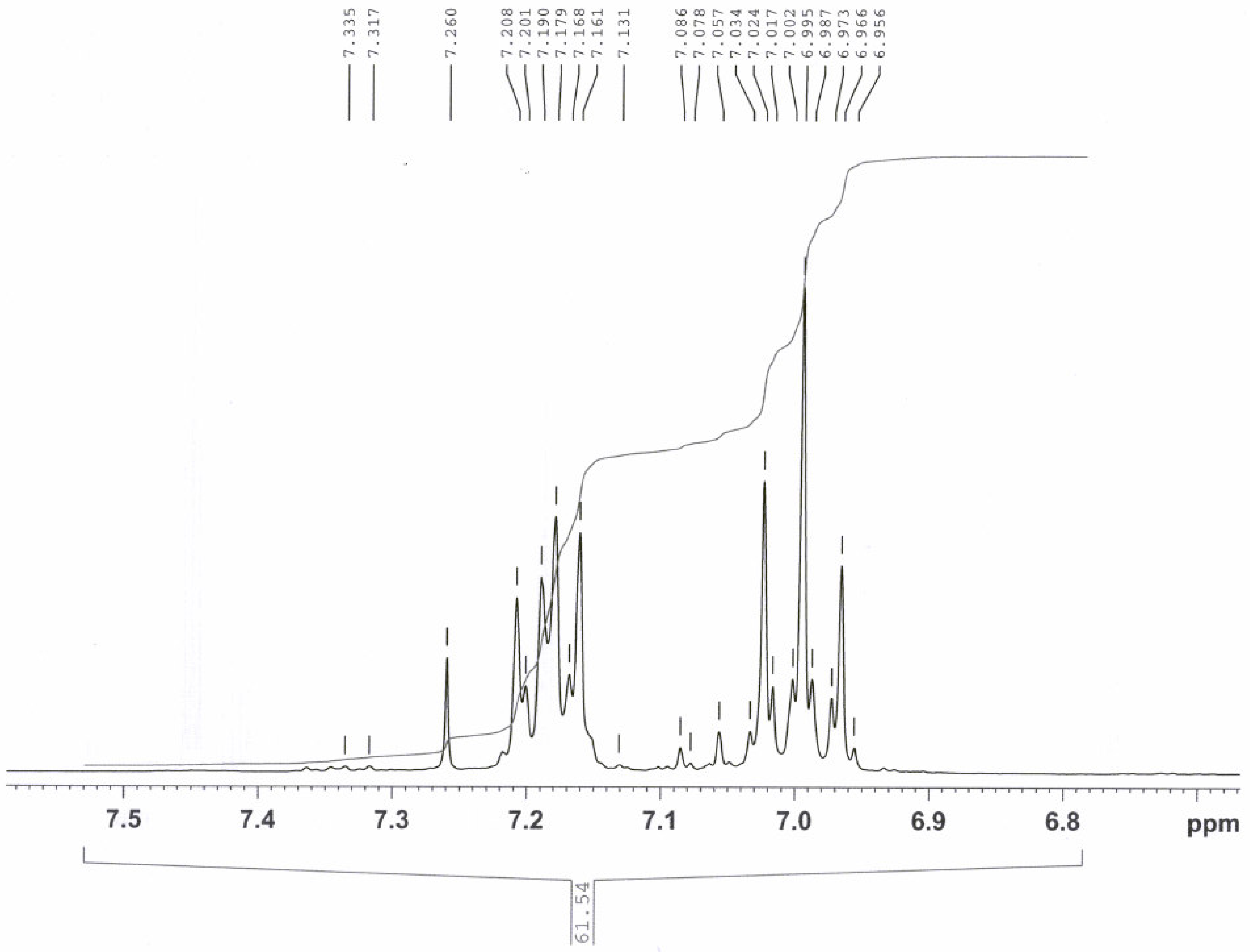

Intertek ASG

Current Data Parameters	
NAME	10383690
EXPNO	10
PROCNO	1

F2 - Acquisition Parameters	
Date_	20060719
Time	12.18
INSTRUM	dpx300
PROBHD	5 mm QNP 1H/13
PULPROG	zg30
TD	48848
SOLVENT	CDCl3
NS	64
DS	2
SWH	7183.908 Hz
FIDRES	0.147067 Hz
AQ	3.3998709 sec
RG	322.5
DW	69.600 usec
DE	6.00 usec
TE	299.2 K
D1	6.59999990 sec
MCREST	0.00000000 sec
MCWRK	0.01500000 sec

======== CHANNEL f1 ========	
NUC1	1H
P1	9.40 usec
PL1	0.00 dB
SFO1	300.1330010 MHz

F2 - Processing parameters	
SI	65536
SF	300.1300062 MHz
WDW	EM
SSB	0
LB	0.30 Hz
GB	0
PC	2.00

D. [illegible] 19/7/06

10383690, RX0002/44B, BRUKER DPX300, QNP, H1 SPECTRUM, SOLV:-CDCL3/TMS

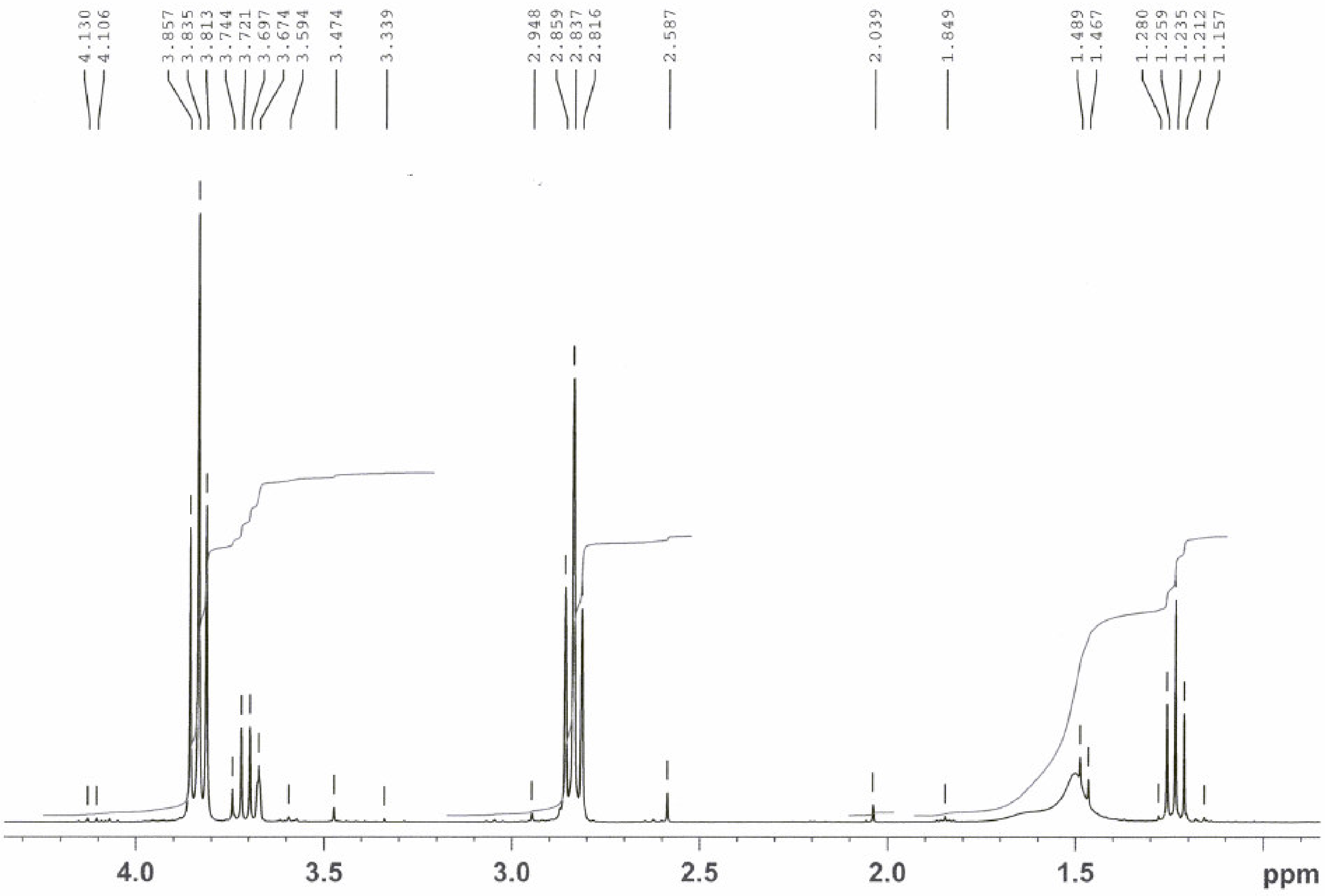

Intertek ASG

```
Current Data Parameters
NAME             10383690
EXPNO                  10
PROCNO                  1

F2 - Acquisition Parameters
Date_            20060719
Time                12.18
INSTRUM            dpx300
PROBHD   5 mm QNP 1H/13
PULPROG              zg30
TD                  48848
SOLVENT             CDC13
NS                     64
DS                      2
SWH              7183.908 Hz
FIDRES           0.147067 Hz
AQ              3.3998709 sec
RG                  322.5
DW                 69.600 usec
DE                   6.00 usec
TE                  299.2 K
D1             6.59999990 sec
MCREST         0.00000000 sec
MCWRK          0.01500000 sec

======== CHANNEL f1 ========
NUC1                   1H
P1                   9.40 usec
PL1                  0.00 dB
SFO1          300.1330010 MHz

F2 - Processing parameters
SI                  65536
SF            300.1300062 MHz
WDW                    EM
SSB                     0
LB                   0.30 Hz
GB                      0
PC                   2.00
```

10383450, RX0002/38/3, BRUKER DPX300, QNP, H1 SPECTRUM, SOLV:-CDCL3/TMS

```
;zg30
;avance-version (02/05/31)
;1D sequence
;using 30 degree flip angle

#include <Avance.incl>

1 ze
2 30m
  d1
  p1*0.33 ph1
  go=2 ph31
  30m mc #0 to 2 F0(zd)
exit

ph1=0 2 2 0 1 3 3 1
ph31=0 2 2 0 1 3 3 1

;pl1 : f1 channel - power leve
;p1  : f1 channel -  90 degree
;d1  : relaxation delay; 1-5 *
```

TABLE 4 ENTRY 3

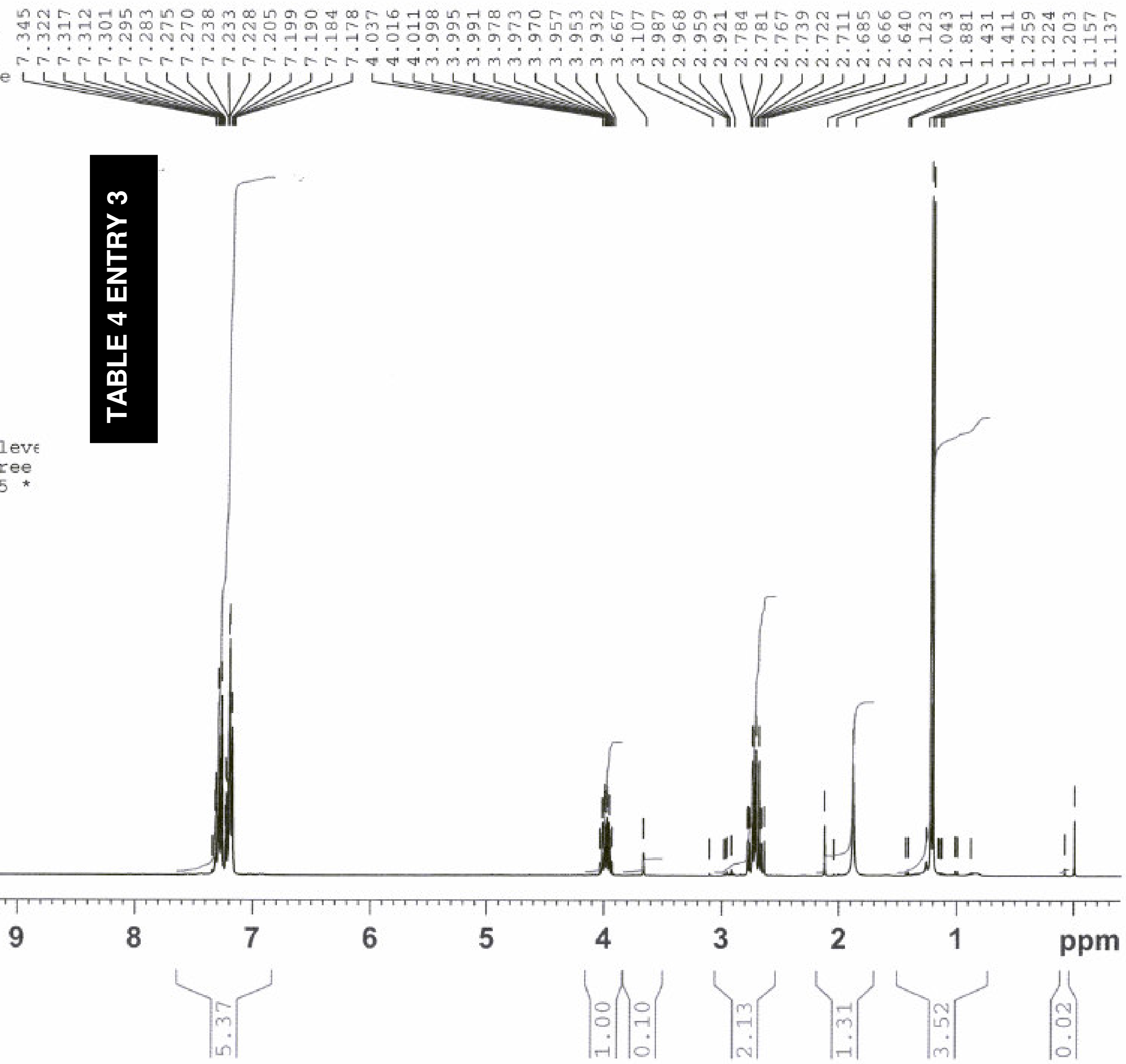

Intertek ASG

```
Current Data Parameters
NAME            10383450
EXPNO                 10
PROCNO                 1

F2 - Acquisition Parameters
Date_           20060712
Time               12.45
INSTRUM           dpx300
PROBHD   5 mm QNP 1H/13
PULPROG             zg30
TD                 48848
SOLVENT            CDCl3
NS                    64
DS                     2
SWH             7183.908 Hz
FIDRES          0.147067 Hz
AQ             3.3998709 sec
RG                    64
DW                69.600 usec
DE                  6.00 usec
TE                 299.2 K
D1            6.59999990 sec
MCREST        0.00000000 sec
MCWRK         0.01500000 sec

======== CHANNEL f1 ========
NUC1                  1H
P1                  9.40 usec
PL1                 0.00 dB
SFO1         300.1330010 MHz

F2 - Processing parameters
SI                 65536
SF           300.1300146 MHz
WDW                   EM
SSB                    0
LB                  0.30 Hz
GB                     0
PC                  2.00
```

D. Nold

10383450, RX0002/38/3, BRUKER DPX30D, QNP, H1 SPECTRUM, SOLV:-CDCL3/TMS

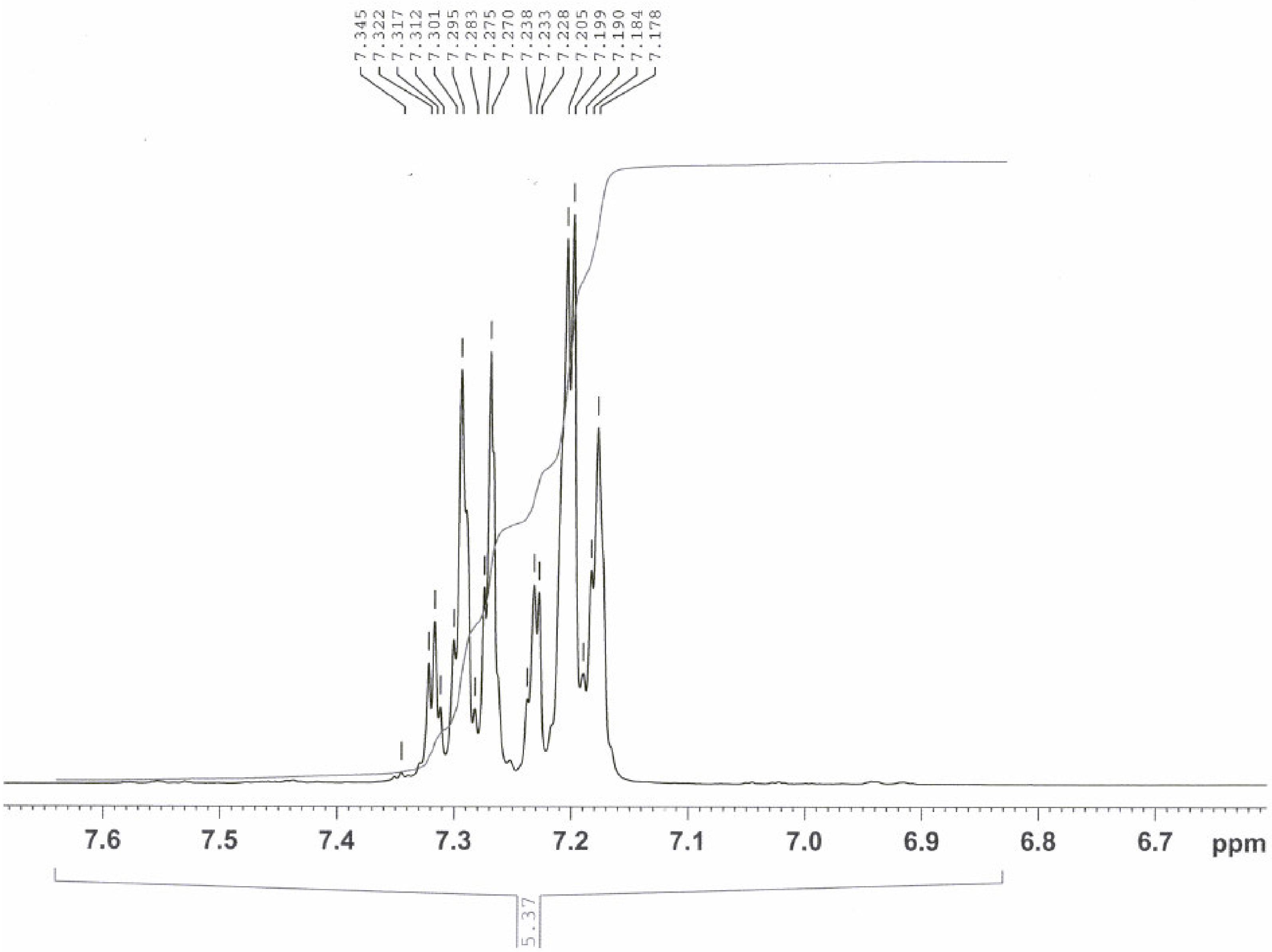

Intertek ASG

Current Data Parameters

NAME	10383450
EXPNO	10
PROCNO	1

F2 - Acquisition Parameters

Date_	20060712
Time	12.45
INSTRUM	dpx300
PROBHD	5 mm QNP 1H/13
PULPROG	zg30
TD	48848
SOLVENT	CDCl3
NS	64
DS	2
SWH	7183.908 Hz
FIDRES	0.147067 Hz
AQ	3.3998709 sec
RG	64
DW	69.600 usec
DE	6.00 usec
TE	299.2 K
D1	6.59999990 sec
MCREST	0.00000000 sec
MCWRK	0.01500000 sec

======== CHANNEL f1 ========

NUC1	1H
P1	9.40 usec
PL1	0.00 dB
SFO1	300.1330010 MHz

F2 - Processing parameters

SI	65536
SF	300.1300146 MHz
WDW	EM
SSB	0
LB	0.30 Hz
GB	0
PC	2.00

10383450, RX0002/38/3, BRUKER DPX300, QNP, H1 SPECTRUM, SOLV:-CDCL3/TMS

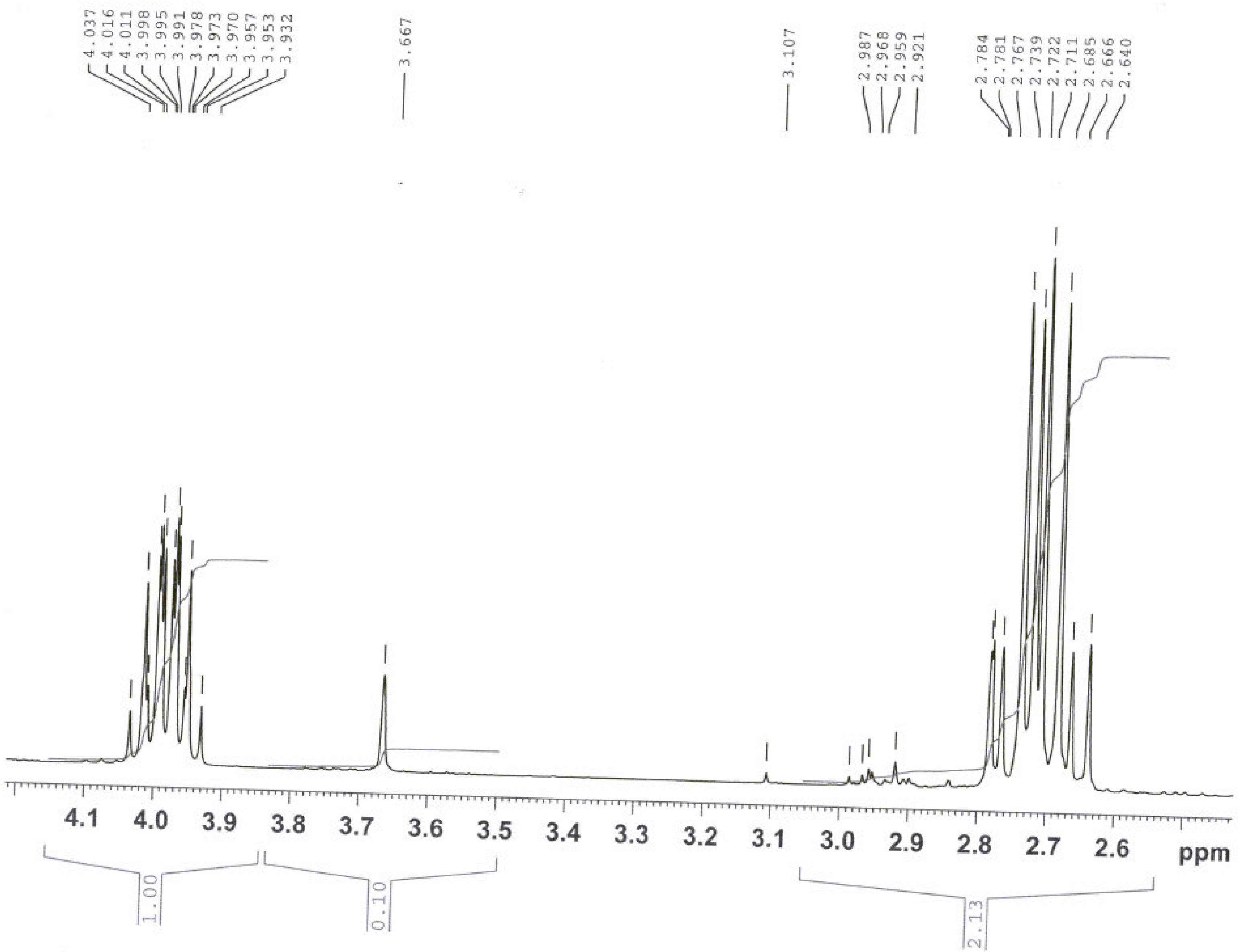

Intertek ASG

Current Data Parameters

NAME	10383450
EXPNO	10
PROCNO	1

F2 - Acquisition Parameters

Date_	20060712
Time	12.45
INSTRUM	dpx300
PROBHD	5 mm QNP 1H/13
PULPROG	zg30
TD	48848
SOLVENT	CDCl3
NS	64
DS	2
SWH	7183.908 Hz
FIDRES	0.147067 Hz
AQ	3.3998709 sec
RG	64
DW	69.600 usec
DE	6.00 usec
TE	299.2 K
D1	6.59999990 sec
MCREST	0.00000000 sec
MCWRK	0.01500000 sec

======== CHANNEL f1 ========

NUC1	1H
P1	9.40 usec
PL1	0.00 dB
SFO1	300.1330010 MHz

F2 - Processing parameters

SI	65536
SF	300.1300146 MHz
WDW	EM
SSB	0
LB	0.30 Hz
GB	0
PC	2.00

D. Abet

10383450, RX0002/38/3, BRUKER DPX300, QNP, H1 SPECTRUM, SOLV:-CDCL3/TMS

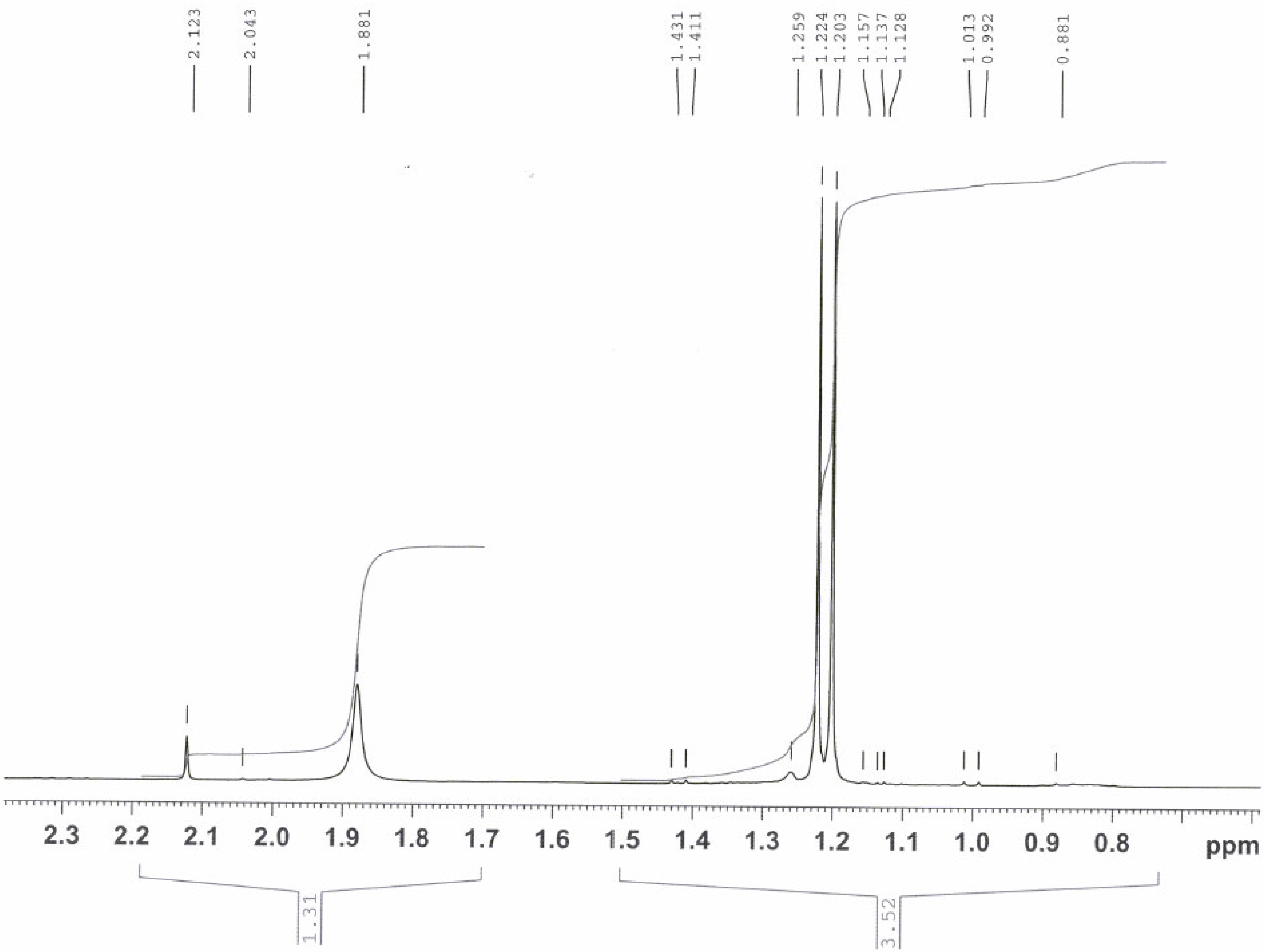

Intertek ASG

Current Data Parameters		
NAME	10383450	
EXPNO	10	
PROCNO	1	
F2 - Acquisition Parameters		
Date_	20060712	
Time	12.45	
INSTRUM	dpx300	
PROBHD	5 mm QNP 1H/13	
PULPROG	zg30	
TD	48848	
SOLVENT	CDCl3	
NS	64	
DS	2	
SWH	7183.908	Hz
FIDRES	0.147067	Hz
AQ	3.3998709	sec
RG	64	
DW	69.600	usec
DE	6.00	usec
TE	299.2	K
D1	6.59999990	sec
MCREST	0.00000000	sec
MCWRK	0.01500000	sec
======== CHANNEL f1 ========		
NUC1	1H	
P1	9.40	usec
PL1	0.00	dB
SFO1	300.1330010	MHz
F2 - Processing parameters		
SI	65536	
SF	300.1300146	MHz
WDW	EM	
SSB	0	
LB	0.30	Hz
GB	0	
PC	2.00	

10383691, RX0002/44C, BRUKER DPX300, QNP, H1 SPECTRUM, SOLV:-CDCL3/TMS

– OH

Intertek ASG

```
;zg30
;avance-version (02/05/31)
;1D sequence
;using 30 degree flip angle

#include <Avance.incl>

1 ze
2 30m
  d1
  p1*0.33 ph1
  go=2 ph31
  30m mc #0 to 2 F0(zd)
exit

ph1=0 2 2 0 1 3 3 1
ph31=0 2 2 0 1 3 3 1

;pl1 : f1 channel - power leve
;p1 : f1 channel -  90 degree
;d1 : relaxation delay; 1-5 *
```

TABLE 4 ENTRY 4

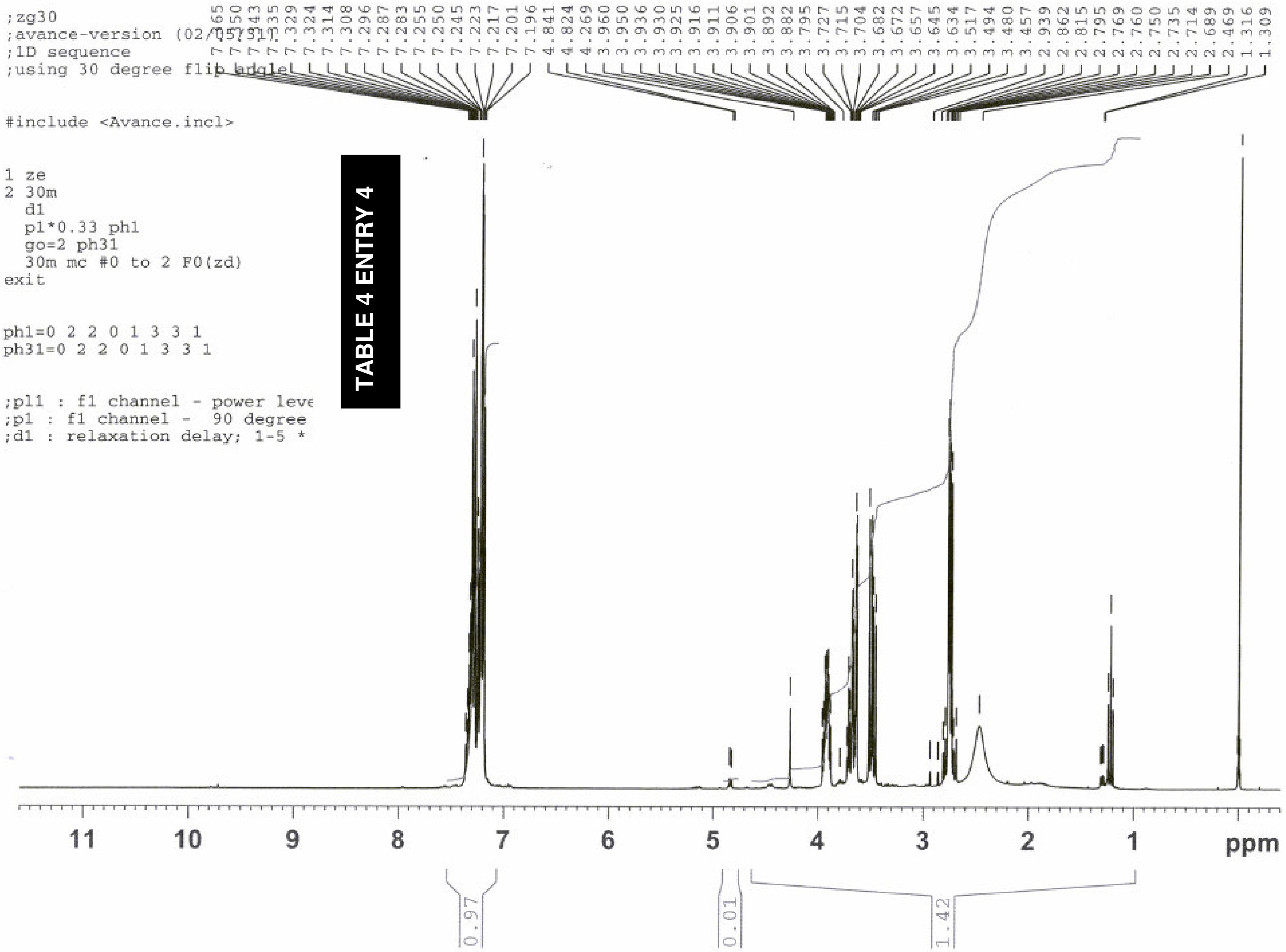

Current Data Parameters		
NAME	10383691	
EXPNO	10	
PROCNO	1	
F2 - Acquisition Parameters		
Date_	20060719	
Time	12.35	
INSTRUM	dpx300	
PROBHD	5 mm QNP 1H/13	
PULPROG	zg30	
TD	48848	
SOLVENT	CDCl3	
NS	64	
DS	2	
SWH	7183.908	Hz
FIDRES	0.147067	Hz
AQ	3.3998709	sec
RG	322.5	
DW	69.600	usec
DE	6.00	usec
TE	299.2	K
D1	6.59999990	sec
MCREST	0.00000000	sec
MCWRK	0.01500000	sec
======== CHANNEL f1 ========		
NUC1	1H	
P1	9.40	usec
PL1	0.00	dB
SFO1	300.1330010	MHz
F2 - Processing parameters		
SI	65536	
SF	300.1300075	MHz
WDW	EM	
SSB	0	
LB	0.30	Hz
GB	0	
PC	2.00	

D. Nat
19/7/06
